# Iron‐ and Cobalt‐Catalyzed Synthesis of Carbene Phosphinidenes

**DOI:** 10.1002/anie.201508303

**Published:** 2015-12-08

**Authors:** Kuntal Pal, Oliver B. Hemming, Benjamin M. Day, Thomas Pugh, David J. Evans, Richard A. Layfield

**Affiliations:** ^1^ School of ChemistryThe University of ManchesterOxford RoadManchesterM13 9PLUK; ^2^ Department of ChemistryUniversity of HullHullHU6 7RXUK

**Keywords:** catalysis, cobalt, iron, N-heterocyclic carbenes, phosphorus

## Abstract

In the presence of stoichiometric or catalytic amounts of [M{N(SiMe_3_)_2_}_2_] (M=Fe, Co), N‐heterocyclic carbenes (NHCs) react with primary phosphines to give a series of carbene phosphinidenes of the type (NHC)⋅PAr. The formation of (IMe_4_)⋅PMes (Mes=mesityl) is also catalyzed by the phosphinidene‐bridged complex [(IMe_4_)_2_Fe(μ‐PMes)]_2_, which provides evidence for metal‐catalyzed phosphinidene transfer.

Transition‐metal complexes of terminal phosphinidene ligands have stimulated considerable interest in recent years because of the fundamental interest in metal–phosphorus multiple bonds and because such complexes can serve as versatile organophosphorus reagents.^1^, ^2^, ^3^, ^4^, ^5^ Complexes of the general type [L_*n*_M=PR] have been synthesized with many transition metals, and the ability of the phosphinidene ligand to display electrophilic or nucleophilic characteristics has enabled a variety of phosphinidene‐transfer reactions. While much of this chemistry was pioneered with 4 d and 5 d transition metals,^4^, ^5^ several important studies involving 3 d metals have also been described.^6^, ^7^ Of particular significance is the three‐coordinate nickel(II) phosphinidene complex [(dtbpe)Ni=P(C_6_H_3_‐1,2‐Mes_2_)] (dtbpe=di‐*tert*‐butylphosphinoethane, Mes=mesityl), which is able to transfer the {PR} group to alkenes and alkynes in a stoichiometric manner, thus resulting in the formation of phosphorus heterocycles such as phosphiranes and phosphirenes.^6^

Our interest in low‐coordinate transition‐metal chemistry has focused on iron and cobalt complexes of N‐heterocyclic carbene (NHC) ligands.^8^, ^9^, ^10^, ^11^, ^12^ NHC‐stabilized iron and cobalt complexes of terminal or bridging phosphinidene ligands are currently unknown, hence we are interested in developing the phosphinidene‐transfer chemistry of complexes of the type [(NHC)_*n*_M(PR)] (M=Fe, Co). We now report that NHC‐ligated iron and cobalt phosphinidene complexes can indeed be synthesized, and they also show a strong tendency to couple the NHC to the phosphinidene group, which has allowed us to develop the first catalytic synthesis of carbene phosphinidenes, that is, compounds of the type (NHC)⋅PR.

To obtain the desired metal phosphinidene complexes, the three‐coordinate complexes [(IMe_4_)M(N′′)_2_] (IMe_4_=1,3,4,5‐tetramethylimidazolin‐2‐ylidene), with M=Fe (**1_Fe_**) or Co (**1_Co_**), were first synthesized and isolated (Scheme 1). The structures of **1_Fe_** and **1_Co_** (Figure 1; see Table S1 in the Supporting Information) are very similar to those of previously reported [(NHC)M(N′′)_2_] complexes.^8^, ^9^ The reactions of **1_Fe_** and **1_Co_** with MesPH_2_ in toluene produced dark green solutions, from which green crystals of the phosphinidene‐bridged dimetallic compounds [(IMe_4_)_2_M(μ‐PMes)]_2_⋅toluene were obtained (**2_Fe_**⋅toluene and **2_Co_**⋅toluene). The compounds **2_Fe_**⋅toluene and **2_Co_**⋅toluene can also be synthesized by combining IMe_4_, [M(N′′)_2_], and MesPH_2_ in the observed 2:1:1 stoichiometry, and hence isolated in yields of 45 % and 57 %, respectively. The structures of **2_Fe_** and **2_Co_** are centrosymmetric dimers in which the four‐coordinate metal centers occupy distorted tetrahedral environments, and are bonded to two terminal IMe_4_ ligands and two bridging phosphinidene ligands (Figure 2). In **2_Fe_**, the Fe‐P distances are 2.1280(9) Å and 2.126(1) Å and the Fe‐C1 and Fe‐C8 distances are 1.922(8) and 1.926(3) Å, respectively. The angles subtended at iron in **2_Fe_** lie in the 92.4(1)–133.7(1)° range. The phosphorus atoms in **2_Fe_** adopt pyramidal geometries and reside 0.454(2) Å out of the Fe_2_C15 plane, with an Fe‐P‐Fe angle of 75.99(3)°. Both Co‐P distances in **2_Co_** are 2.163(1) Å, hence they are slightly longer than the analogous distances in **2_Fe_**, and the Co‐C1 and Co‐C8 distances of 1.895(3) and 1.901(3) Å, respectively, are slightly shorter than the analogous distances in **2_Fe_**. The angles around cobalt in **2_Co_** also lie in a broad range of 93.9(2)–130.7(1). The phosphorus atoms in **2_Co_** reside 0.698(2) Å out of the Co_2_C15 plane, and the Co‐P‐Co angle is 71.39(3)°.

**Figure 1:**
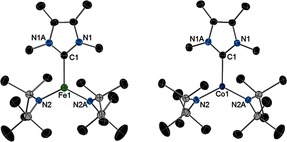
Molecular structures of **1_Fe_** and **1_FCo_** (thermal ellipsoids at 50 % probability). Hydrogen atoms are not shown. For unlabeled atoms: C black, and Si grey.

**Figure 2:**
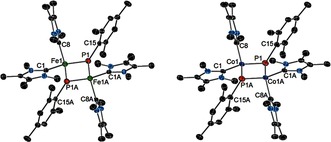
Molecular structures of **2_Fe_** and **2_Co_** (thermal ellipsoids at 50 % probability). Hydrogen atoms are not shown. For unlabeled atoms: C black and N light blue.

**Scheme 1:**
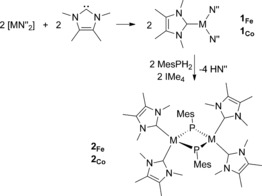
Synthesis of the compounds **1** and **2**. M=Fe or Co, N′′= N(SiMe_3_)_2_.

The formulation of the bridging phosphorus ligands in **2_Fe_** and **2_Co_** as phosphinidenes was made on the basis of their ^1^H‐coupled ^31^P NMR spectra (see Figures S12 and S19), which display singlets at *δ*=329.9 and 449.1 ppm, respectively. Furthermore, the IR spectra of the two compounds do not show any characteristic P−H stretches (see Figures S13 and S20).

The variable‐temperature ^1^H NMR spectrum of **2_Fe_** (see Figures S8–S11) in the range 193–323 K shows that the mesityl substituents are fluxional by virtue of rotation about the P−C bonds, and that the IMe_4_ ligands rotate around the Fe−C bonds. In the case of the mesityl substituents, the solid‐state structure of **2_Fe_** shows that there are two distinct environments for the *meta* CH protons. Both environments are observed in the ^1^H NMR spectrum at 193 K, with *δ*=6.05 and 6.58 ppm, and the two signals coalesce around 223 K. The ^1^H NMR spectrum of **2_Co_** toluene in [D_6_]benzene at 343 K shows resonances resulting from the mesityl *meta* CH protons at *δ*=6.71 ppm, and the mesityl *ortho*‐ and *para*‐methyl groups at 2.57 ppm and 2.07 ppm, respectively. The non‐equivalent IMe_4_ N‐methyl substituents occur at *δ*=4.77 and 3.30 ppm, and the non‐equivalent IMe_4_ backbone methyl groups occur at *δ*=1.83 and 1.31 ppm. Upon lowering the temperature, the resonances for the mesityl *meta* CH protons and the *ortho*‐methyl groups broaden in a similar manner to that of **2_Fe_**, thus suggesting similar fluxionality of the mesityl substituents and the IMe_4_ ligands, however the poor solubility of **2_Co_** precluded further investigations at lower temperatures.

An intriguing feature of the ^1^H NMR spectra of **2_Fe_**⋅toluene and **2_Co_**⋅toluene is that the resonances occur in the region typical of a diamagnetic compound. Using the Evans NMR method in solution,^13^ effective magnetic moments of zero were recorded for **2_Fe_** and **2_Co_**. The temperature dependence of the magnetic susceptibility was also measured for both compounds in a SQUID magnetometer in the temperature range 2–300 K using an applied field of 10 kOe. In both cases, negative values for the susceptibility were recorded, and is a clear indication of their diamagnetism. The most probable explanation for the diamagnetism is extremely strong antiferromagnetic exchange between the metal centers through the μ‐phosphinidene ligands.

To obtain iron and cobalt complexes with terminal phosphinidene ligands, IMe_4_ was replaced with the bulkier carbene 1,3‐dimesitylimidazolin‐2‐ylidene (IMes). Surprisingly, the reaction of [(IMes)Fe(N′′)_2_]^9^ with MesPH_2_ at 80 °C produced the carbene phosphinidene (IMes)⋅PMes (**3**) in 67 % yield (Scheme 2). Similarly, [(IPr)Fe(N′′)_2_] and MesPH_2_ reacted to give (IPr)⋅PMes (**4**) in 57 % yield (IPr=1,3‐bis(2,6‐diisopropyl)phenylimidazolin‐2‐ylidene). Intrigued by these observations, we analyzed the ^31^P NMR spectrum of the reaction mixture which produced **2_Fe_**, and found additional minor resonances at *δ*=−75.3 and −93.4 ppm, which correspond to (IMe_4_)⋅PMes (**5**) and Mes_2_PH, respectively (see Figure S16). The ^31^P NMR spectra of the reactions of [(IPr)Fe(N′′)_2_] and [(IMes)Fe(N′′)_2_] with MesPH_2_ also show minor amounts of Mes_2_PH in addition to **3** and **4**, however no evidence for metal phosphinidene species was found.

**Scheme 2:**
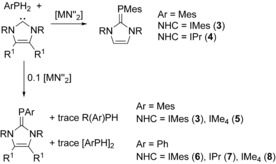
Stoichiometric and catalytic synthesis of **3**–**8**.

The direct reaction of 1,3‐diisopropylimidazolin‐2‐ylidene (*i*Pr_2_Im) with phenylphosphine or *p*‐tolylphosphine above 100 °C was recently reported by Radius et al. to give (*i*Pr_2_Im)⋅PAr.^14^ It is possible that **3**–**5** could have formed as a result of the NHCs reacting directly with MesPH_2_, a reaction we investigated by heating the NHC/MesPH_2_ mixtures at 80 °C in [D_6_]benzene for seven days. For the combinations of IMes/MesPH_2_ (see Figures S28 and S29) and IPr/MesPH_2_ (see Figures S30 and S31), no reaction was observed by either ^1^H or ^31^P NMR spectroscopy. The ^1^H and ^31^P NMR spectra of the IMe_4_/MesPH_2_ combination show that MesPH_2_, MesP(H)Me, and IMe_4_⋅PMes are present in an approximate ratio of 7:1:1, hence the mixture is dominated by starting materials (see Figures S32 and S33).

Formation of **3** and **4** is therefore at least assisted by [Fe(N′′)_2_]. The possibility that the reactions could be iron‐catalyzed was explored by treating NHC/MesPH_2_ mixtures with 10 mol % [Fe(N′′)_2_] (Scheme 2, Table 1). Remarkably, and although a temperature of 80 °C and a reaction time of five days were required, **3** was isolated in a yield of 71 %, and no unreacted phosphine was detected by ^31^P NMR spectroscopy (see Figure S35). In contrast, when 1:1 mixtures of IPr and MesPH_2_ were heated at 80 °C for seven days with 10 mol % [Fe(N′′)_2_] only a small amount of **4** formed, with the resulting ^31^P NMR spectrum being dominated by unreacted MesPH_2_ and minor amounts of Mes_2_PH and the diphosphane P_2_H_2_Mes_2_ (see Figure S37). Heating the IMe_4_/MesPH_2_ combination to 80 °C for seven days in the presence of 10 mol % [Fe(N′′)_2_] produced a roughly equimolar mixture of **5**, IMe_4_, and MesPH_2_ (see Figures S38 and S39), although here it was not possible to purify **5**.

**Table 1:** [M(N′′)_2_]‐catalyzed synthesis of **3**–**8** (M=Fe, Co).^[a]^

(NHC)⋅PAr	R	R^1^	Ar		*t*			Yield [%]^[c]^	
					Fe	Co		Fe	Co
**3**	Mes	H	Mes		7 d	7 d		71	51
**4**	Dipp	H	Mes		7 d	7 d		0	0
**5**	Me	Me	Mes		7 d	7 d		40^[d]^	32
**6**	Mes	H	Ph		2 d	2 h		46	58
**6** ^[b]^	Mes	H	Ph		–	18 h		–	61
**7**	Dipp	H	Ph		7 d	7 d		30^[d]^	50
**8**	Me	Me	Ph		7 d	4 h		41	62

[a] Reaction conditions: 10 mol % [M(N′′)_2_], toluene, 80 °C. [b] 1 mol % [Co(N′′)_2_]. [c] Yield of isolated product unless otherwise stated. [d] Yield determined by ^1^H NMR spectroscopy.

It was also possible to couple IMe_4_ and IMes with phenylphosphine using 10 mol % [Fe(N′′)_2_], thus producing (IMes)⋅PPh (**6**) and (IMe_4_)⋅PPh (**8**) in yields of 46 % and 41 %, respectively (Table 1). Selectivity for **8** in the presence of [Fe(N′′)_2_] is very good, and no unreacted PhPH_2_ is observed. The minor phosphorus‐containing by‐products were Ph_2_PH, and a trace amount of P_5_Ph_5_ was also found in the reaction leading to **6** (see Figures S40, S41, S44, and S45). The IMes/PhPH_2_ control experiment shows that the carbene does react directly with the phosphine to give **6**, but also that significant amounts of unreacted phosphine remain after heating at 80 °C for three days (see Figures S46 and S47), whereas in the iron‐catalyzed reaction all the PhPH_2_ is consumed. The IMe_4_/PhPH_2_ control experiment at 80 °C over three days produces a ^31^P NMR spectrum dominated by unreacted phosphine and small amounts of **8**, P_4_Ph_4_, P_5_Ph_5_, and P_2_H_2_Ph_2_ (see Figures S50 and S51). The reaction between IPr and PhPH_2_ in the presence of [Fe(N′′)_2_] produced an approximately equimolar mixture of IPr, (IPr)⋅PPh (**7**), and IPr(H)_2_ in addition to small amounts of P_2_H_2_Ph_2_ (see Figures S42 and S43). The IPr/PhPH_2_ control experiment produced an ^1^H NMR spectrum which consisted mainly of unreacted starting materials, with IPr, **7**, and IPr(H)_2_ being present in an approximate 5:1:1 ratio, and the ^31^P NMR spectrum is dominated by unreacted PhPH_2_ (see Figures S48 and S49).

With 10 mol % loadings of [Co(N′′)_2_], the reactions between the NHCs and ArPH_2_ at 80 °C are generally more efficient than the analogous iron chemistry, thus allowing **3** and **5**–**8** to be isolated in yields of 51–62 % (Table 1). Reaction times at 80 °C can also be reduced to 2–4 hours for the synthesis of **6** and **8**, and **6** can be synthesized using 1 mol % [Co(N′′)_2_], albeit with a reaction time of 18 hours. Based on ^31^P NMR spectroscopy of the reaction mixtures, the selectivity for **3** and **6**–**8** is very good (see Figures S52, S53, and S56–S65). Only (IPr)⋅PMes (**4**) could not be synthesized using [Co(N′′)_2_], with only starting materials being detected after heating at 80 °C for seven days (see Figures S54 and S55). The unsuccessful catalyzed reactions of IPr with MesPH_2_ are likely to be a consequence of the IPr ligand undergoing a fast normal‐to‐abnormal rearrangement^8^ relative to the rate of (IPr)⋅PAr formation, presumably because of the steric bulk of IPr.

Carbene phosphinidenes were first reported in 1997,^15^ and recent studies have shown that these compounds,^16^ and closely related systems,^17^ are the subject of renewed interest. The compounds **3**–**5** are new members of the family. Much of the intrigue in this type of phospha‐alkene focuses on the “inversely polarized” nature of the phosphorus–carbon bond,^18^ which has been considered to have three resonance forms, one with a formal C=P double bond, a zwitterionic form with a C−P single bond, and a third form with a C→P donor–acceptor interaction (Scheme 3). The latter two forms have been invoked to rationalize the fact that the ^31^P NMR chemical shifts in carbene phosphinidenes occur much further upfield than in typical phospha‐alkenes. In agreement with this trend, the ^31^P NMR chemical shifts for **3**–**5** in [D_6_]benzene are *δ*=−59.1, −52.1, and −75.1 ppm, respectively. The compounds **3** and **4** also fit the observed trend of long C–P distances in carbene phosphinidenes,^15^, ^16^ with the distances being 1.769(3) and 1.766(2) Å, respectively (see Figure S67 and Table S3; diffraction‐quality crystals of **5** could not be obtained).

**Scheme 3:**
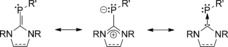
Resonance forms of carbene phosphinidenes.

Further evidence for C−P single bonds in **3** and **4** can be found in their ^1^H NMR spectra. The Dipp methine protons in **4** occur as a four‐proton septet at *δ*=3.13 ppm at 333 K, and decoalesce into two two‐proton septets at 233 K (*δ*=3.15 and 3.04 ppm). This observation indicates that the two Dipp substituents are inequivalent on the NMR timescale at 233 K, and is consistent with the solid‐state structure of **4**. The NMR spectra can be accounted for by rotation of the mesityl group around the C−P bond of **4**, and at lower temperatures it occupies a position *cis* to one Dipp substituent. The activation barrier for the rotation in **4** is estimated to be Δ*G* ^≠^=52.6 kJ mol^−1^. A similar process was also observed in the ^1^H NMR spectrum of **3**, with Δ*G* ^≠^=62.1 kJ mol^−1^.

Synthetic routes to carbene phosphinidenes include the ring‐opening reactions of NHCs with cyclic polyphosphanes,^15^ chloride displacement reactions of PhPCl_2_ with a range of carbenes followed by alkali metal reduction,16f and the defluorosilylation reaction of PhenoFluor with P(SiMe_3_)_3_.16c Several routes to carbene adducts of the parent phosphinidene (NHC)⋅PH are known,^19^ and the direct reaction of phenylphosphine and *p*‐tolylphosphine with *i*Pr_2_Im has been described.^14^ To the best of our knowledge, we have identified the first catalytic route to carbene phosphinidenes. Furthermore, the carbene phosphinidene syntheses described above represent the first catalytic phosphinidene transfer reactions.

Regarding the mechanism(s) through which the **3**–**8** form under [M(N′′)_2_] catalysis, we were interested to determine whether or not the phosphinidene complex **2_Fe_** could catalyze the formation of **5**. Thus, IMe_4_ and MesPH_2_ were combined with 5 mol % of **2_Fe_**⋅toluene and heated to 80 °C for seven days. The ^31^P NMR spectrum of the reaction (see Figure S66) shows that **5** is the major phosphorus‐containing product and that all MesPH_2_ was consumed. This observation indicates that a metal–phosphinidene species is involved in the formation of the carbene phosphinidenes, however, it provides no detailed insight into how the C−P bond is formed. Mechanistic possibilities include: phosphinidene transfer from the metal to the coordinated NHC, or vice‐versa; addition of NHC to coordinated phosphinidene; a concerted C−P bond‐forming process; or elimination of cyclic polyphosphanes which subsequently react with free NHC. These possibilities will be subjected to detailed analysis and reported in a future article. We will also focus on developing the phosphinidene‐transfer chemistry for the synthesis of organophosphorus heterocycles.

## Supporting information

As a service to our authors and readers, this journal provides supporting information supplied by the authors. Such materials are peer reviewed and may be re‐organized for online delivery, but are not copy‐edited or typeset. Technical support issues arising from supporting information (other than missing files) should be addressed to the authors.

Supplementary
