# Supplementary material for: Iron‐ and Cobalt‐Catalyzed Synthesis of Carbene Phosphinidenes
Source: Angew Chem Int Ed Engl. 2015 Dec 8;55(5):1690–3. doi: 10.1002/anie.201508303 (PMC5064616; doi:10.1002/anie.201508303)
Supplement: Supplementary file 1 — Supplementary [file ANIE-55-1690-s001.pdf]

Angewandte Chemie
Eine Zeitschrift der Gesellschaft Deutscher Chemiker

Supporting Information

Iron- and Cobalt-Catalyzed Synthesis of Carbene Phosphinidenes

*Kuntal Pal, Oliver B. Hemming, Benjamin M. Day, Thomas Pugh, David J. Evans, and Richard A. Layfield**

anie_201508303_sm_miscellaneous_information.pdf

General Experimental Details: All manipulations were performed under an atmosphere of dry, oxygen-free argon, using either standard Schlenk techniques or an argon-filled glove box. Toluene and THF, and their deuterated analogues for NMR spectroscopy, were dried by refluxing over potassium and collected by distillation. All solvents were stored over activated 4 Å molecular sieves or a potassium mirror and freeze-thaw degassed prior to use. Literature procedures were used for the synthesis of $[Fe\{N(SiMe_3)_2\}_2]_2$,[1] $[Co\{N(SiMe_3)_2\}_2]_2$,[2] IMe_4,[3] IMes,[4] IPr,[4] and $MesPH_2$.[5] Phenylphosphine was purchased as a 10% w/w solution in hexanes and used without further purification. Elemental analyses were carried out at London Metropolitan University, U.K. Infrared spectra were recorded as Nujol mulls in KBr discs on a Shimadzu IRAffinity-1S FT-IR spectrophotometer. Electronic absorption spectra were measured as toluene solutions (5.8×10^{-4} to 2.0×10^{-5} M) using a PerkinElmer Lambda-1050 spectrometer in the range 300-2000 nm. X-ray diffraction data were collected on an Oxford Instruments XCalibur2 diffractometer or an Agilent SuperNova, using MoKα radiation, or a Bruker APEX-II diffractometer, using CuKα radiation. NMR spectra were acquired on Bruker Avance-III 400 MHz or 500 MHz spectrometers. Mössbauer spectra were recorded in zero magnetic field at 80 K on an ES-Technology MS-105 Mössbauer spectrometer with a 900 MBq ^{57}Co source in a rhodium matrix at ambient temperature. Spectra were referenced against 25 μm iron foil at 298 K and spectrum parameters were obtained by fitting with Lorentzian curves. Samples were ground with boron nitride under a nitrogen atmosphere before mounting in the sample holder.

Synthesis of $[(IMe_4)Fe\{N(SiMe_3)_2\}_2]$ (1-Fe). A solution of IMe_4 (0.06 g, 0.5 mmol) in toluene (5 ml) was added to a solution of $[Fe\{N(SiMe_3)_2\}_2]_2$ (0.19 g, 0.25 mmol) in toluene (5 ml) at room temperature, and the mixture was stirred for three hours. The resulting pale-green solution was filtered and concentrated to a volume of 2 ml and stored at −28 °C overnight, resulting in the formation of pale green crystals of $[(IMe_4)Fe(N'')_2]$ (**1-Fe**). The crystals were isolated by filtration, washed with cold pentane and dried *in vacuo* (0.18 g, 73%). Anal. Calcd. for $C_{19}H_{48}FeN_4Si_4$: C, 45.48; H, 9.84; N, 11.17, Found: C, 45.61; H, 9.33; N, 11.28. Effective magnetic moment (Evans methods), $\mu_{eff} = 5.4(2)\ \mu_B$.

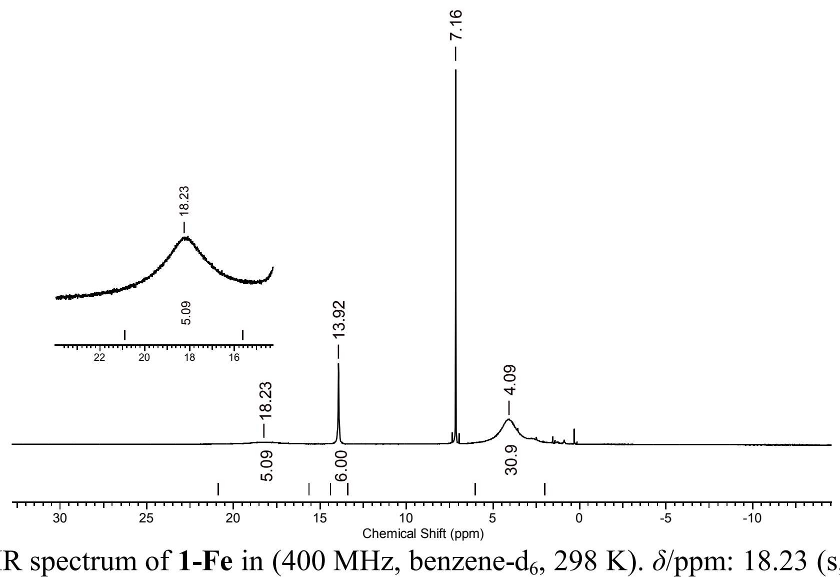

Figure S1. ^1H NMR spectrum of **1-Fe** in (400 MHz, benzene-d_6, 298 K). δ/ppm: 18.23 (s, 6H, CCH_3), 13.92 (s, 6H, NCH_3), 4.09 (s, 36H, $SiMe_3$).

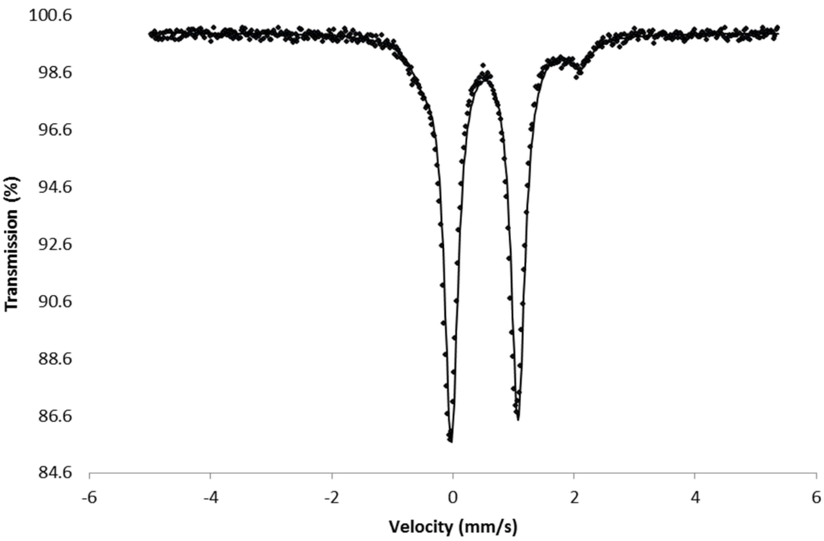

Figure S2. ^{57}Fe Mössbauer spectrum of **1-Fe** at 80 K. δ = 0.51 mm s^{-1} and ΔE_Q = 1.11 mm s^{-1} (hwhm = 0.14 mm s^{-1}). The spectrum also reveals the presence of a small amount (~2%) of a high-spin iron(II) impurity with δ = 0.79 mm s^{-1} and ΔE_Q = 2.58 mm s^{-1} (hwhm = 0.30 mm s^{-1}).

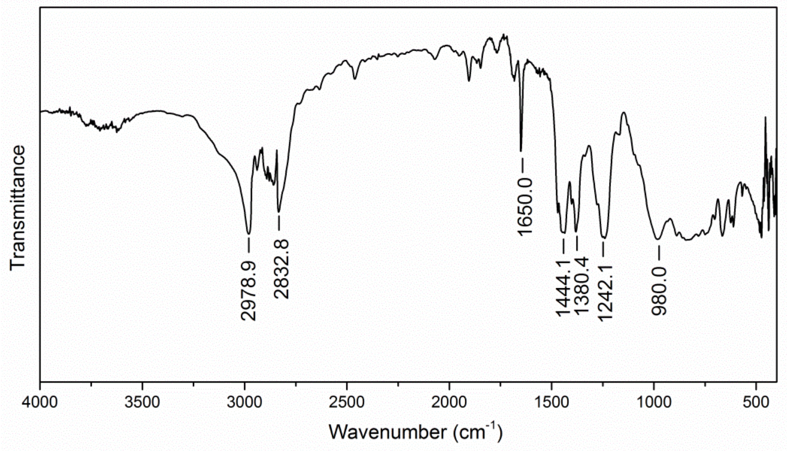

Figure S3. IR spectrum of **1-Fe**.

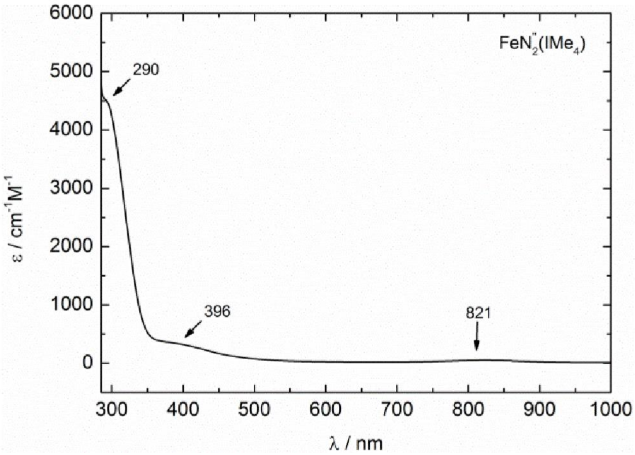

Figure S4. UV/vis spectrum of **1-Fe** in toluene.

Synthesis of [(IMe$_4$)Co{N(SiMe$_3$)$_2$}$_2$] (1-Co). Compound **1-Co** was synthesized using the procedure described for **1-Fe**, with IMe (0.12 g, 1.0 mmol) and [Co{N(SiMe$_3$)$_2$}$_2$]$_2$ (0.38 g, 0.5 mmol). **1-Co** was isolated as green crystals (0.39 g, 78%). Calculated elemental analysis for $C_{19}H_{48}CoN_4Si_4$: C 45.33, H 9.61, N 11.13. Found: C 44.95; H 9.69; N 10.93. Effective magnetic moment (Evans methods), $\mu_{eff} = 4.8(2)\ \mu_B$.

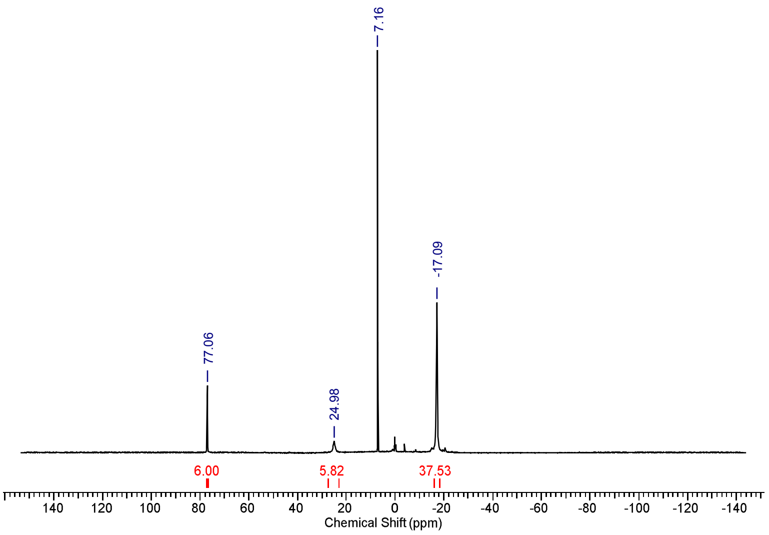

Figure S5. ^{1}H NMR spectrum of **1-Co** (400 MHz, benzene-d$_6$, 298 K). δ/ppm: 77.06 (6H, s, CCH$_3$); 24.98 (6H, s, NCH$_3$), –17.09 (36H, s, SiMe$_3$).

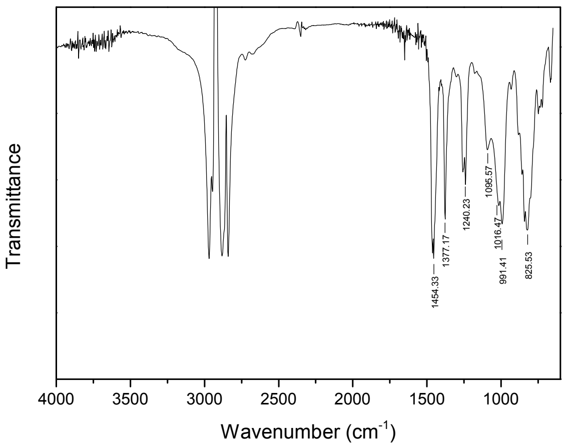

Figure S6. IR spectrum of **1-Co**.

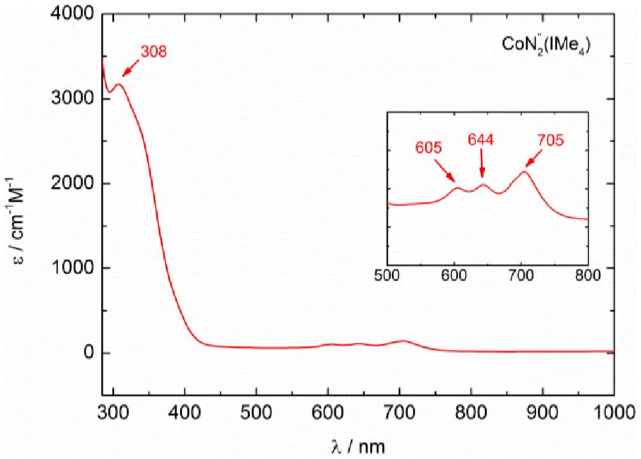

Figure S7. UV/vis spectrum of **1-Co** in toluene.

Synthesis of [(IMe$_4$)$_2$Fe{μ-PMes}]$_2$·toluene (2-Fe·toluene). A solution of IMe$_4$ (0.12 g, 1.0 mmol) and MesPH$_2$ (76 µL, 0.50 mmol) in toluene (10 ml) was added to a solution of [Fe{N(SiMe$_3$)$_2$}$_2$]$_2$ (0.19 g, 0.25 mmol) in toluene (10 ml) at room temperature, and the mixture was stirred at 80°C overnight. The resulting dark solution was filtered and concentrated to a volume of approximately 10 ml and stored at −28 °C overnight, which resulted in the formation of dark-green crystals of [(IMe$_4$)$_2$Fe(µ-PMes)$_2$]·toluene (**2-Fe**·toluene). The crystals were washed with a cold 1:1 mixture of toluene and pentane and then dried (0.113 g, 45% based on iron). Calculated elemental analysis for $C_{53}H_{78}Fe_2N_8P_2$: C 63.60, H 7.86, N 11.20. Found: C 63.56, H 8.01, N 11.08.

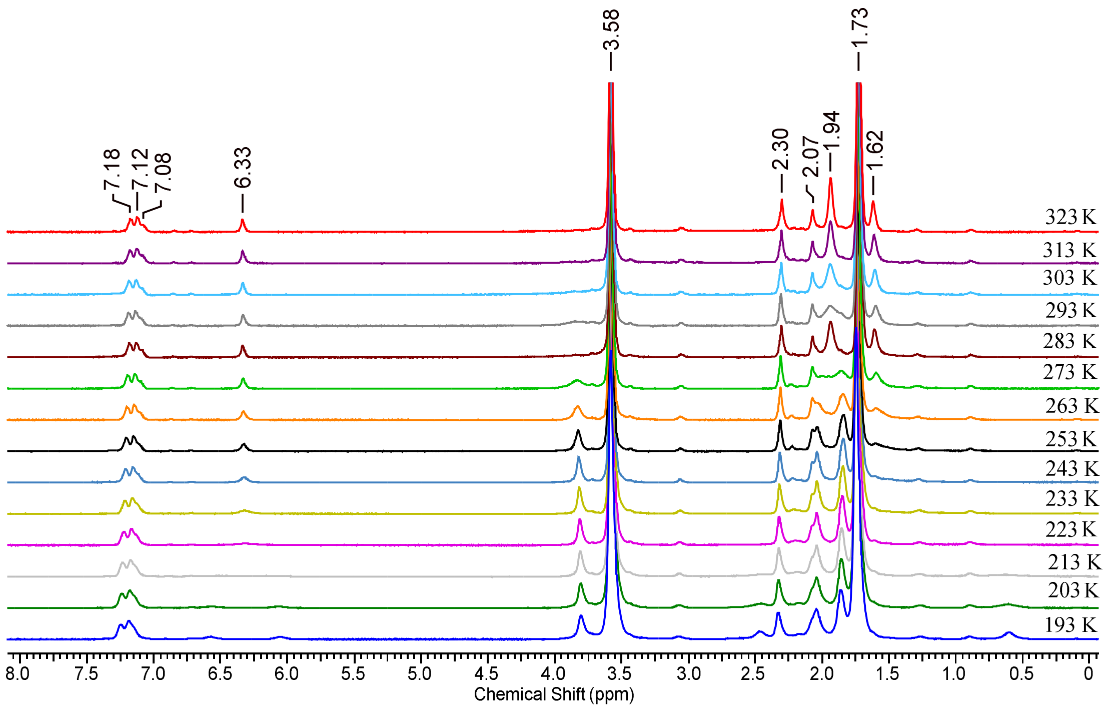

Figure S8. Variable-temperature ^{1}H NMR spectrum of **2-Fe**·toluene (500 MHz, thf-d$_8$).

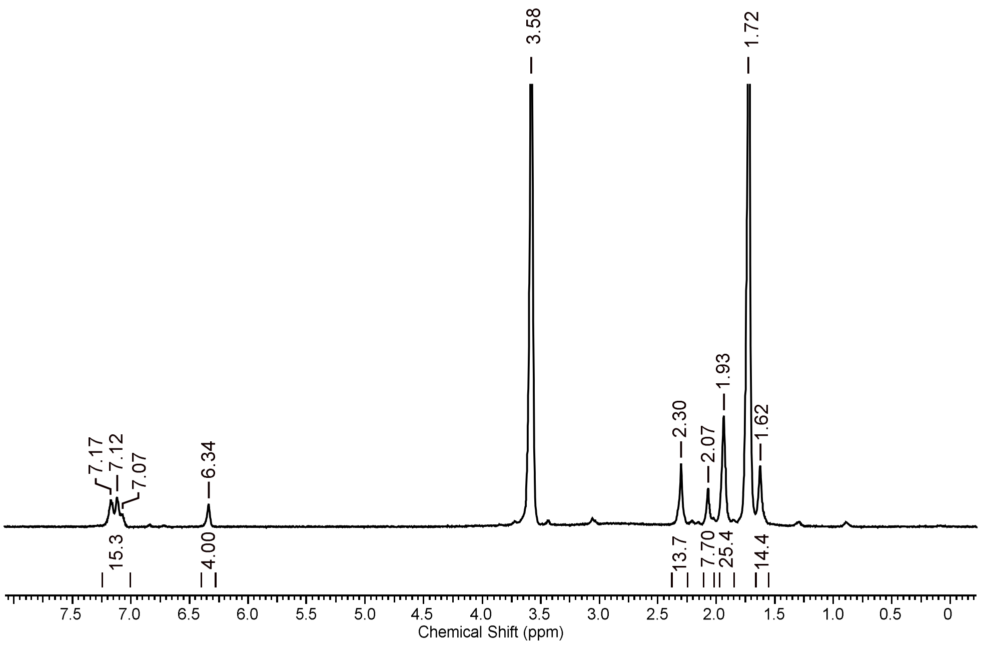

Figure S9. ^{1}H NMR spectrum of **2-Fe**·toluene (500 MHz, 323 K, thf-d$_8$). δ/ppm: 6.34 (4H, s, mesityl *meta*-CH); 2.07 (6H, s, mesityl *para*-CH$_3$); 1.93 (24H, s, IMe$_4$ CCH$_3$); 1.62 (12H, s, mesityl *ortho*-CH$_3$). Lattice toluene: 7.07-7.17 (m, aryl CH); 2.30 (s, Me). IMe$_4$ NCH$_3$ groups not observed.

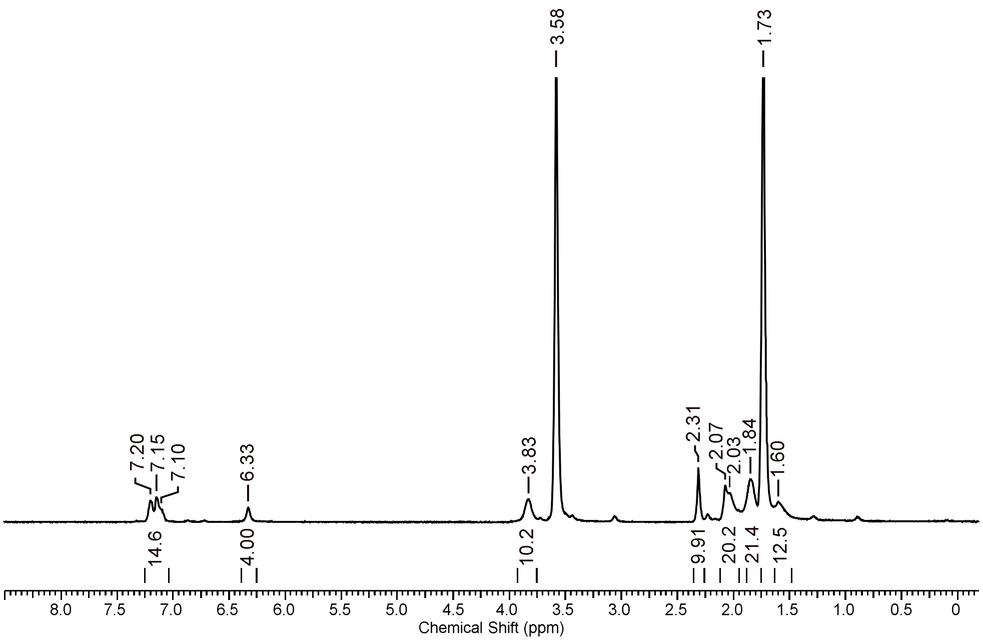

Figure S10. ^{1}H NMR spectrum of **2-Fe**·toluene in (500 MHz, thf-d_8, 263 K). δ/ppm: 6.33 (4H, s, mesityl *meta*-CH); 3.83(12H, s, IMe_4 NCH_3); 2.07 (6H, s, mesityl *para*-CH_3); 2.03 (12H, s, IMe_4 NCH_3); 1.84 (24H, s, IMe_4 CCH_3); 1.60 (12H, s, mesityl *ortho*-CH_3). Lattice toluene: 7.10-7.20 (m, aryl CH); 2.31 (s, Me).

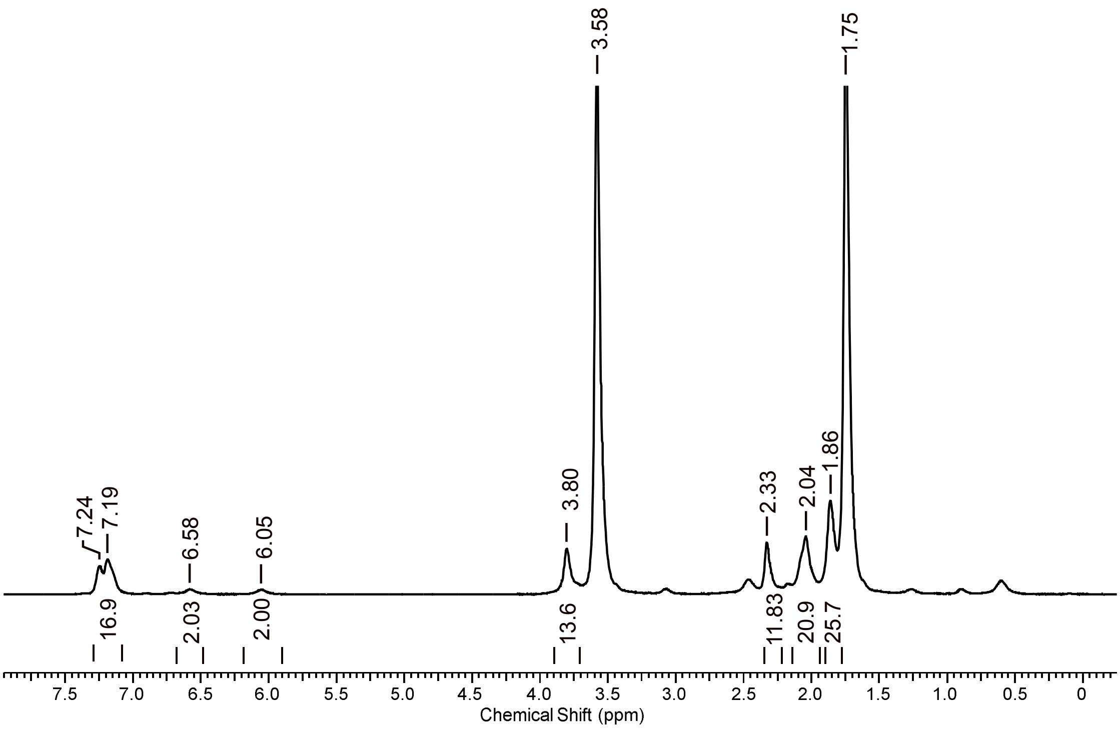

Figure S11. ^{1}H NMR spectrum of **2-Fe**·toluene in (500 MHz, thf-d_8, 193 K). δ/ppm: 6.58 (2H, s, mesityl *meta*-CH); 6.06 (2H, s, mesityl *meta*-CH); 3.80 (12H, s, IMe_4 NCH_3); 2.04 (6H, s, mesityl *para*-CH_3, and 12H IMe_4 NCH_3); 1.86 (24H, s, IMe_4 CCH_3). Lattice toluene: 7.07-7.18 (m, aryl CH); 2.33 (s, Me).

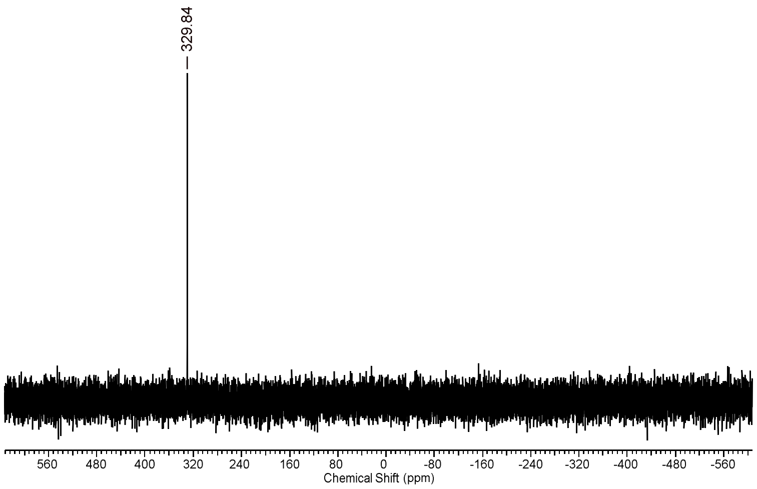

Figure S12. Proton-coupled ^{31}P NMR spectrum of **2-Fe**·toluene in thf-d_8 at 293 K.

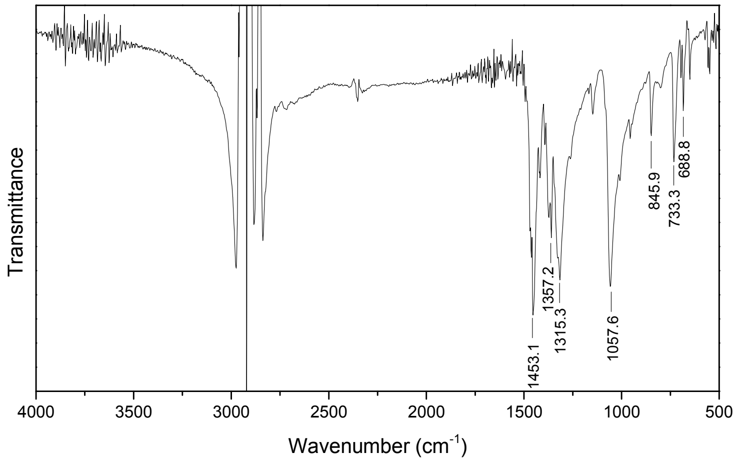

Figure S13. IR spectrum of **2-Fe**·toluene.

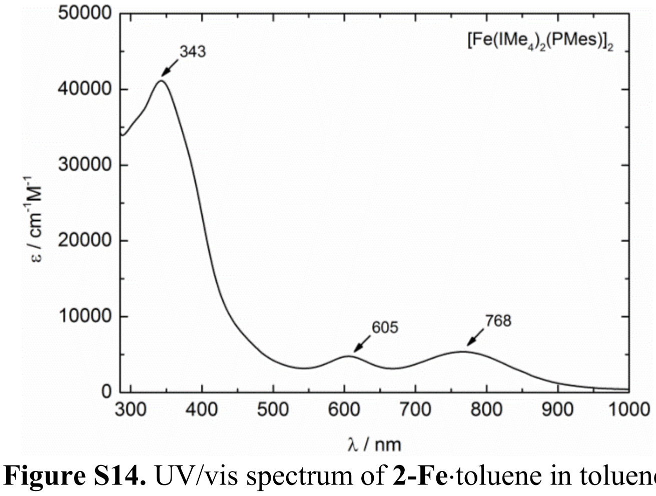

Figure S14. UV/vis spectrum of **2-Fe**·toluene in toluene.

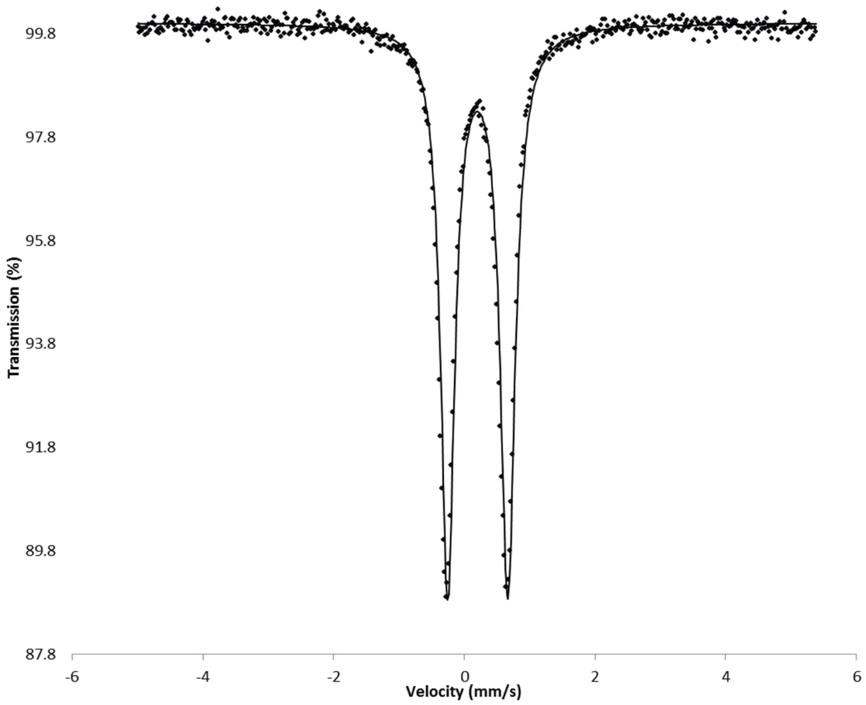

Figure S15. [57]Fe Mössbauer spectrum of **2-Fe**·toluene at 80 K. δ = 0.18 mm s^{-1} and ΔE_Q = 0.92 mm s^{-1} (hwhm = 0.14 mm s^{-1}).

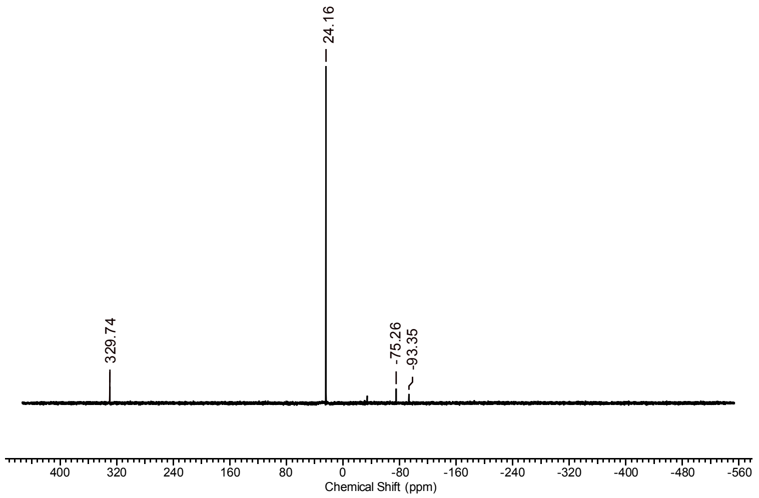

Figure S16. $^{31}P\{^1H\}$ NMR spectrum of the 1:1:1 reaction mixture of IMe_4, $MesPH_2$ and [Fe(N″)$_2$] after heating for three days at 80°C (benzene-d$_6$, 298 K). δ/ppm: +329.7 (**2-Fe**); –75.3 (IMe_4·PMes); –93.4 (Mes_2PH). δ = +24.2 ppm corresponds to a benzene solution of Ph_3P=O contained in a capillary.

Synthesis of [(IMe$_4$)$_2$Co{μ-PMes}]$_2$·toluene (2-Co·toluene). IMe_4 (0.124 g, 1.0 mmol) and [Co(N(SiMe$_3$)$_2$)$_2$]$_2$ (0.19 g, 0.25 mmol) were dissolved in toluene (8 ml) and stirred at room temperature for 1 hour. A solution of $MesPH_2$ in toluene (5.0 M, 0.1 ml, 0.5 mmol) was added and the reaction mixture was heated at 80°C for 16 hours. The solution was cooled to room temperature and concentrated *in vacuo* to a volume of approximately 3 ml. The solution was stored at –28°C overnight, resulting in the formation of dark green crystals. The nascent solvent was decanted and the crystals were washed with hexane (6 ml), and then dried *in vacuo*, giving **2-Co·toluene** as a green polycrystalline material (0.143 g, 57% based on cobalt). Calculated elemental analysis for $C_{53}H_{78}Co_2N_8P_2$: C 63.21, H 7.81, N 11.12. Found: C 63.13, H 7.92, N 11.03.

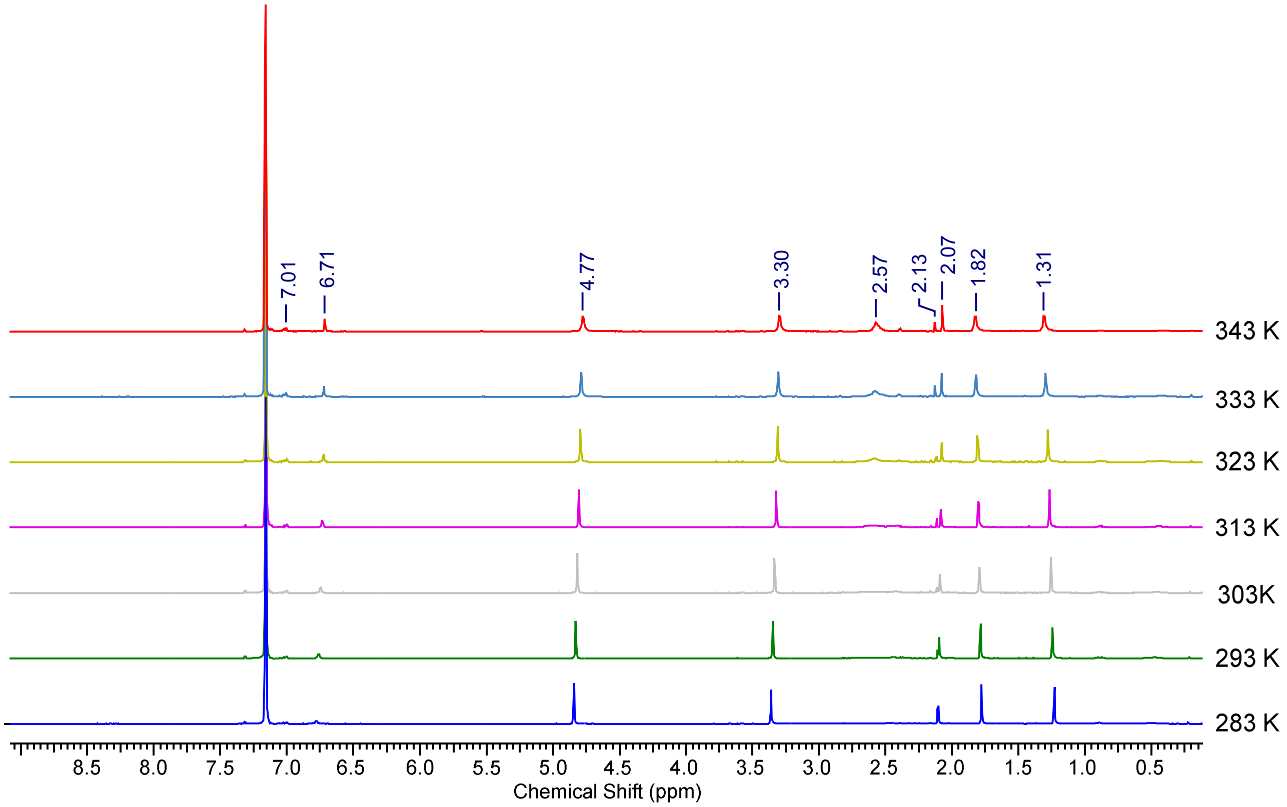

Figure S17. Variable-temperature ^{1}H NMR spectrum of **2-Co**·toluene in (500 MHz, benzene-d$_6$).

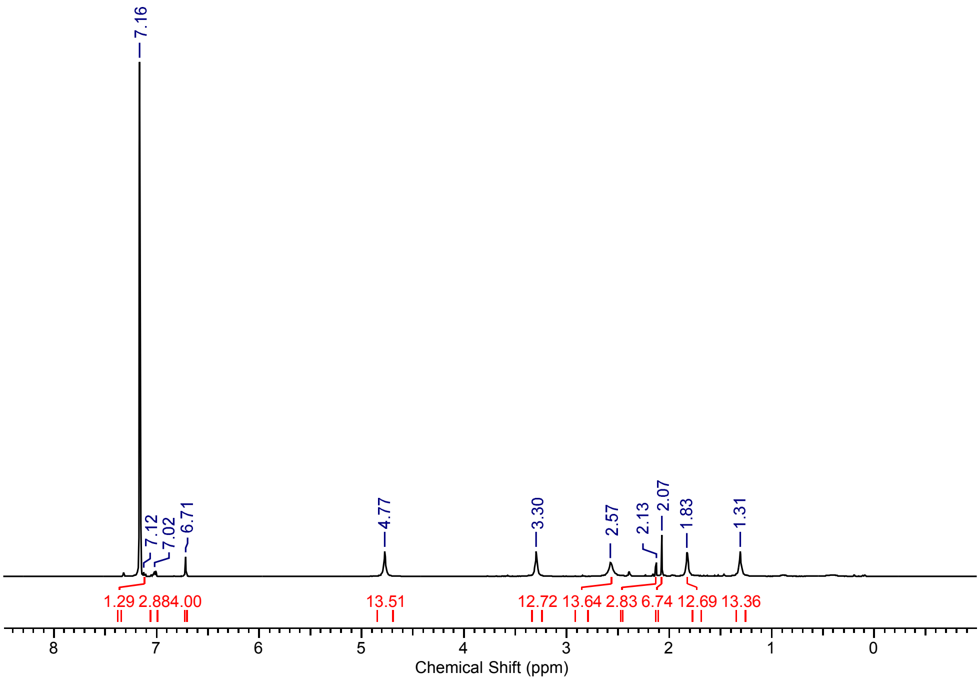

Figure S18. ^{1}H NMR spectrum of **2-Co**·toluene in benzene-d$_6$ (500 MHz, 353 K). *δ*/ppm: 6.71 (4H, s, mesityl *meta*-CH); 4.77 (12H, s, IMe$_4$ NCH$_3$); 3.30 (12H, s, IMe$_4$ NCH$_3$); 2.57 (12H, s, mesityl *ortho*-CH$_3$); 2.07 (6H, s, mesityl *para*-CH$_3$); 1.82 (12H, s, IMe$_4$ backbone CH$_3$); 1.30 (12H, s, IMe$_4$ CCH$_3$).

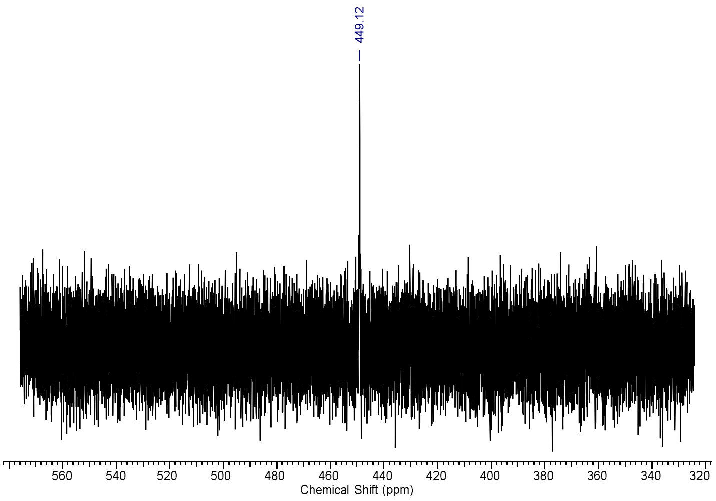

Figure S19. Proton-coupled ^{31}P NMR spectrum of **2-Co**·toluene in benzene-d_6 at 293 K.

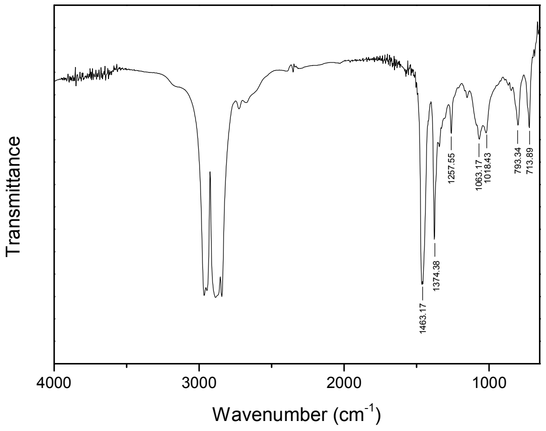

Figure S20. IR spectrum of **2-Co**·toluene.

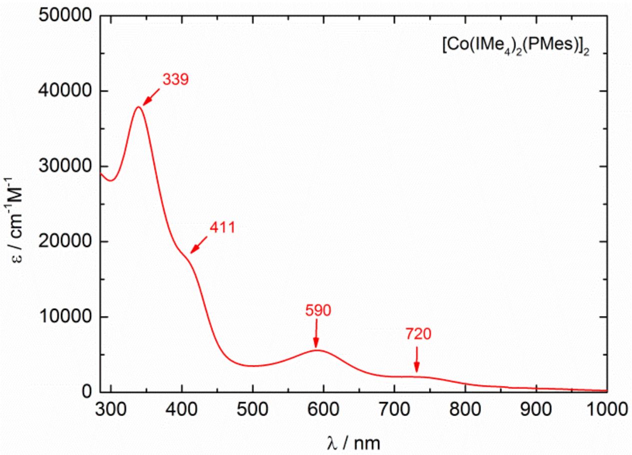

Figure S21. UV/vis spectrum of **2-Co**·toluene in toluene.

Synthesis of IMes·PMes (6) using one stoichiometric equivalent of [Fe(N'')$_2$]. A solution of IMes (0.15 g, 0.50 mmol) in toluene (8 ml) was added to a solution of [Fe{N(SiMe$_3$)$_2$}$_2$]$_2$ (0.19 g, 0.25 mmol) in toluene (2 ml) at room temperature and the mixture was stirred for 30 minutes. A solution of MesPH$_2$ (76 µL, 0.50 mmol) in toluene (2 ml) was added to the reaction mixture, which was stirred at 80°C for 30 min and then at room temperature overnight. The resulting solution was filtered, concentrated to a volume of 3 ml and stored

at –28°C overnight, which resulted in the formation of pale-yellow crystals. The crystals were isolated by filtration, washed with pentane and dried *in vacuo*. IMes·PMes was isolated as a pale yellow crystalline material (0.15 g, 67%). Anal. Calcd for : $C_{30}H_{35}N_2P$, 79.26; H, 7.76; N, 6.16, Found: C, 79.17; H, 7.64; N, 6.27.

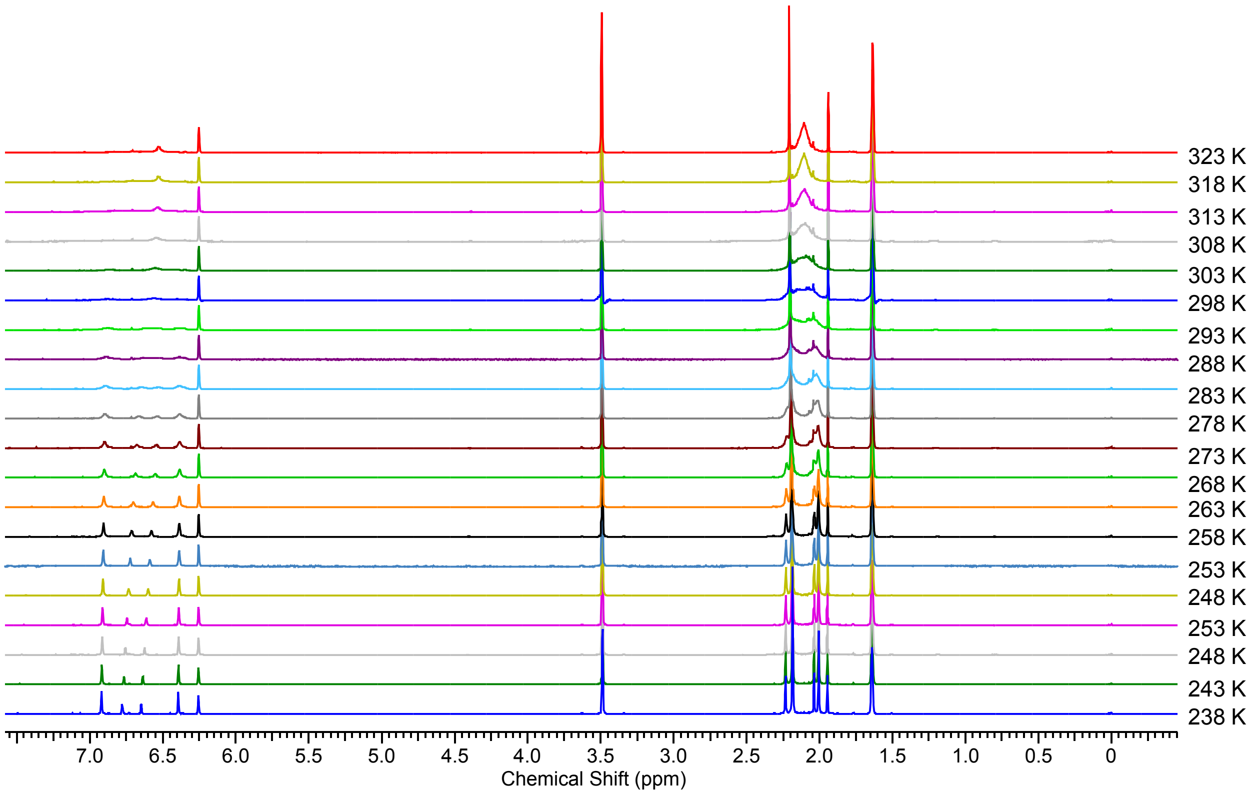

Figure S22. Variable-temperature ^{1}H NMR spectrum of IMes·PMes (**3**) in (500 MHz, THF-d_8). The coalescence temperature of the CH_3 resonances is approximately 303 K.

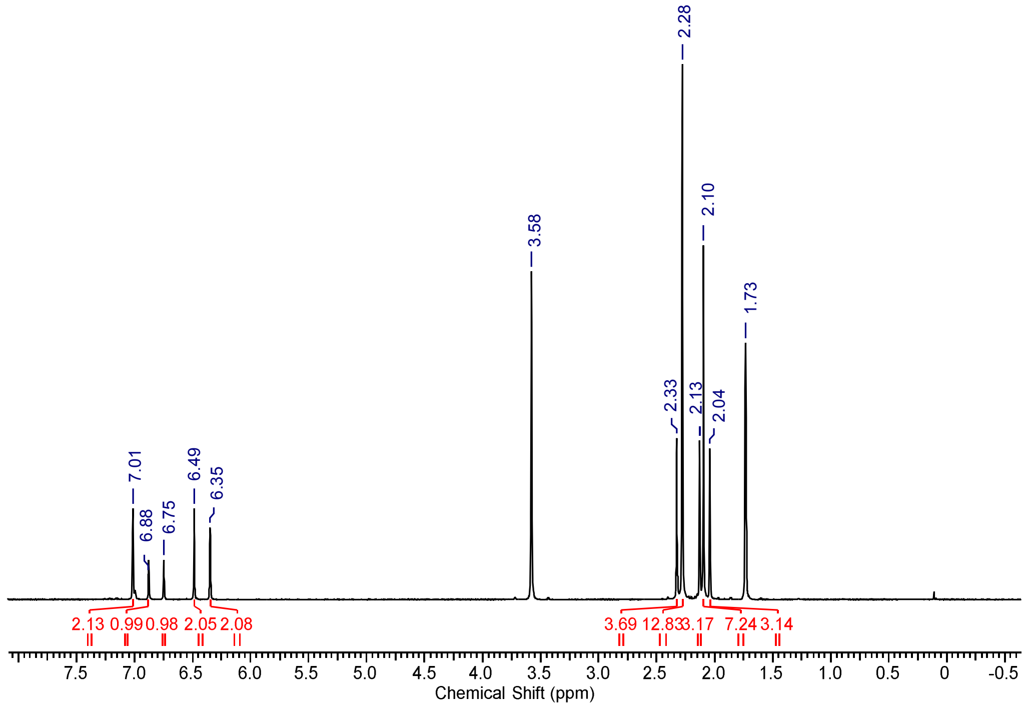

Figure S23. ^{1}H NMR spectrum of IMes·PMes (**3**) in (500 MHz, thf-d_8, 235 K). δ/ppm: 7.01 (2H, s, mesityl *meta*-CH); 6.88 (1H, t, imidazolylidene CH, $^3J_{HH}$ = 1.88 Hz); 6.75 (1H, t, imidazolylidene CH, $^3J_{HH}$ = 1.88 Hz); 6.49 (2H, s, mesityl *meta*-CH); 6.35 (2H, s, mesityl *meta*-CH); 2.33 (3H, s, mesityl *para*-CH_3); 2.28 (6H, s, mesityl *ortho*-CH_3); 2.28 (6H, s, mesityl *ortho*-CH_3); 2.13 (3H, s, mesityl *para*-CH_3); 2.10 (6H, s, mesityl *ortho*-CH_3); 2.04 (6H, s, mesityl *para*-CH_3).

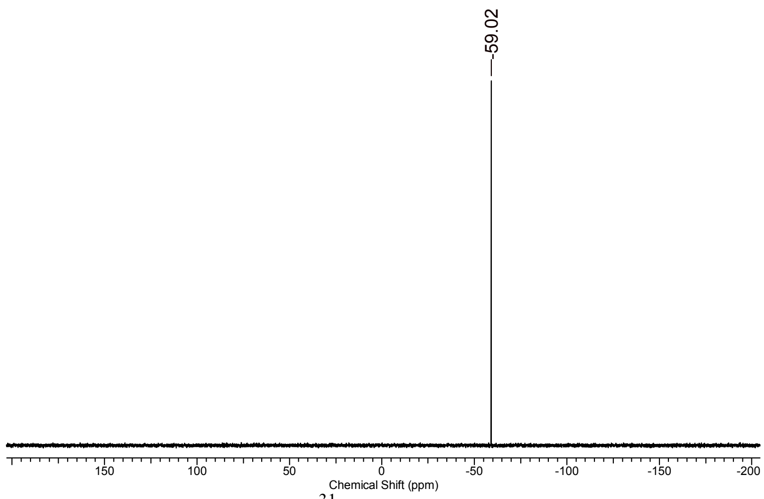

Figure S24. Proton-decoupled ^{31}P NMR spectrum of **3** in thf-d_8 at 293 K.

Synthesis of IPr·PMes (4) using one stoichiometric equivalent of [Fe(N'')$_2$]. A solution of IPr (0.19 g, 0.50 mmol) in toluene (8 ml) was added to [Fe{N(SiMe$_3$)$_2$}$_2$]$_2$ (0.19 g, 0.25 mmol) in toluene (2 ml) at room temperature to generate [(IPr)Fe(N(SiMe$_3$)$_2$)$_2$] *in situ*. A solution of MesPH$_2$ (76 µL, 0.50 mmol) in toluene (2 ml) was added and the reaction mixture was stirred at 60°C for 5 minutes and then at room temperature for two days. The resulting dark brown solution was filtered, concentrated to a volume of about 3 ml and stored at –28°C overnight, which resulted in the formation of yellow crystals. The nascent solvent was removed by filtration, and the crystals were washed with pentane and dried *in vacuo*. IPr·PMes was isolated as a yellow crystalline material (0.15 g, 57%). Anal. Calcd. for $C_{36}H_{47}N_2P$: C, 80.26; H, 8.79; N, 5.20, Found: C, 80.31; H, 8.89; N, 5.23.

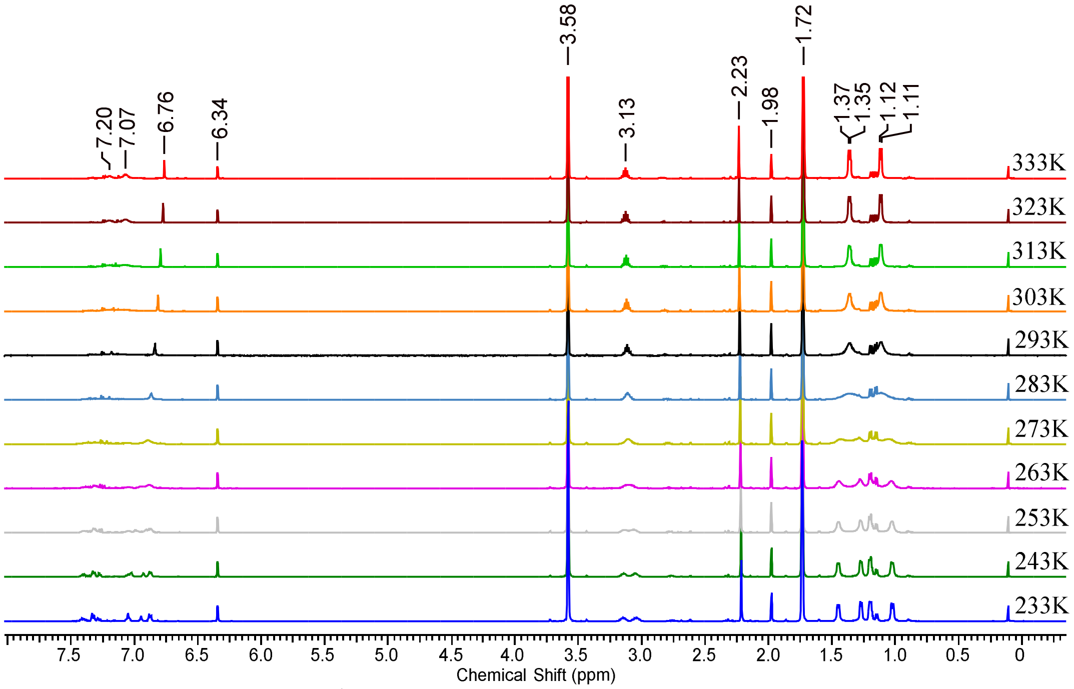

Figure S25. Variable-temperature ^{1}H NMR spectrum of **4** in (500 MHz, thf-d_8). The coalescence temperature of the ^{i}Pr methine (CH) resonances is approximately 258 K.

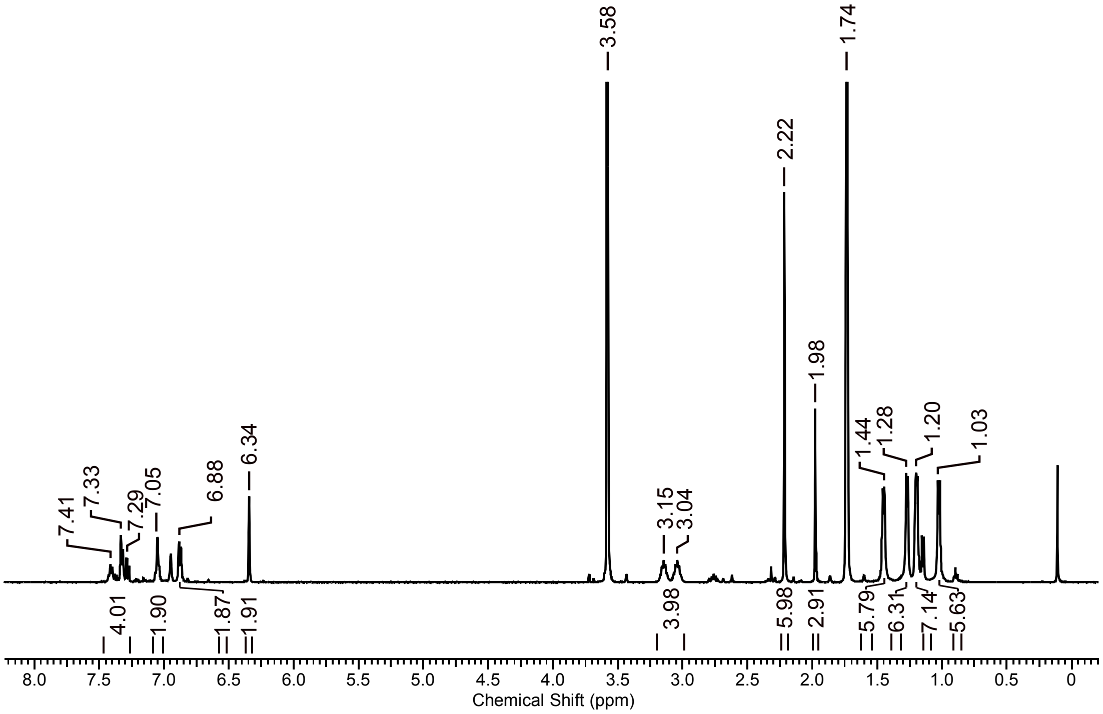

Figure S26. ^{1}H NMR spectrum of IPr·PMes (**4**) in (400 MHz, thf-d$_8$, 233 K). δ/ppm: 7.27-7.43 (4H, m, *meta*-CH, Dipp); 7.05 (2H, s, mesityl *meta*-CH); 6.88 (2H, d, imidazolylidene CH, $^3J_{HH}$ = 7.54 Hz); 6.34 (2H, s, mesityl *meta*-CH); 3.15 (1H, sept, CH, iPr, $^3J_{HH}$ = 6.28 Hz); 3.04 (1H, sept, CH, iPr, $^3J_{HH}$ = 6.59 Hz); 2.22 (6H, s, mesityl *ortho*-CH$_3$); 1.98 (3H, s, mesityl *ortho*-CH$_3$); 1.44 (6H, d, CH$_3$ of iPr, $^3J_{HH}$ = 4 Hz); 1.28 (6H, d, CH$_3$ of ^{i}Pr, $^3J_{HH}$ = 6.28 Hz); 1.20 (6H, d, CH$_3$ of iPr, $^3J_{HH}$ = 6.28 Hz); 1.03 (6H, d, CH$_3$ of iPr, $^3J_{HH}$ = 6.28 Hz).

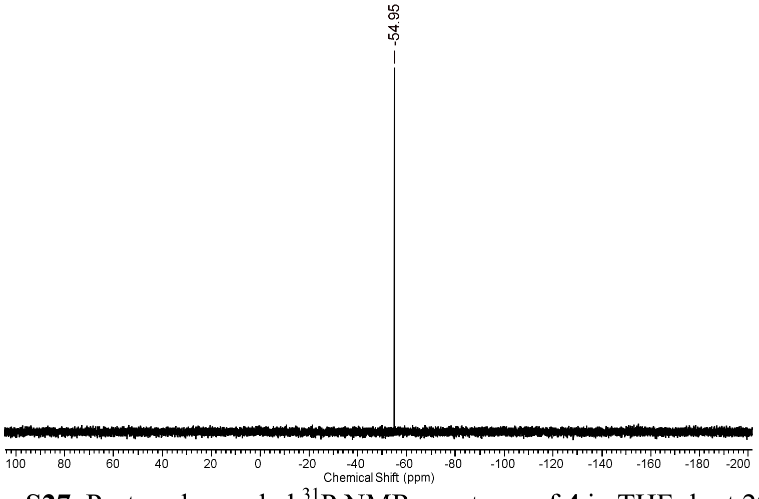

Figure S27. Proton-decoupled ^{31}P NMR spectrum of **4** in THF-d$_8$ at 298 K.

Control experiment: IMes and MesPH$_2$. A solution of IMes (0.015 g, 0.05 mmol) and MesPH$_2$ (7.6 µL, 0.05 mmol) in 1 ml benzene-d$_6$ was heated at 80°C for five days. The ^{1}H and ^{31}P NMR spectra show resonances due only to the starting materials, in a 1:1 ratio, as confirmed by comparison to the ^{1}H NMR spectra of the separate compounds.

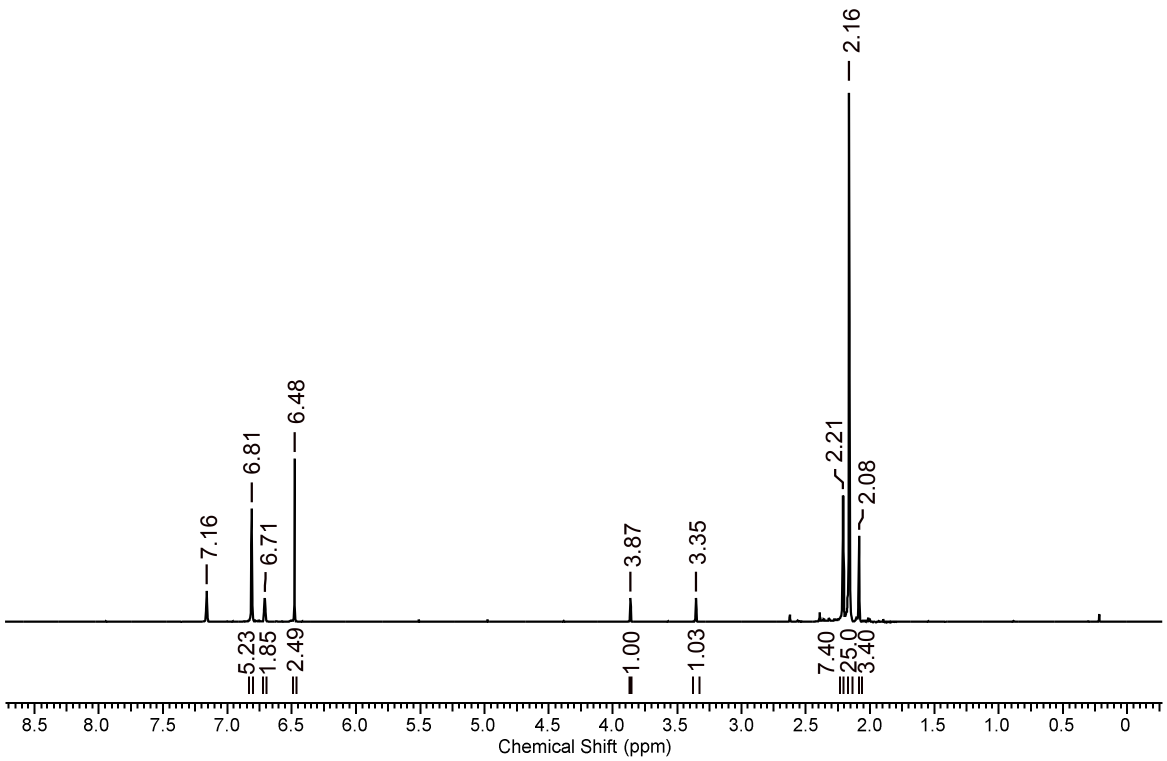

Figure S28. ^{1}H NMR spectrum of a 1:1 mixture of IMes and $MesPH_2$ after heating at 80°C for five days in benzene-d_6 (400 MHz, 298 K). δ/ppm. **$MesPH_2$:** 6.71 (2H, s, mesityl *meta*-CH); 3.61 (d, 2H, PH_2, $^{1}J_{PH}$ = 204.29 Hz); 2.21 (6H, s, mesityl *ortho*-CH_3); 2.08 (6H, s, mesityl *para*-CH_3). **IMes:** 6.81 (4H, s, mesityl *meta*-CH); 6.48 (2H, s, imidazolylidene CH); 2.16 (18H, mesityl CH_3).

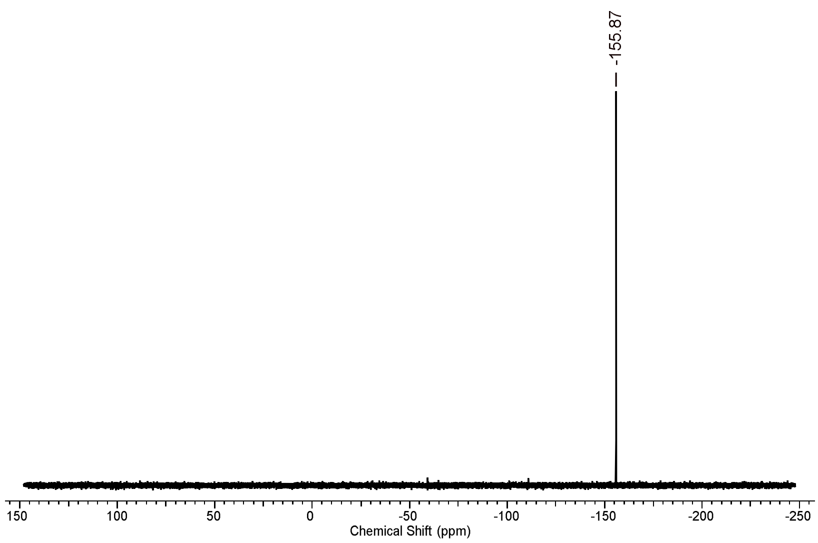

Figure S29. $^{31}P\{^{1}H\}$ NMR spectrum (298 K) of a 1:1 mixture of IMes and $MesPH_2$ after heating at 80°C for five days in benzene-d_6. δ/ppm. **$MesPH_2$:** –155.9.

Control experiment: IPr and $MesPH_2$. A solution of IPr (0.019 g, 0.05 mmol) and $MesPH_2$ (7.6 µL, 0.05 mmol) in 1 ml benzene-d_6 was heated at 80°C for five days. The ^{1}H and ^{31}P NMR spectra show resonances due only to the starting materials, in a 1:1 ratio, as confirmed by comparison to the ^{1}H NMR spectra of the separate compounds.

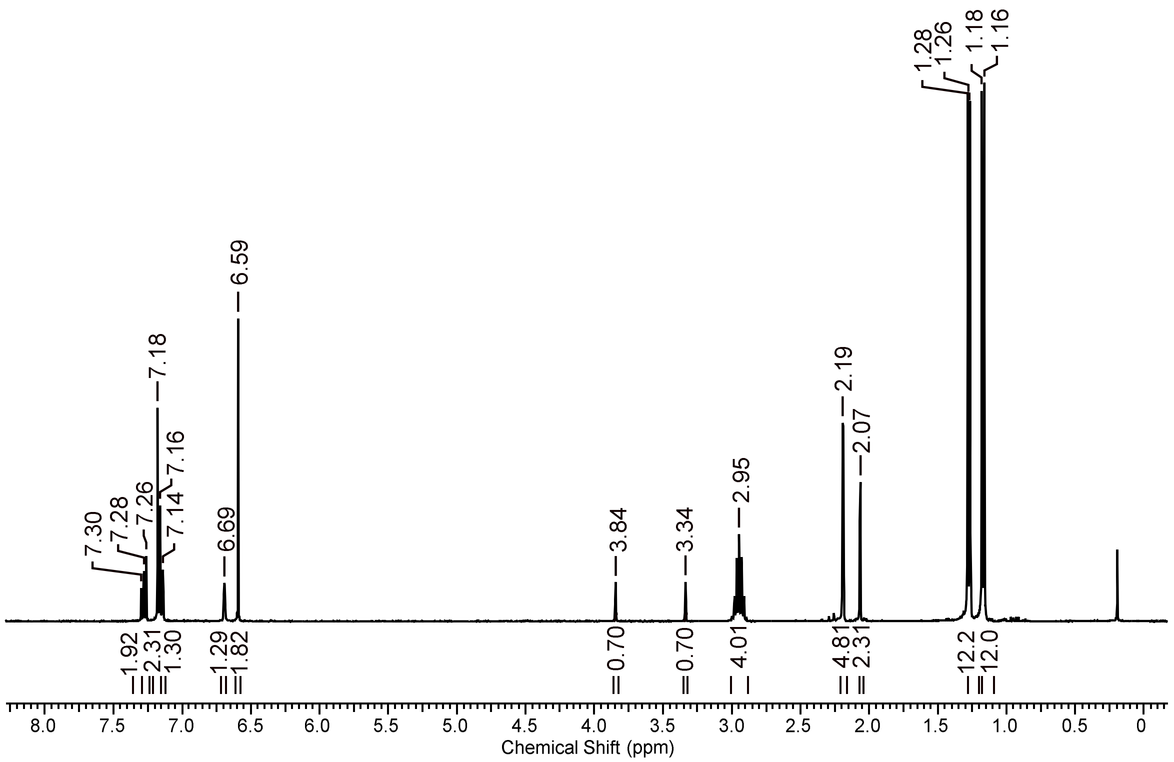

Figure S30. ^{1}H NMR spectrum of a 1:1 mixture of IPr and $MesPH_2$ after heating at 80°C for five days in benzene-d_6 (400 MHz, 298 K). δ/ppm. **$MesPH_2$:** 6.71 (2H, s, mesityl *meta*-CH); 3.61 (d, 2H, PH_2, $^{1}J_{PH}$ = 203.79 Hz); 2.20 (6H, s, mesityl *ortho*-CH_3); 2.08 (3H, s, mesityl *para*-CH_3). **IPr:** 7.28-7.32 (2H, m, aryl *para*-CH); 7.16-7.20 (4H, s, aryl *meta*-CH); 6.61 (2H, s, imidazolylidene CH); 2.97 (4H, sept, ^{i}Pr CH, $^{3}J_{HH}$ = 6.81 Hz); 1.27 (d, 12H, ^{i}Pr CH_3, $^{3}J_{HH}$ = 6.81 Hz); 1.19 (d, 12H, ^{i}Pr CH_3, $^{3}J_{HH}$ = 7.06 Hz).

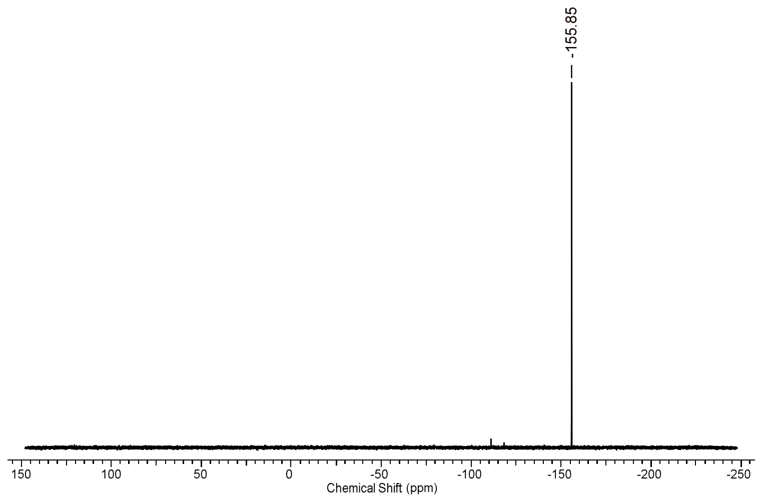

Figure S31. $^{31}P\{^{1}H\}$ NMR spectrum of a 1:1 mixture of IPr and $MesPH_2$ after heating at 80°C for five days in benzene-d_6 (298 K). δ/ppm. –155.9 ($MesPH_2$).

Control experiment: IMe_4 and $MesPH_2$. A solution of IMe_4 (0.006 g, 0.05 mmol) and $MesPH_2$ (7.6 µL, 0.05 mmol) in 1 ml benzene-d_6 was heated at 80°C for seven days. The ^{31}P NMR spectrum shows resonances due to $MesPH_2$, $IMe_4{\cdot}PMes$, and MesPHMe. The relative amounts of the three phosphorus-containing compounds were determined by integration of the mesityl meta-protons in the ^{1}H NMR spectrum at $\delta(^{1}H)$ = 7.01 ppm ($IMe_4{\cdot}PMes$), 6.76 ppm (MesPHMe) and 6.71 ppm ($MesPH_2$).

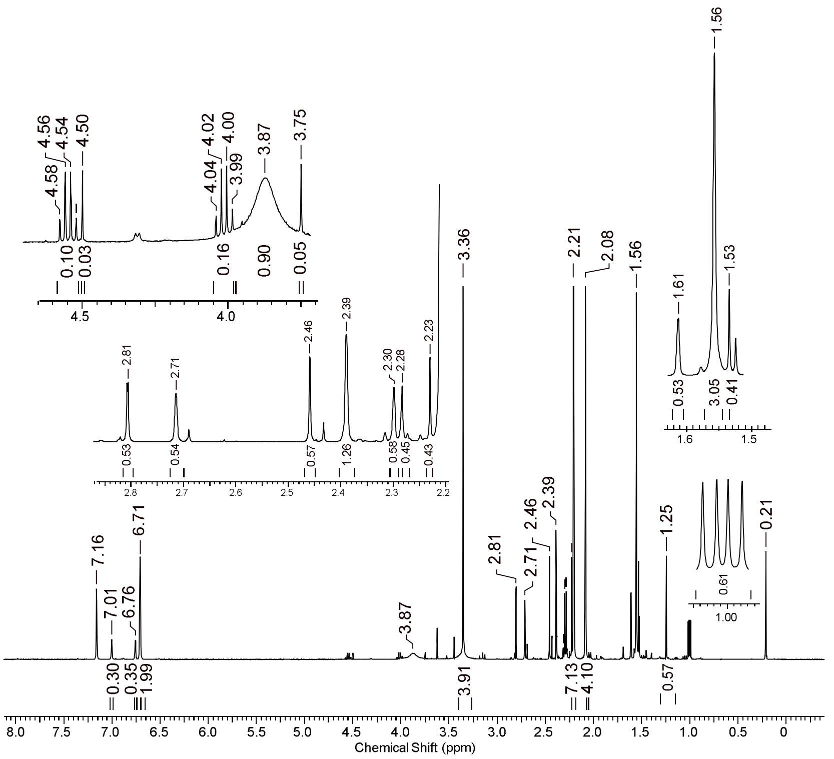

Figure S32. ^{1}H NMR spectrum of a 1:1 mixture of IMe_4 and $MesPH_2$ after heating at 80°C for seven days in benzene-d_6 (400 MHz, 298 K). δ/ppm. **$MesPH_2$**: 6.71 (2H, s, mesityl *meta*-CH); 3.87 (br, 2H, PH_2); 2.21 (6H, s, mesityl *ortho*-CH_3); 2.08 (3H, s, mesityl *para*-CH_3). **IMe_4·PMes:** 7.01 (2H, s, aryl *meta*-CH); 2.81 (6H, s, NCH_3); 2.71 (6H, s, mesityl *ortho*-CH_3); 2.28 (3H, s, mesityl *para*-CH_3); 1.25 (6H, s, CCH_3). **MesP(H)Me:** 6.76 (2H, s, aryl *meta*-CH); 4.28 (1H, dq, PH, $^{1}J_{PH}$ = 213.84 Hz; $^{3}J_{HH}$ = 7.46 Hz); 2.39 (6H, s, mesityl *ortho*-CH_3); 2.30 (3H, s, mesityl *para*-CH_3); 1.00 (3H, m, PMe). **IMe_4:** 3.36 (6H, s, NMe); 1.56 (6H, s, CMe).

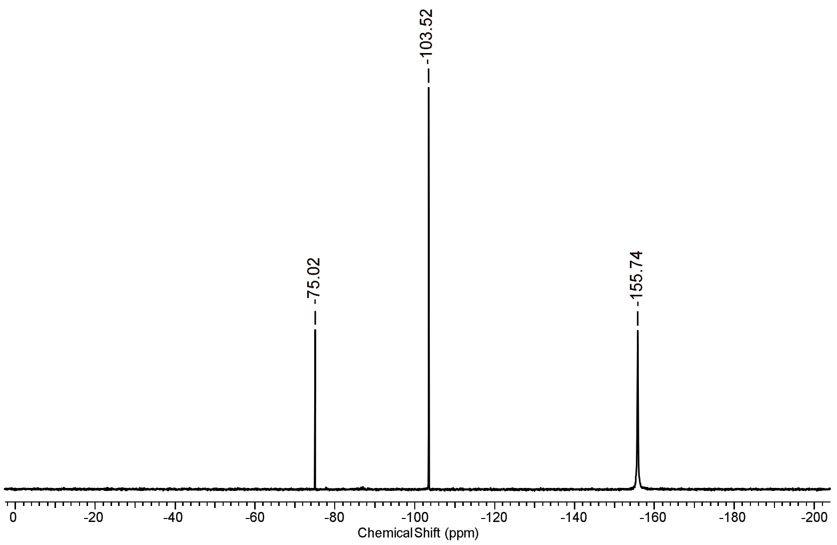

Figure S33. ^{31}P{^{1}H} NMR spectrum of a 1:1 mixture of IMe_4 and $MesPH_2$ after heating at 80°C for seven days in benzene-d_6 (298 K). δ/ppm. –75.0 (IMe_4·PMes); –103.5 (MesPHMe); –155.8 ($MesPH_2$).

Preparative-scale catalytic synthesis of IMes·PMes (3) using 10 mol % [Fe(N")$_2$]. A solution of IMes (0.15 g, 0.5 mmol) in toluene (5 ml) was added to a solution of [Fe{N(SiMe$_3$)$_2$}$_2$]$_2$ (0.019 g, 0.025 mmol) in toluene (5 ml) at room temperature and the mixture was stirred for 30 minutes. A solution of MesPH$_2$ (76 µL, 0.50 mmol) in toluene (5 ml) was added to the reaction mixture, which was stirred at 80°C for seven days. After cooling the reaction mixture, solution was filtered, concentrated to a volume of about 2-3 ml and stored at –28°C for two days. IMes·PMes was isolated as a pale yellow crystalline material (0.16 g, 71%) which was isolated by filtration, washed with cold toluene, dried *in vacuo*. The isolated material was found to be pure by ^{1}H and ^{31}P NMR spectroscopy (see Figures S22 and S23 for ^{1}H and ^{31}P NMR spectra).

NMR-scale synthesis of IMes·PMes (3) using 10 mol % [Fe(N")$_2$]. A mixture of IMes (0.015 g, 0.05 mmol), [Fe{N(SiMe$_3$)$_2$}$_2$]$_2$ (0.0019 g, 0.0025 mmol) and MesPH$_2$ (7.6 µL, 0.05 mmol) in benzene-d$_6$ (1.5 ml) was heated to 80°C in an NMR tube for seven days.

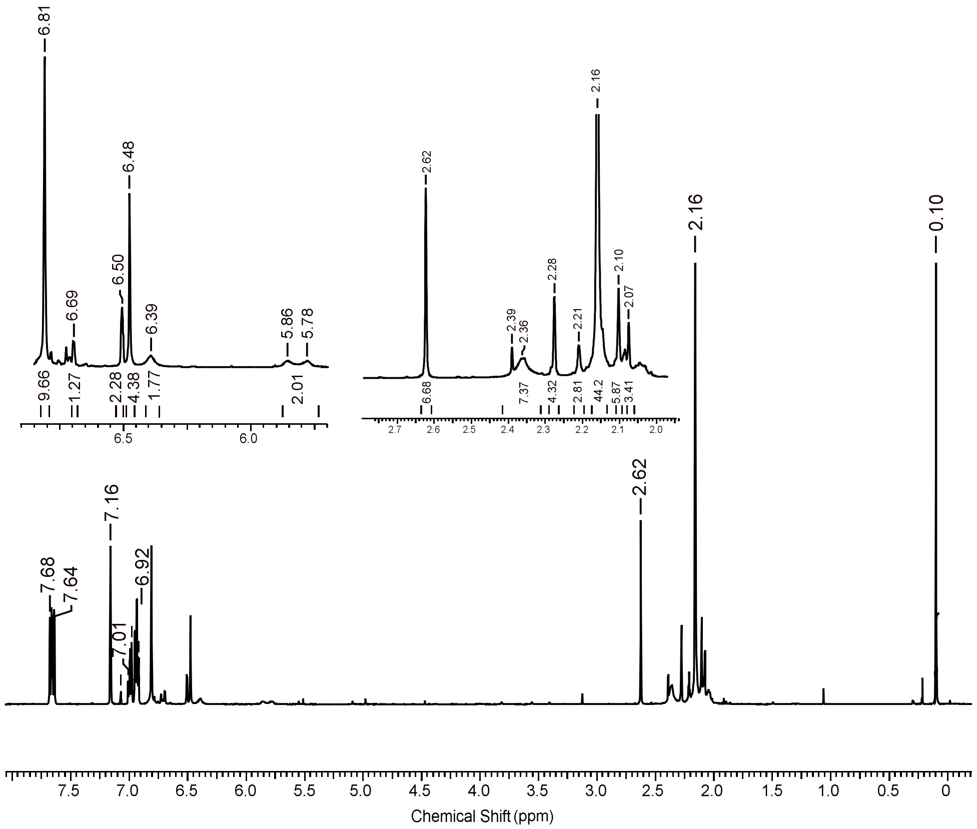

Figure S34. ^{1}H NMR spectrum of the reaction mixture for the catalytic synthesis of IMes·PMes (**3**) using 10 mol % [Fe(N")$_2$] (benzene-d$_6$, 500 MHz, 295 K). δ/ppm. **IMes·PMes**: signals broaded due to fluxionality (*cf.* Fig. S22). **MesPH$_2$:** 6.69 (s, mesityl *meta*-CH); 3.61 (d, PH$_2$, $^{1}J_{PH}$ = 202.64 Hz); 2.21 (s, mesityl *ortho*-CH$_3$); 2.07 (s, mesityl *para*-CH$_3$). **IMes:** 6.81 (s, mesityl *meta*-CH); 6.50 (2H, s, imidazolylidene CH); 2.16 (18H, mesityl CH$_3$, overlapping with CH$_3$ group of IMes·PMes).

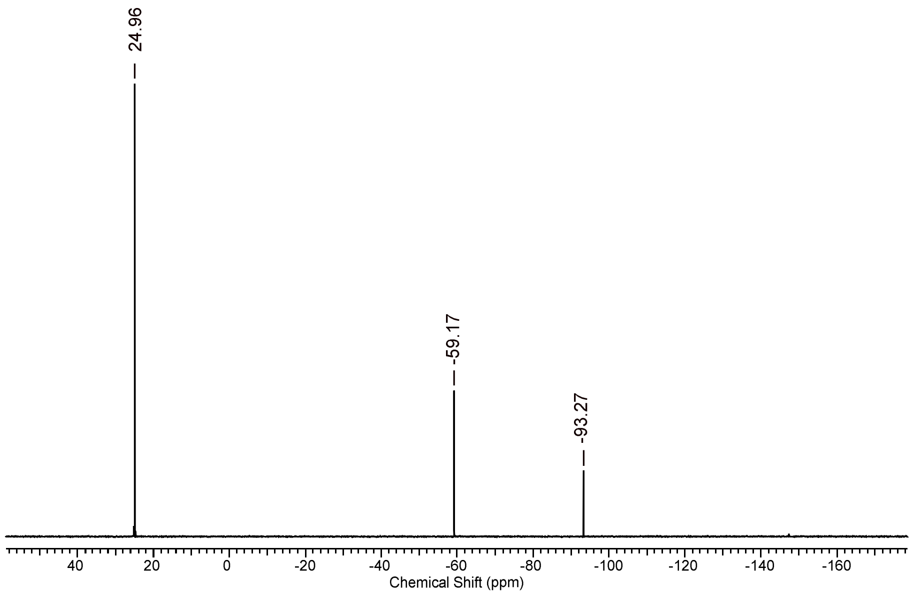

Figure S35. $^{31}P\{^{1}H\}$ NMR spectrum of the reaction mixture for the synthesis of IMes·PMes (**3**) using 10 mol % [Fe(N'')$_2$] in benzene-d$_6$ (298 K). δ/ppm –59.1 (IMes·PMes), –93.3 (Mes$_2$PH). δ = +25.0 ppm corresponds to a benzene solution of Ph$_3$P=O contained in a capillary.

Attempted preparative-scale catalytic synthesis of IPr·PMes (4) using 10 mol % [Fe(N'')$_2$]. A solution of IPr (0.19 g, 0.5 mmol) in toluene (5 ml) was added to a solution of [Fe{N(SiMe$_3$)$_2$}$_2$]$_2$ (0.019 g, 0.025 mmol) in toluene (5 ml) at room temperature and the mixture was stirred for 30 minutes. A solution of MesPH$_2$ (76 μL, 0.50 mmol) in toluene (5 ml) was added to the reaction mixture, which was stirred at 80°C for seven days. After cooling the reaction mixture, the solution was filtered, concentrated to a volume of about 3 ml and stored at –28°C for two days. The resulting pale yellow crystals were isolated by filtration, washed with cold toluene, dried *in vacuo*. Several crystals were analysed by X-ray crystallography, which revealed that the reaction had produced a mixture of IPr, IPr·PMes and [(*a*IPr)Fe{N(SiMe$_3$)$_2$}$_2$].

Attempted NMR-scale synthesis of IPr·PMes (4) using 10 mol % [Fe(N'')$_2$]. A mixture of IPr (0.019 g, 0.05 mmol), [Fe{N(SiMe$_3$)$_2$}$_2$]$_2$ (0.0019 g, 0.0025 mmol) and MesPH$_2$ (7.6 μL, 0.05 mmol) in benzene-d$_6$ (1.5 ml) was heated to 80°C in an NMR tube for seven days.

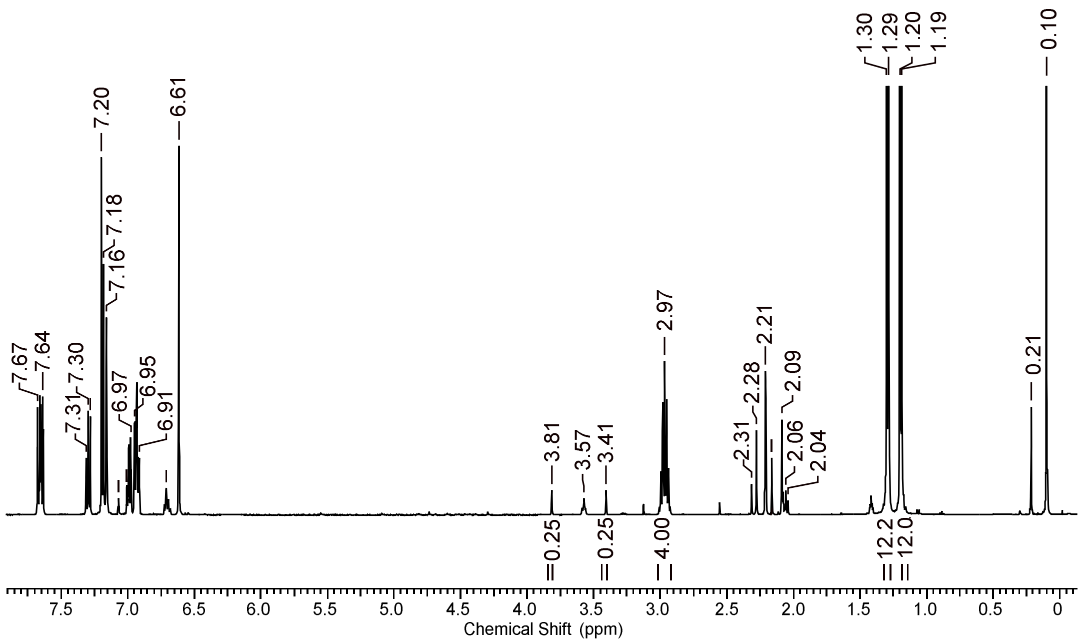

Figure S36. ^{1}H NMR spectrum of the reaction mixture for the catalytic synthesis of **IPr·PMes** (**4**) using 10 mol % [Fe(N")$_2$] (benzene-d$_6$, 500 MHz). δ/ppm. **IPr·PMes**: 1.45 (s, br, 12H, IPr CH$_3$); 1.09 (s, br, 12H, IPr CH$_3$); 2.06 (s, 3H, mesityl *para*-CH$_3$); 2.54 (s, 6H, mesityl *ortho*-CH$_3$); 3.28 (sept, 4H, $^{3}J_{HH}$ = 6.1 Hz, IPr methine CH); 6.54 (2H, s, IMes backbone); **MesPH$_2$:** 6.71 (2H, s, mesityl *meta*-CH); 3.60 (d, 2H, PH$_2$, $^{1}J_{PH}$ = 203 Hz); 2.21 (6H, s, mesityl *ortho*-CH$_3$); 2.09 (3H, s, mesityl *para*-CH$_3$). **IPr:** 7.28-7.32 (2H, m, aryl *para*-CH); 7.16-7.20 (4H, s, aryl *meta*-CH); 6.61 (2H, s, imidazolylidene CH); 2.97 (4H, sept, ^{i}Pr CH, $^{3}J_{HH}$ = 7 Hz); 1.295 (d, 12H, ^{i}Pr CH$_3$, $^{3}J_{HH}$ = 7 Hz); 1.195 (d, 12H, ^{i}Pr CH$_3$, $^{3}J_{HH}$ = 7 Hz). HN(SiMe$_3$)$_2$: 0.10 (s, 36H).

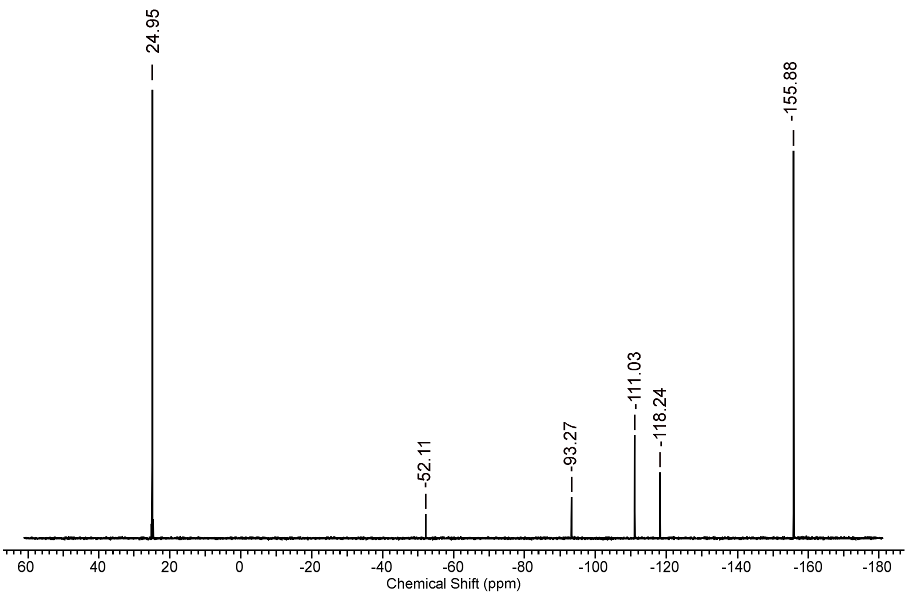

Figure S37. ^{31}P{^{1}H} NMR spectrum of the reaction mixture for the catalytic synthesis of IPr·PMes (**4**) using 10 mol % [Fe(N")$_2$] (298 K). δ/ppm. –52.1 (IPr·PMes); –93.3 (Mes$_2$PH); –111.0 (*D*-/*L*-MesP(H)P(H)Mes); –118.2 (*meso*-MesP(H)P(H)Mes); –155.9 (MesPH$_2$). δ = +25.0 ppm corresponds to a benzene solution of Ph$_3$P=O contained in a capillary.

Preparative-scale catalytic synthesis of IMe$_4$·PMes (5) using 10 mol % [Fe(N")$_2$]. A solution of IMe$_4$ (0.06 g, 0.5 mmol) in toluene (5 ml) was added to a solution of [Fe{N(SiMe$_3$)$_2$}$_2$]$_2$ (0.019 g, 0.025 mmol) in toluene (5 ml) at room temperature, and the mixture was stirred for 30 minutes. A solution of MesPH$_2$ (76

μL, 0.5 mmol) in toluene (5 ml) was added to the reaction mixture, which was stirred at 80°C for seven days. After cooling, the reaction mixture was filtered, reduced to a volume to about 6 ml, and stored at –28°C overnight, which produced **2-Fe**·toluene (0.02 g, 0.02 mmol). The crystals of **2-Fe**·toluene were separated by filtration. Removal of all solvent from the filtrate gave a red-yellow waxy solid, which was washed with cold hexane (5 ml) to remove unreacted $MesPH_2$. The solid obtained after washing was re-dissolved in toluene (10 ml), filtered, reduced in volume to about 1-2 ml, and stored at –28°C for two days. The resulting pale yellow crystals were isolated by filtration, washed with cold hexane, dried *in vacuo*. The isolated material (0.092 g) was found to be mixture of IMe_4 and IMe_4·PMes and $(MesPH)_2$ by ^1H and ^{31}P NMR spectroscopy and X-ray crystallography.

NMR-scale synthesis of IMe_4·PMes (5) using 10 mol % [Fe(N")$_2$]. A mixture of IMe_4 (0.015 g, 0.05 mmol), $[Fe\{N(SiMe_3)_2\}_2]_2$ (0.0019 g, 0.0025 mmol) and $MesPH_2$ (7.6 μL, 0.05 mmol) in benzene-d_6 (1.5 ml) was heated to 80°C in an NMR tube for seven days.

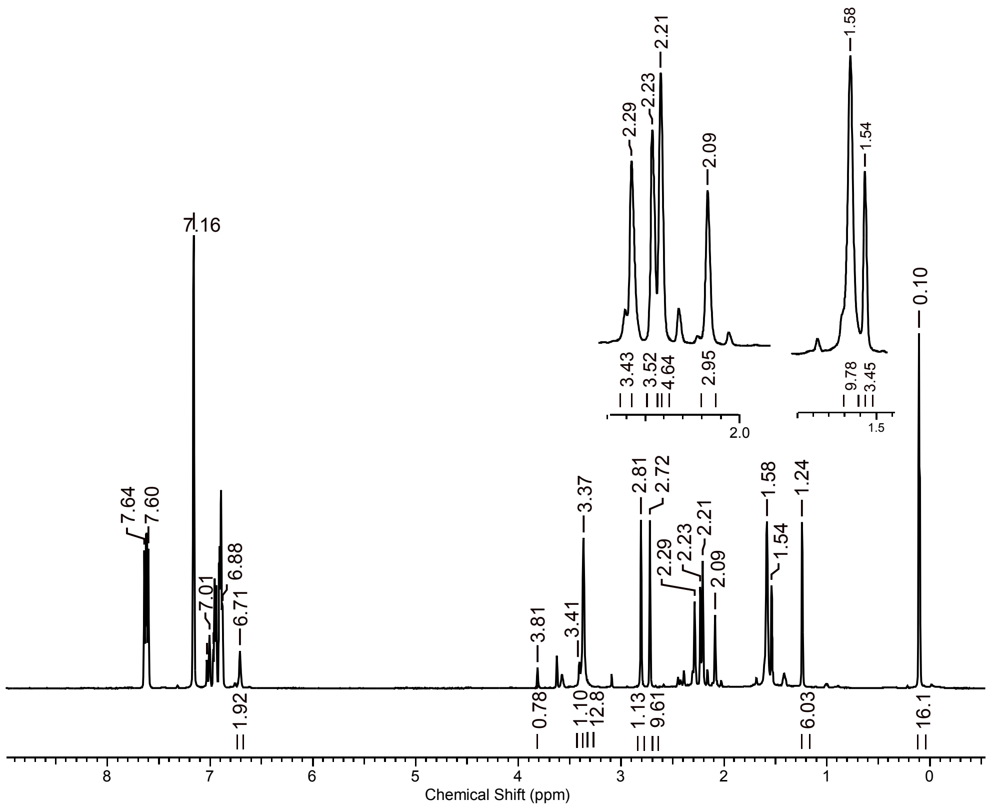

Figure S38. ^1H NMR spectrum of the reaction mixture for the catalytic synthesis of IMe_4·PMes (**5**) using 10 mol % [Fe(N")$_2$] (benzene-d_6, 500 MHz). δ/ppm. **IMe_4·PMes**: 7.01 (2H, s, mesityl *meta*-CH); 2.81 (6H, s, NCH_3); 2.72 (6H, s, mesityl *ortho*-CH_3); 2.29 (3H, s, mesityl *para*-CH_3); 1.24 (6H, s, CCH_3). **$MesPH_2$**: 6.71 (s, 2H, mesityl *meta*-CH); 3.60 (d, 1H, $^1J_{PH}$ = 202.84 Hz); 2.21(6H, mesityl *ortho*-CH_3); 2.09 (3H, mesityl *para*-CH_3). **IMe_4**: 3.37 (s, 6H, N-Me); 1.57 (6H, Me). **$HN(SiMe_3)_2$**: 0.10 (s, 36H, $SiMe_3$).

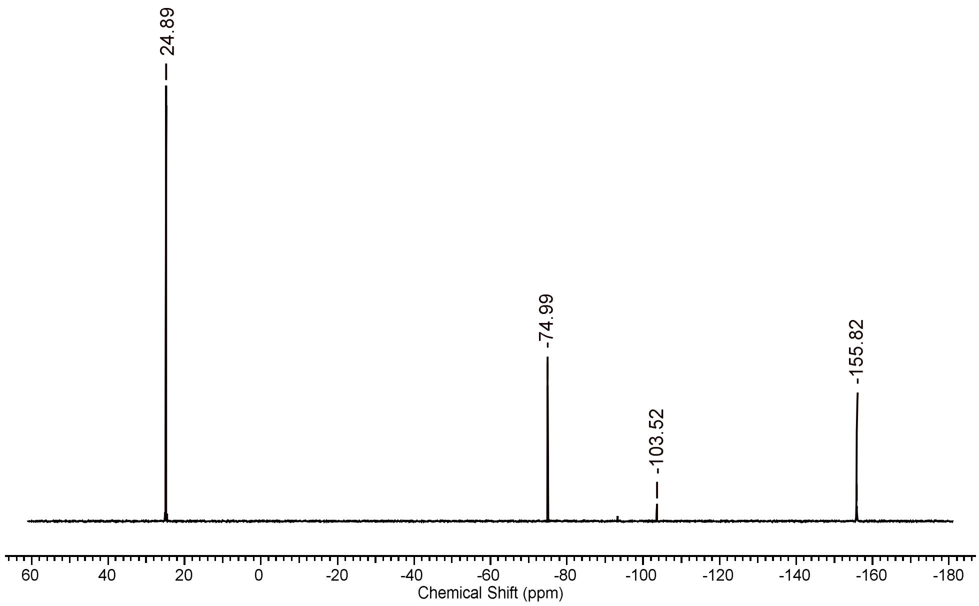

Figure S39. $^{31}P\{^1H\}$ NMR spectrum of the reaction mixture for the catalytic synthesis of **IMe_4·PMes** (**5**) using 10 mol % **1-Fe**. δ/ppm: –75.0 (IMe_4·PMes); –103.5 (s, Mes_2PH); –155.8 ($MesPH_2$). δ = +24.9 ppm corresponds to a benzene solution of $Ph_3P=O$ contained in a capillary.

Preparative-scale catalytic synthesis of IMes·PPh (6) using 10 mol % [Fe(N")$_2$]. A solution of IMes (0.15 g, 0.5 mmol) in toluene (10 ml) was added to a solution of $[Fe\{N(SiMe_3)_2\}_2]_2$ (0.019 g, 0.025 mmol) in toluene (5 ml) at room temperature and the mixture was stirred for 30 minutes. A solution of $PhPH_2$ (0.7 ml, 10% w/w hexane solution, 0.5 mmol) was added to the reaction mixture, which was stirred at 80°C for two days. The yellow-red solution was evaporated to dryness and the resulting solid was treated with an excess of LiCl as a THF solution. The mixture was again evaporate to dryness, extracted with toluene (10 ml), filtered, reduced in volume to about 2-3 ml, and stored at –28°C overnight. The resulting material was washed with hexane, and IMes·PPh was isolated as pale yellow crystals (0.11 g, 46%). Purity was established by comparison of the ^1H and ^{31}P NMR spectroscopy to those of the previously reported compound.[6]

NMR-scale catalytic synthesis of IMes·PPh (6) using 10 mol % [Fe(N'')$_2$]. A mixture of IMes (0.015 g, 0.05 mmol), $[Fe\{N(SiMe_3)_2\}_2]_2$ (0.0019 g, 0.0025 mmol) and $PhPH_2$ (0.07ml of 10% w/w hexane solution, 0.05 mmol) in benzene-d_6 (1.5 ml) was heated to 80°C in an NMR tube for two days.

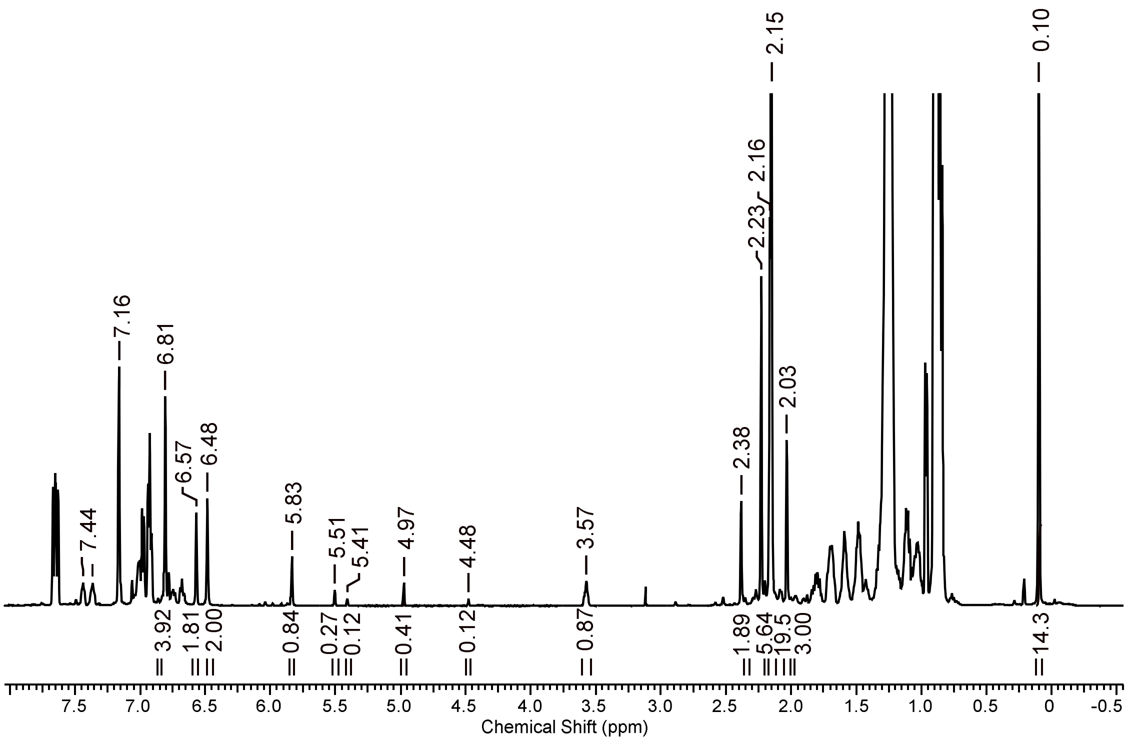

Figure S40. ^{1}H NMR spectrum of the reaction mixture for the catalytic synthesis of IMes·PPh (**6**) using 10 mol % [Fe(**N''**)$_2$] (400 MHz, benzene-d$_6$, 298 K). δ/ppm. **IMes**: 6.81 (2H, s, mesityl *meta*-CH); 6.48 (2H, imidazolylidene CH); 2.16 (18H, s, mesityl CH$_3$). **IMes·PPh**: 7.42-7.44 (m, 2H, PPh); 6.66-6.78 (3H, m, PPh); (4H, s, mesityl *meta*-CH); 5.83 (2H, s, imidazolylidene); 2.23 (12H, s, mesityl *ortho*-CH$_3$); 2.03 (6H, s, mesityl *para*-CH$_3$). **IMesH$_2$**: 6.78 (4H, s, mesityl *meta*-CH); 5.51 (2H, s, NCH$_2$N); 4.97 (2H, imidazolylidene CH); 2.83 (18H, s, mesityl CH$_3$). **Ph$_2$PH**: 7.39-7.35 (2H, m, phenyl *meta*-CH); 7.02-7.00 (2H, m, phenyl *ortho/para*-CH); 3.57 (2H, m, PH). **HN(SiMe$_3$)$_2$:** 0.10 (18H, s).

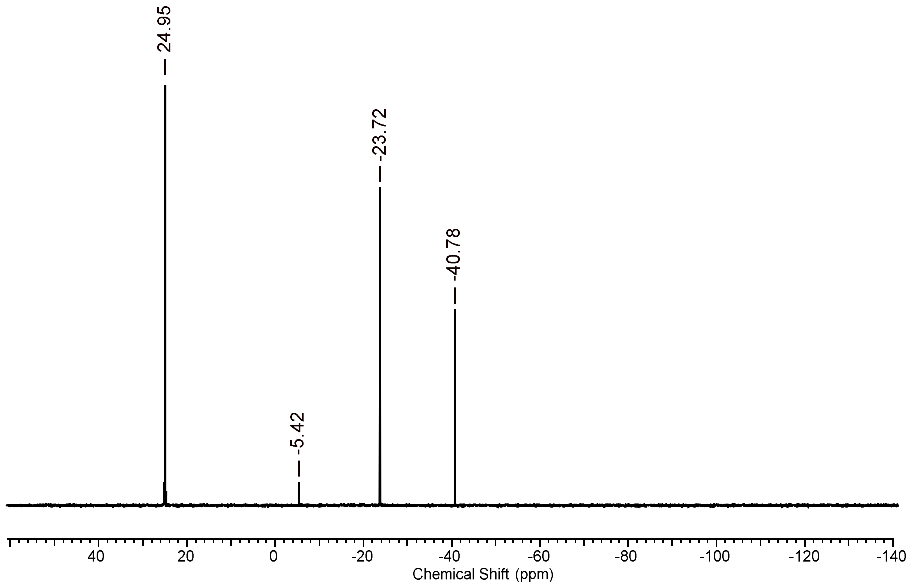

Figure S41. ^{31}P{^{1}H} NMR spectrum of the reaction mixture for the catalytic synthesis of IMes·PPh (**6**) using 10 mol % [Fe(N'')$_2$] (298 K). δ/ppm: –5.42 (P$_5$Ph$_5$); –23.7 (IMes·PPh); –40.8 (Ph$_2$PH). δ = +25.0 ppm corresponds to a benzene solution of Ph$_3$P=O contained in a capillary.

Preparative-scale catalytic synthesis of IPr·PPh (7) using 10 mol % [Fe(N'')$_2$]. A solution of IPr (0.19 g, 0.5 mmol) in toluene (10 ml) was added to a solution of [Fe{N(SiMe$_3$)$_2$}$_2$]$_2$ (0.0019 g, 0.0025 mmol) in toluene (5 ml) at room temperature and the mixture was stirred for 30 minutes. A solution of PhPH$_2$ (0.7ml of 10% w/w hexane solution, 0.5 mmol) was added to the reaction mixture, which was stirred at 80°C for seven days. The resulting yellow-red solution was filtered, reduced in volume to about 2-3 ml and stored at –28°C overnight. Unreacted IPr (0.055 g) was crystallized from the reaction (characterized by ^{1}H NMR spectroscopy). The nascent solution was filtered and evaporated to dryness, which gave a yellow waxy solid, which was found to consist of IPr·PPh and unreacted PhPH$_2$.

NMR-scale catalytic synthesis of IPr·PPh (7) using 10 mol % [Fe(N'')$_2$]. A mixture of IPr (0.019 g, 0.05 mmol), [Fe{N(SiMe$_3$)$_2$}$_2$]$_2$ (0.0019 g, 0.0025 mmol) and PhPH$_2$ (0.07 ml of 10% w/w hexane solution, 0.05 mmol) in benzene-d$_6$ (1.5 ml) was heated to 80°C in an NMR tube for 5 days. Based on the integration of the isopropyl methine environments in the ^{1}H NMR spectrum, the reaction mixture consists of 22.6 % unreacted IPr (22%), IPr·PPh (30%) and IPrH$_2$ (48%).

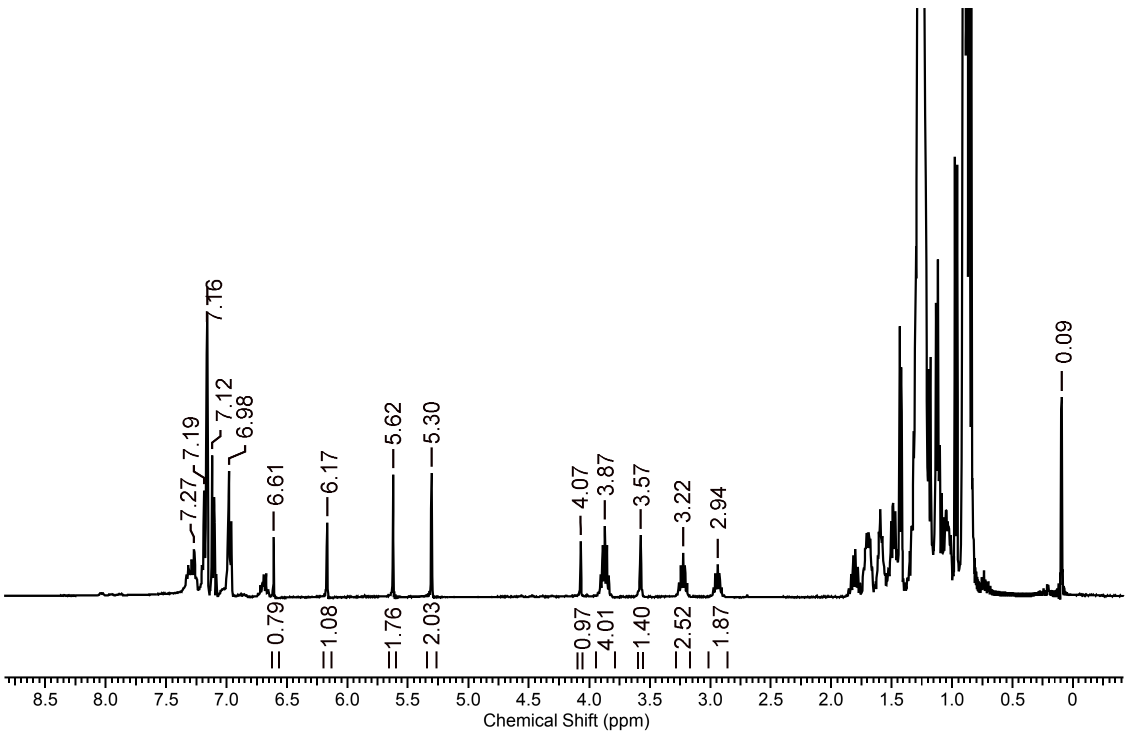

Figure S42. ^{1}H NMR spectrum of the reaction mixture for the catalytic synthesis of IPr·PPh (**7**) using 10 mol % [Fe(**N''**)$_2$] (500 MHz, benzene-d$_6$, 298 K). δ/ppm. **PhPH$_2$**: 7.25-7.29 (2H, m, PPh); 6.98-7.00 (3H, m, PPh); 3.83 (2H, d, PH$_2$, $^1J_{PH}$ = 198 Hz); 2.0-0.7 (hexane). **IPr**: 7.28-7.32 (2H, m, ArH); 7.16-7.20 (4H, m, ArH); 6.61 (2H s, imidazolylidene CH); 2.94 (4H, sept, ^{i}Pr CH, $^3J_{HH}$ = 8 Hz); 1.29 (12H, d, ^{i}Pr CH$_3$, $^3J_{HH}$ = 8 Hz); 1.19 (12H, d, ^{i}Pr CH$_3$, $^3J_{HH}$ = 8 Hz). **IPr·PPh**: 7.26-7.40 (2H, m, ArH); 7.10 (2H, t, $^3J_{HH}$ = 7.6 Hz); 6.98 (4H, d, $^3J_{HH}$ = 7.6 Hz); 6.66-6.68 (3H, m, ArH); 6.19 (2H s, imidazolylidene CH); 3.22 (4H, sept, ^{i}Pr CH, $^3J_{HH}$ = 6.7 Hz); 1.43 (12H, d, ^{i}Pr CH$_3$, $^3J_{HH}$ = 6.7 Hz); 1.12 (12H, d, ^{i}Pr CH$_3$, $^3J_{HH}$ = 6.7 Hz). **IPrH$_2$.** 7.09-7.12 (2H, m, ArH); 6.67-6.71 (4H, m, ArH); 5.62 (2H, s, imidazolylidene CH); 5.30 (2H, s, NCH$_2$N); 3.87 (4H, sept, ^{i}Pr CH, $^3J_{HH}$ = 4 Hz); 1.26 (12H, d, ^{i}Pr CH$_3$, $^3J_{HH}$ = 8 Hz); 0.96 (12H, d, ^{i}Pr CH$_3$, $^3J_{HH}$ = 8 Hz, overlapping with hexane).

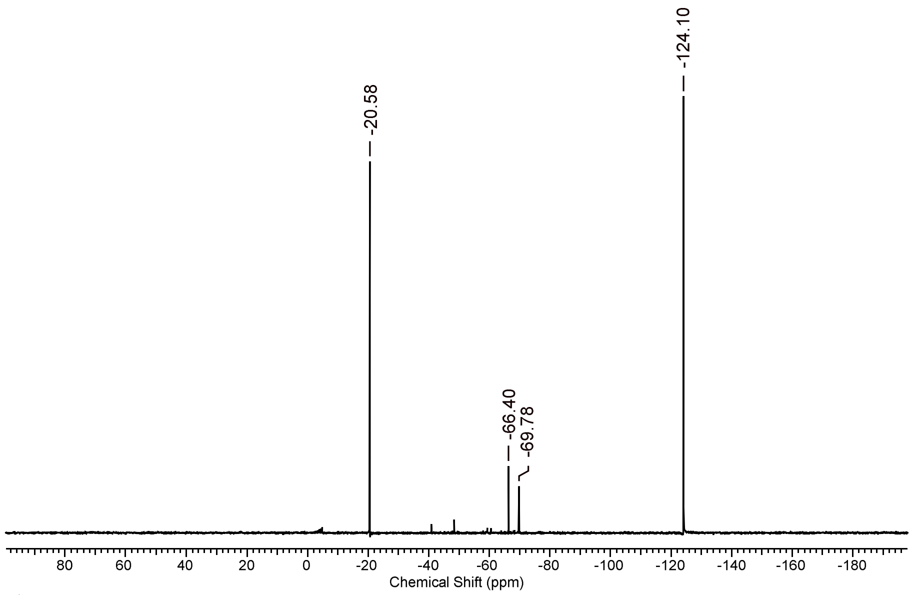

Figure S43. $^{31}P\{^{1}H\}$ NMR spectrum (benzene-d_6, 298 K) of the reaction mixture for the catalytic synthesis of IPr·PPh (**7**) using 10 mol % [Fe(N'')$_2$] (298 K). δ/ppm: –20.6 (IPr·PPh); –66.4, –69.8 (PhPHPHPh); –124.0 ($PhPH_2$).

Preparative-scale catalytic synthesis of IMe_4·PPh (11) using 10 mol % [Fe(N'')$_2$]. A solution of IMe_4 (0.06 g, 0.5 mmol) in toluene (10 ml) was added to a solution of $[Fe\{N(SiMe_3)_2\}_2]_2$ (0.0019 g, 0.0025 mmol) in toluene (5 ml) at room temperature and the mixture was stirred for 30 minutes. A solution of $PhPH_2$ (0.7 ml of 10% w/w hexane solution, 0.5 mmol) was added to the reaction mixture, which was stirred at 80°C for seven days. The resulting yellow-red solution was evaporated to dryness and the resulting solid was treated with an excess of LiCl as a THF solution. The solution was again evaporate to dryness, extracted with toluene (10 ml), filtered, reduced in volume to about 2-3 ml and stored –28°C overnight. The resulting yellow crystalline material was washed with cold hexane and dried in vacuo, allowing **8** to be isolated as pale-yellow crystals (0.048 g, 41%). The isolated material was found to be pure by ^{1}H and ^{31}P NMR spectroscopy, with the chemical shifts due to **8** being identical to those previously reported.[6]

NMR-scale catalytic synthesis of IMe_4·PPh (8) using 10 mol % [Fe(N'')$_2$]. A mixture of IMe_4 (0.006 g, 0.05 mmol), $[Fe\{N(SiMe_3)_2\}_2]_2$ (0.0019 g, 0.0025 mmol) and $PhPH_2$ (0.07 ml of 10% w/w hexane solution, 0.05 mmol) in benzene-d_6 (1.5 ml) was heated to 80°C in an NMR tube for two days. Based on integration of the NMe ^{1}H integrations (Figure S39), IMe_4·PPh and IMe_4H_2 form in an approximate 2:1 ratio.

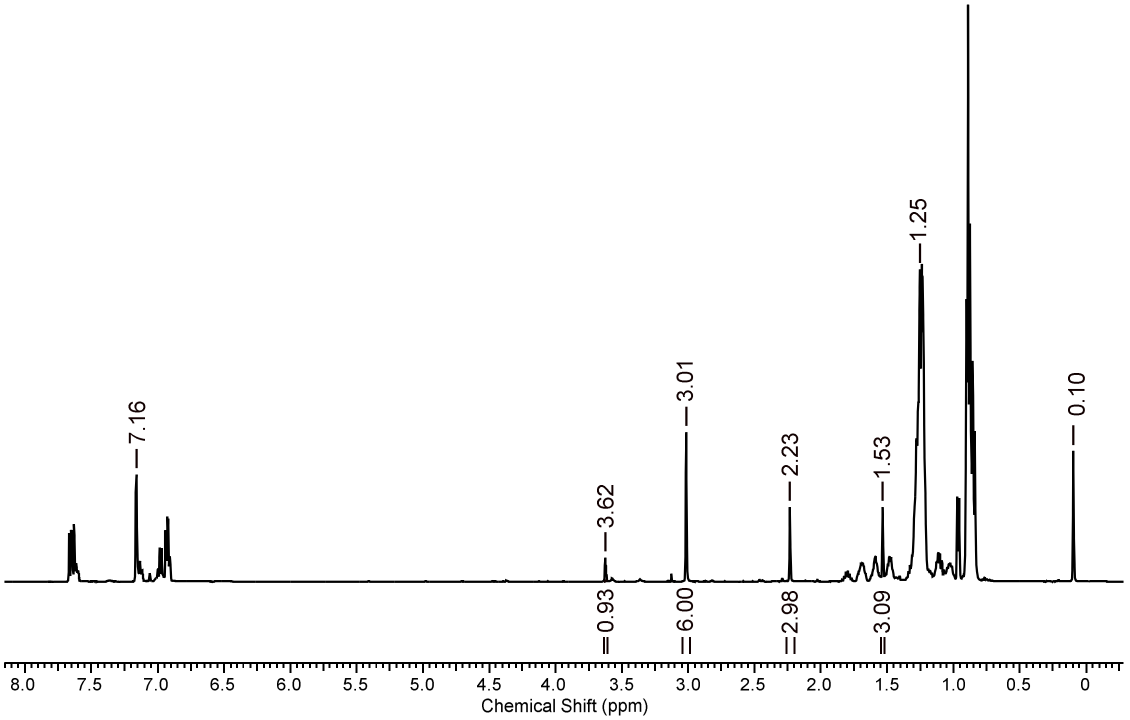

Figure S44. ^{1}H NMR spectrum of the reaction mixture for the catalytic synthesis of IMe_4·PPh (**8**) using 10 mol % [Fe(**N''**)$_2$] (500 MHz, benzene-d_6, 298 K). δ/ppm. **IMe_4·PPh**: 6.9-7.3 (5H, m, PPh); 2.23 (6H, s, NMe); 1.25 (6H, s, CCMe). **IMe_4H_2**: 3.62 (2H, s, NCH_2N); 2.23 (6H, s, NMe); 1.53 (6H, s, CMe). Unlabelled peaks between 6.50-7.75 ppm are the Ph_3PO internal standard.

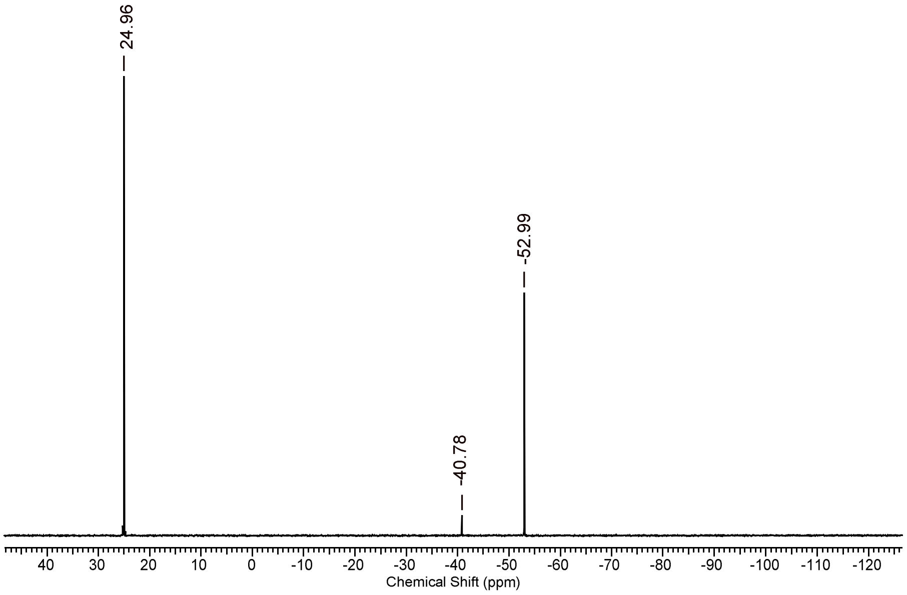

Figure S45. $^{31}P\{^{1}H\}$ NMR spectrum of the reaction mixture for the catalytic synthesis of IMe_4·PPh (**8**) using 10 mol % [Fe(N'')$_2$] (benzene-d_6, 298 K). δ/ppm: –40.8 (Ph_2PH); –53.0 (IMe_4·PPh). δ = +25.0 ppm corresponds to a benzene solution of Ph_3P=O contained in a capillary.

Control experiment: IMes and $PhPH_2$. A solution of IMes (0.015 g, 0.05 mmol) and $PhPH_2$ (0.07 ml of 10% w/w hexane solution, 0.05 mmol) in 1 ml benzene-d_6 was heated at 80°C for three days. A 1:1 ratio of IMes·PPh and $IMesH_2$ was determined by integration of the ^{1}H resonances corresponding to the imidazolylidene backbone environment.

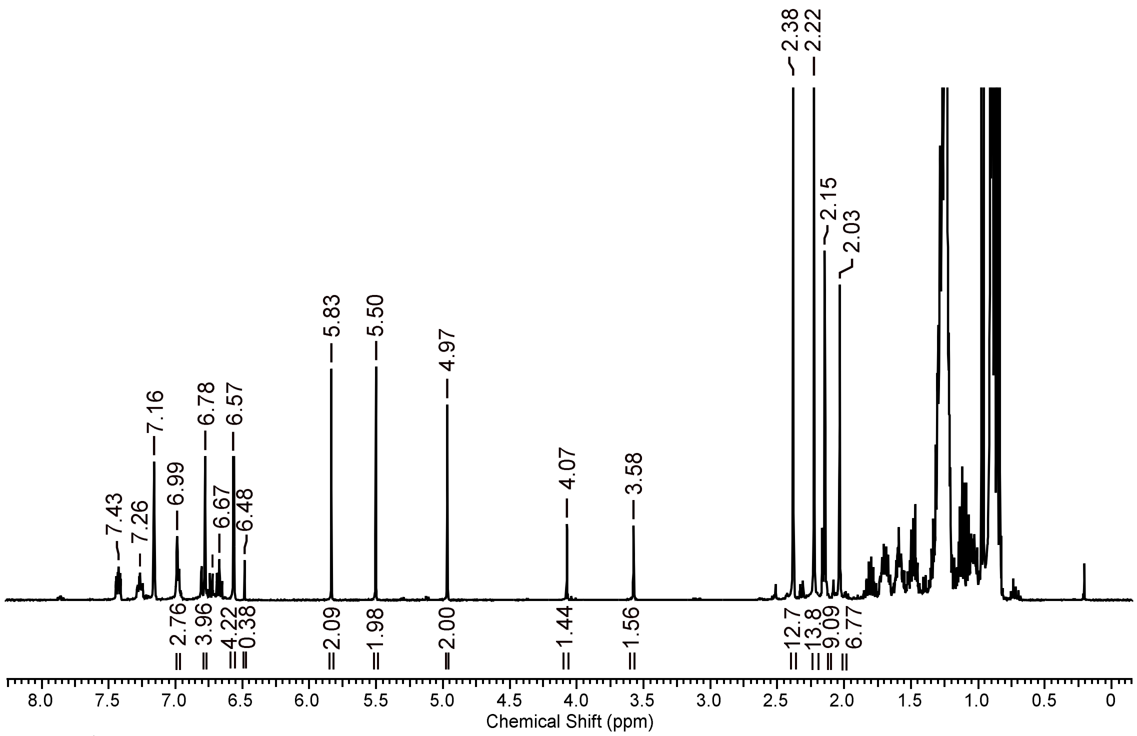

Figure S46. ^{1}H NMR spectrum of the IMes/$PhPH_2$ control experiment (400 MHz, benzene-d_6, 298 K). δ/ppm. **$PhPH_2$**: 7.25-7.29 (2H, m, PPh); 6.98-7.00 (3H, m, PPh); 3.83 (2H, d, PH_2, $^{1}J_{PH}$ = 198.74 Hz); 2.0-0.7 (hexane). **IMes**: 6.78 (2H, s, mesityl *meta*-CH); 6.48 (2H, imidazolylidene CH); 2.15 (18H, s, mesityl CH_3). **IMes·PPh**: 7.42-7.44 (m, 2H, PPh); 6.66-6.77 (3H, m, PPh); (4H, s, mesityl *meta*-CH); 5.83 (2H, s, imidazolylidene); 2.22 (12H, s, mesityl *ortho*-CH_3); 2.03 (6H, s, mesityl *para*-CH_3). **$IMesH_2$**: 6.78 (4H, s, mesityl *meta*-CH); 5.50 (2H, s, NCH_2N); 4.97 (2H, imidazolylidene CH); 2.83 (18H, s, mesityl CH_3). **Hexane:** 0.5-1.8.

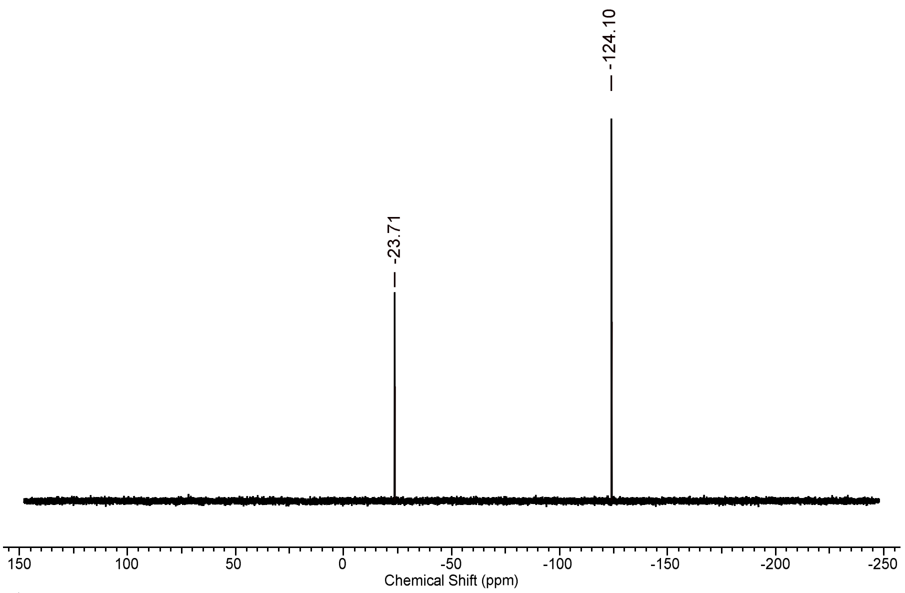

Figure S47. $^{31}P\{^{1}H\}$ NMR spectrum of the IMes/$PhPH_2$ control experiment (benzene-d_6, 298 K). δ/ppm: –23.7 (IMes·PPh); –124.1 ($PhPH_2$).

Control experiment: IPr and $PhPH_2$. A solution of **IPr** (0.019 g, 0.05 mmol) and **$PhPH_2$** (0.07 ml of 10% w/w hexane solution, 0.05 mmol) in 1 ml benzene-d_6 was heated at 80°C for five days. A 5:1:1 ratio of IPr, IPr·PPh and $IPrH_2$ was determined by integration of the ^{1}H resonances corresponding to the imidazolylidene backbone environment.

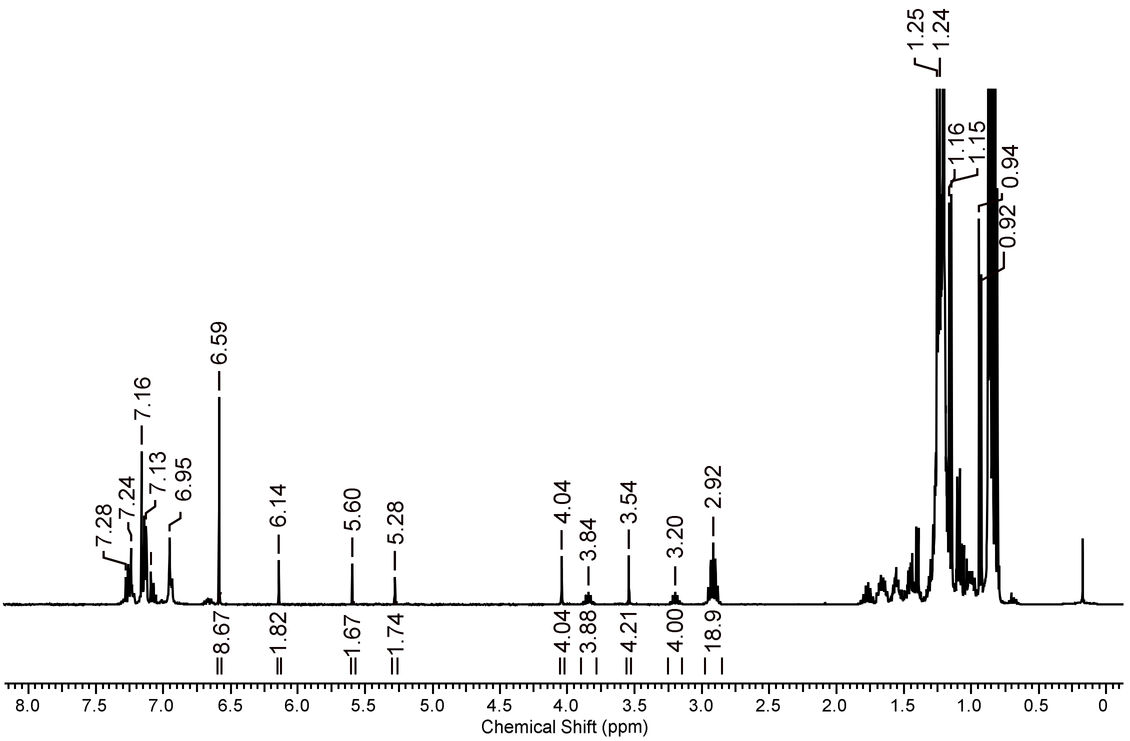

Figure S48. ^{1}H NMR spectrum of the IPr/$PhPH_2$ control experiment (400 MHz, benzene-d_6, 298 K). δ/ppm. **$PhPH_2$**: 7.24-7.28 (2H, m, PPh); 6.98-7.00 (3H, m, PPh); 3.84 (2H, d, PH_2, $^{1}J_{PH}$ = 198.74 Hz); 2.0-0.7 (hexane). **IPr**: 7.28-7.32 (2H, m, ArH); 7.13-7.18 (4H, m, ArH); 6.59 (2H s, imidazolylidene CH); 2.92 (4H, sept, ^{i}Pr CH, $^{3}J_{HH}$ = 6.81 Hz); 1.245 (12H, d, ^{i}Pr CH_3, $^{3}J_{HH}$ = 6.81 Hz); 1.155 (12H, d, ^{i}Pr CH_3, $^{3}J_{HH}$ = 7.06 Hz). **IPr·PPh**: 7.26-7.30 (2H, m, ArH); 7.13 (2H, m, ArH); 6.95 (4H, d, $^{3}J_{HH}$ = 7.82 Hz); 6.66-6.68 (3H, m, ArH); 6.14 (2H s, imidazolylidene CH); 3.20 (4H, sept, ^{i}Pr CH, $^{3}J_{HH}$ = 6.81 Hz); 1.43 (12H, d, ^{i}Pr CH_3, $^{3}J_{HH}$ = 6.56 Hz, overlapping with hexane); 1.12 (12H, d, ^{i}Pr CH_3, $^{3}J_{HH}$ = 6.56 Hz). **$IPrH_2$:** 7.09-7.12 (2H, m, ArH); 6.67-6.71 (4H, m, ArH); 5.60 (2H, s, imidazolylidene CH); 5.28 (2H, s, NCH_2N); 3.84 (4H, sept, ^{i}Pr CH, $^{3}J_{HH}$ = 6.81 Hz); 1.26 (12H, d, ^{i}Pr CH_3, $^{3}J_{HH}$ = 7.06 Hz, overlapping with hexane); 0.96 (12H, d, ^{i}Pr CH_3, $^{3}J_{HH}$ = 7.06 Hz, overlapping with hexane).

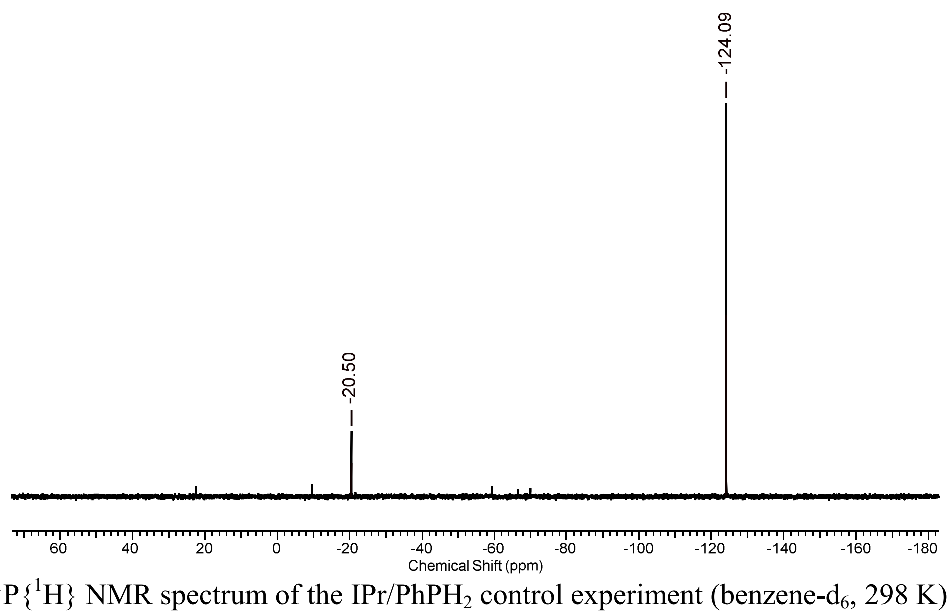

Figure S49. ^{31}P{^{1}H} NMR spectrum of the IPr/$PhPH_2$ control experiment (benzene-d_6, 298 K). δ/ppm: –20.5 (IPr·PPh); –124.1 ($PhPH_2$).

Control experiment: IMe_4 and $PhPH_2$. A solution of IMe_4 (0.006 g, 0.05 mmol) and $PhPH_2$ (0.07 ml of 10% w/w hexane solution, 0.05 mmol) in 1 ml benzene-d_6 was heated at 80°C for three days.

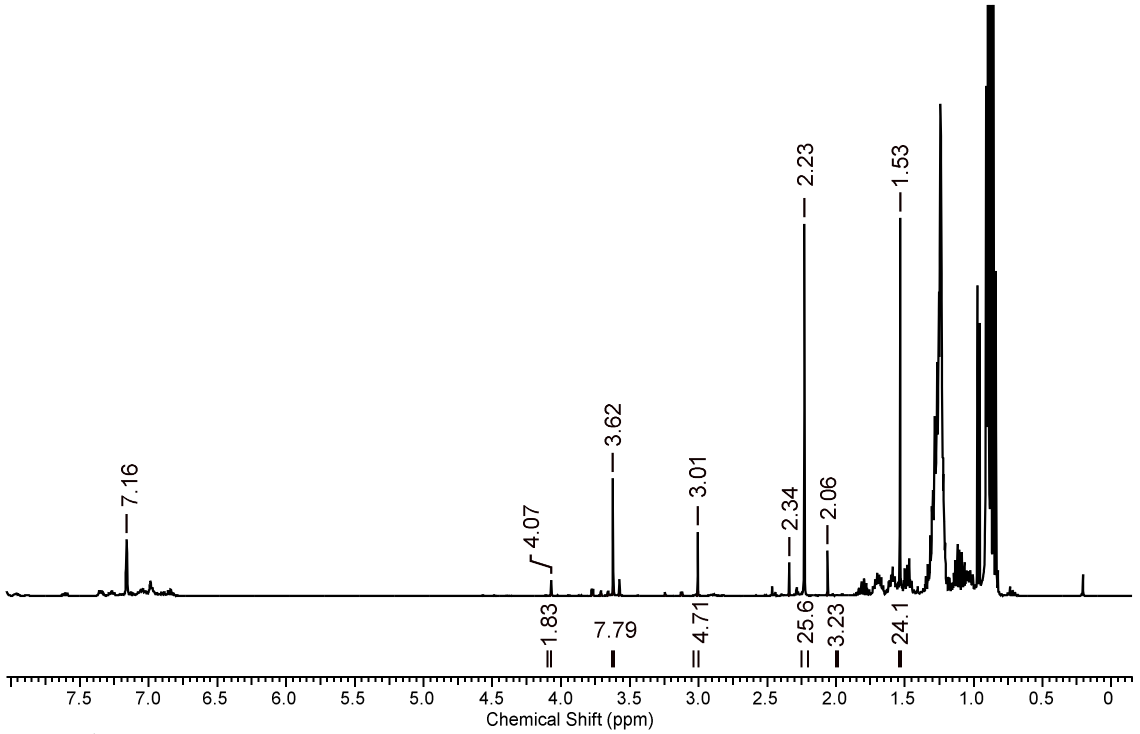

Figure S50. ^{1}H NMR spectrum of the IMe_4/$PhPH_2$ control experiment (400 MHz, benzene-d_6, 298 K). δ/ppm. **$PhPH_2$**: 7.25-7.29 (2H, m, PPh); 6.98-7.00 (3H, m, PPh); 3.83 (2H, d, PH_2, $^{1}J_{PH}$ = 198.99 Hz); 2.0-0.7 (hexane). **IMe_4·PPh**: 6.9-7.3 (5H, m, PPh); 2.23 (6H, s, NMe); 1.25 (6H, s, CCMe). **IMe_4H_2**: 3.62 (2H, s, NCH_2N); 2.23 (6H, s, NMe); 1.53 (6H, s, CMe).

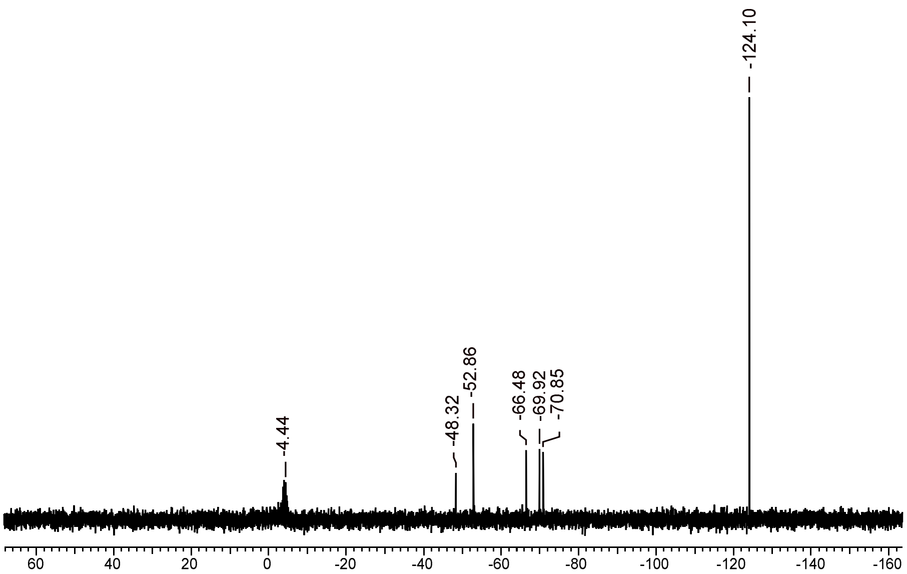

Figure S51. ^{31}P{^{1}H} NMR spectrum of the IMe_4/$PhPH_2$ control experiment (benzene-d_6, 298 K). δ/ppm: –4.4 (P_5Ph_5); –48.3 (P_4Ph_4); –52.9 (IMe_4·PPh); –66.5 to –70.9, (PhP(H)P(H)Ph); –124.1 ($PhPH_2$).

Preparative-scale catalytic synthesis of IMes·PMes (3) using 10 mol % 1-Co. A solution of IMes (0.15 g, 0.5 mmol) and $[Co(N(SiMe_3)_2)_2]_2$ (0.019 g, 0.025 mmol) in toluene (3 ml) was stirred at room temperature for 30 minutes. A solution of $MesPH_2$ in toluene (0.1 ml, 0.5 mmol) was added to the reaction mixture and heated at 80°C for 7 days. The volatiles were removed *in vacuo* and the remaining solids were washed with hexane (2 ml) to give **3** (0.12g, 51%), which was found to be pure by ^1H and ^{31}P NMR spectroscopy (see Figures S22 and S23 for ^1H and ^{31}P NMR spectra).

NMR-scale catalytic synthesis of IMes·PMes (3) using 10 mol % 1-Co. A mixture of IMes (0.015 g, 0.05 mmol), $[Co\{N(SiMe_3)_2\}_2]_2$ (0.0019 g, 0.0025 mmol) and $MesPH_2$ (0.5 ml, 0.05 mmol) in benzene-d_6 (1.0 ml) was heated to 80°C in an NMR tube for seven days.

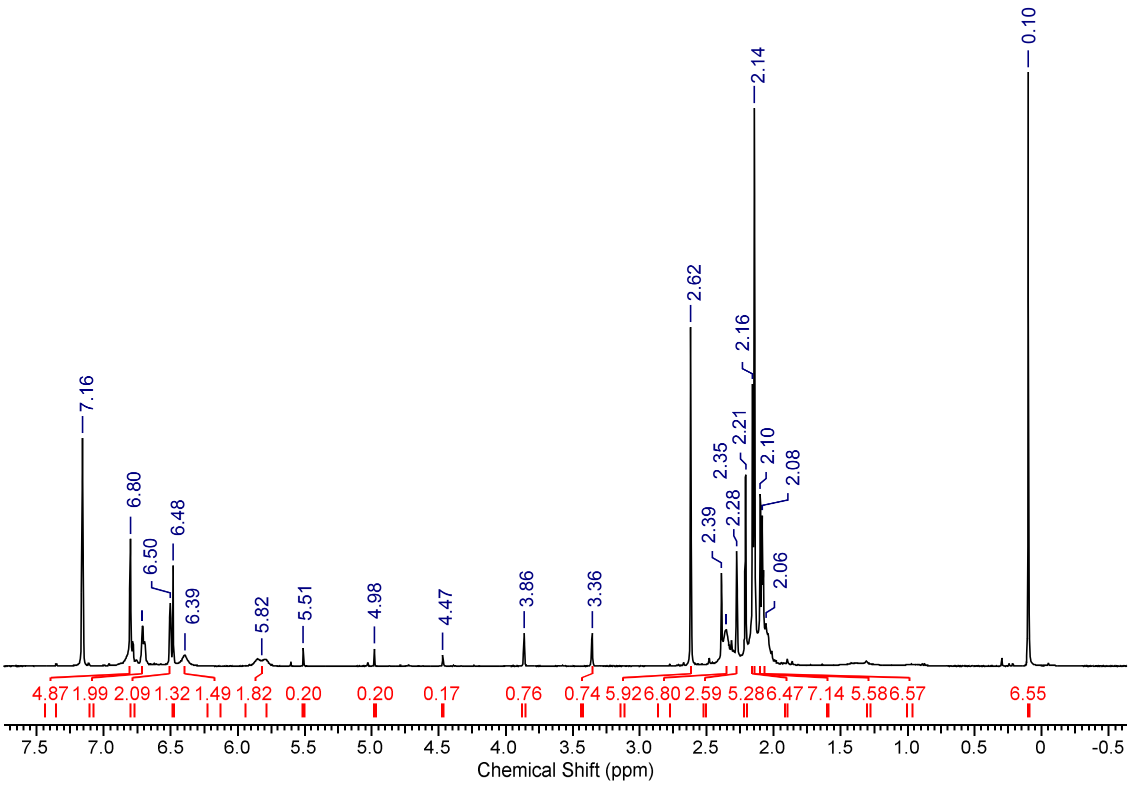

Figure S52. ^1H NMR spectrum of the reaction mixture for the synthesis of IMes·PMes (**3**) using 10 mol % [Co(N")$_2$] (benzene-d_6, 400 MHz, 298 K). δ/ppm. **IMes·PMes:** 6.80 (2H, s, mesityl *meta*-CH); 6.50 (2H, s, mesityl *meta*-CH); 6.39 (2H, s, mesityl *meta*-CH); 5.82 (2H, br d, imidazole-CH); 2.62 (6H, s, mesityl *ortho*-CH_3); 2.39 (6H, s, mesityl *ortho*-CH_3); 2.14 (6H, s, mesityl *ortho*-CH_3); 2.11 (6H, s, mesityl *para*-CH_3); 2.06(3H, s, mesityl *para*-CH_3). **$MesPH_2$:** 6.71 (2H, s, mesityl *meta*-CH); 3.61 (d, 2H, PH_2, $^1J_{PH}$ = 203.70 Hz); 2.21 (6H, s, mesityl *ortho*-CH_3); 2.08 (6H, s, mesityl *para*-CH_3). **IMes:** 6.80 (4H, s, mesityl *meta*-CH); 6.48 (2H, s, imidazolylidene CH); 2.16 (18H, mesityl CH_3).

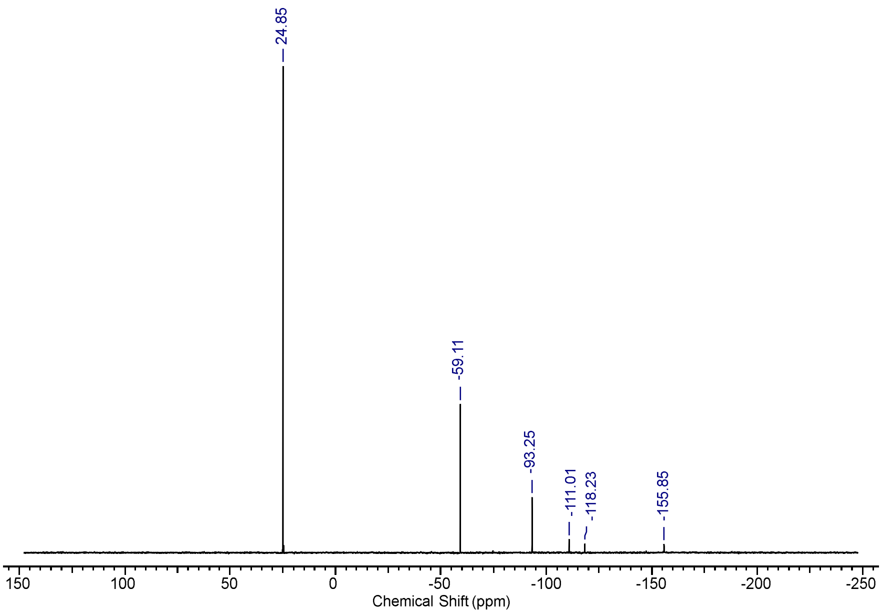

Figure S53. $^{31}P\{^1H\}$ NMR spectrum of the reaction mixture for the synthesis of IMes·PMes (**3**) using 10 mol % [Co(N")$_2$] (benzene-d$_6$, 298 K). δ/ppm: –59.1 (IMes·PMes); –93.3 (Mes$_2$PH); –111.0 (MesP(H)P(H)Mes); –118.2 (MesP(H)P(H)Mes); –155.9 (MesPH$_2$). δ = +24.9 ppm corresponds to a benzene solution of Ph$_3$P=O contained in a capillary.

Attempted catalytic synthesis of IPr·PMes (4) using 10 mol % [Co(N")$_2$]. A mixture of IPr (0.015 g, 0.05 mmol), [Co{N(SiMe$_3$)$_2$}$_2$]$_2$ (0.0019 g, 0.0025 mmol) and MesPH$_2$ (0.5 ml, 0.05 mmol) in benzene-d$_6$ (1.0 ml) was heated to 80°C in an NMR tube for seven days.

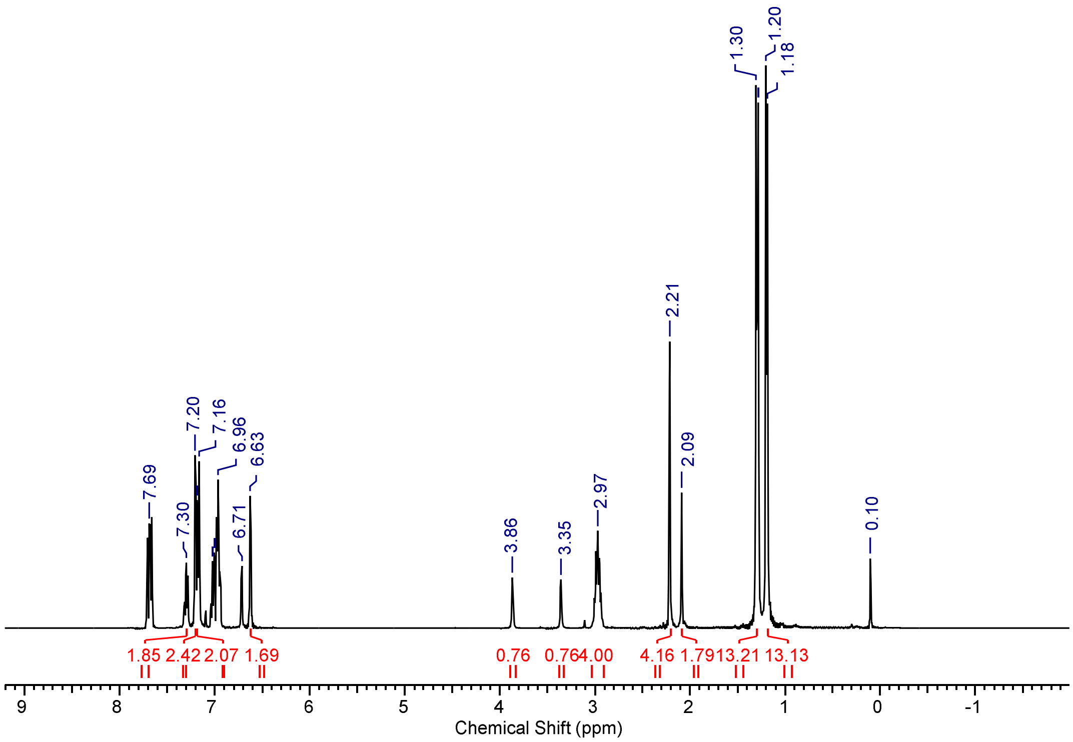

Figure S54. ^1H NMR spectrum of the reaction mixture for the synthesis of IPr·PMes (**4**) using 10 mol % [Co(N")$_2$] (benzene-d$_6$, 400 MHz, 298 K). δ/ppm. **IPr:** 7.30 (2H, t, Dipp *para*-CH, $^3J_{HH}$ = 7.46 Hz); 7.20 (4H, d, Dipp *meta*-CH, $^3J_{HH}$ = 7.38 Hz); 6.63 (2H, s, imidazole CH); 2.97 (4H, sept, isopropyl CH, $^3J_{HH}$ = 7.11 Hz); 1.29 (12H, d, isopropyl CH$_3$, $^3J_{HH}$ = 6.94 Hz); 1.19 (12H, d, isopropyl CH$_3$, $^3J_{HH}$ = 6.94 Hz). **MesPH$_2$:** 6.71 (2H, s, mesityl *meta*-CH); 3.61 (d, 2H, PH$_2$, $^1J_{PH}$ = 201.52 Hz); 2.21 (6H, s, mesityl *ortho*-CH$_3$); 2.09 (6H, s, mesityl *para*-CH$_3$). Ph$_3$PO capillary (7.69, 7.04, 6.99).

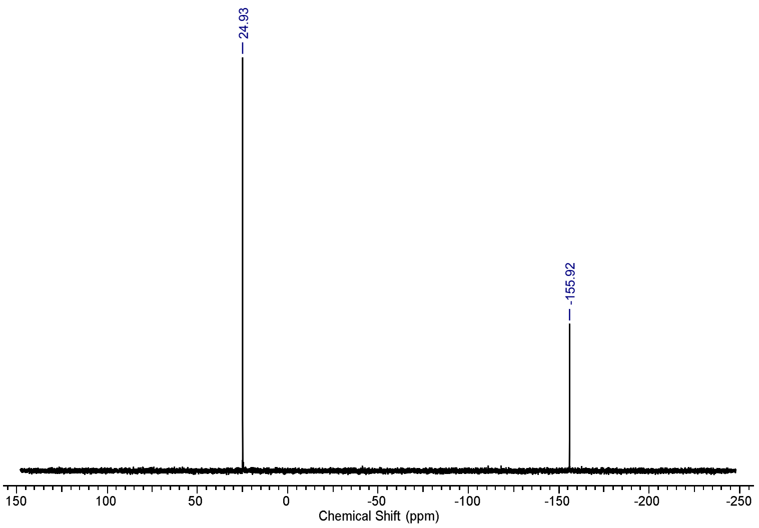

Figure S55. $^{31}P\{^1H\}$ NMR spectrum of the reaction mixture for the synthesis of IPr·PMes (**4**) using 10 mol % [Co(N")$_2$] (benzene-d$_6$, 298 K). δ/ppm: –155.9 ($MesPH_2$). δ = +24.9 ppm corresponds to a benzene solution of $Ph_3P{=}O$ contained in a capillary.

Preparative-scale catalytic synthesis of IMe$_4$·PMes (5) using 10 mol % 1-Co. A solution of IMe$_4$ (0.062 g, 0.5mmol) in toluene (3 ml) was added to a solution of [Co{N(SiMe$_3$)$_2$}$_2$]$_2$ (0.0019 g, 0.025 mmol) in toluene (3 ml) at room temperature and the mixture was stirred for 30 minutes. A solution of $MesPH_2$ (76 µL, 0.50 mmol) in toluene (1 ml) was added to the reaction mixture, which was stirred at 80°C for seven days. The volatiles were removed and the product extracted with hexane, concentrated, and stored at –28°C overnight, which produced yellow crystals. The residual solvent was decanted, the crystals were washed in pentane and then dried *in vacuo*. IMe$_4$·PMes was isolated as pale yellow crystals (0.044 g, 32%). Anal. Calcd. for $C_{16}H_{23}N_2P$: C, 70.05; H, 8.45; N, 10.21, Found: C, 69.84; H, 8.34; N, 10.43.

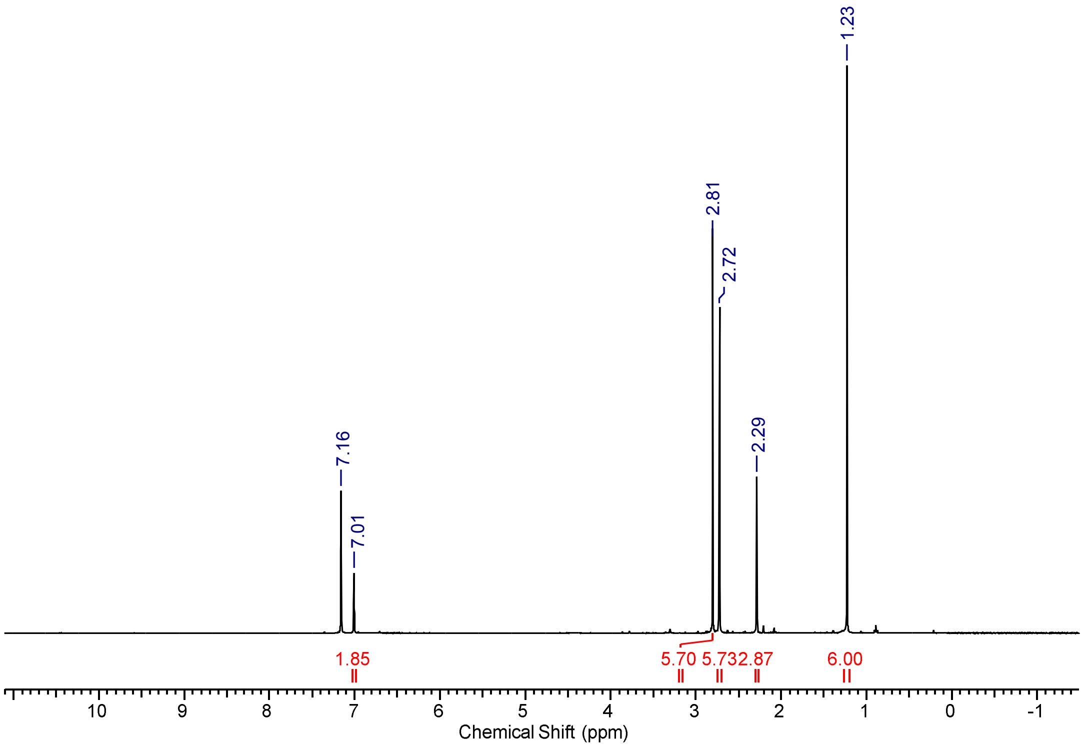

Figure S56. ^1H NMR spectrum of IMe$_4$·PMes (**5**) (benzene-d$_6$, 400 MHz, 298 K). δ/ppm: 7.01 (2H, s, mesityl *meta*-CH); 2.81 (6H, s, NCH_3); 2.72 (6H, s, mesityl *ortho*-CH_3); 2.29 (3H, s, mesityl *para*-CH_3); 1.23 (6H, s, IMe$_4$ CCH_3).

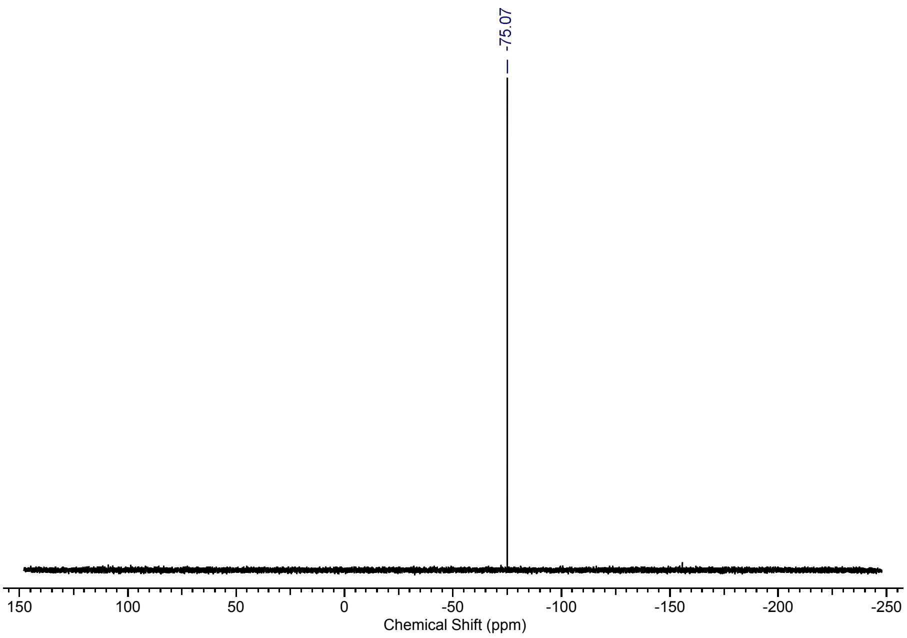

Figure S57. $^{31}P\{^1H\}$ NMR spectrum of $IMe_4{\cdot}PMes$ (**5**) (benzene-d_6, 298 K). δ/ppm: –75.1.

NMR-scale catalytic synthesis of $IMe_4{\cdot}PMes$ (5) using 10 mol % 1-Co. A mixture of IMe_4 (0.015 g, 0.05 mmol), $[Co\{N(SiMe_3)_2\}_2]_2$ (0.0019 g, 0.0025 mmol) and $MesPH_2$ (76 μL, 0.50 mmol) in benzene-d6 (1.0 ml) was heated to 80°C in an NMR tube for seven days.

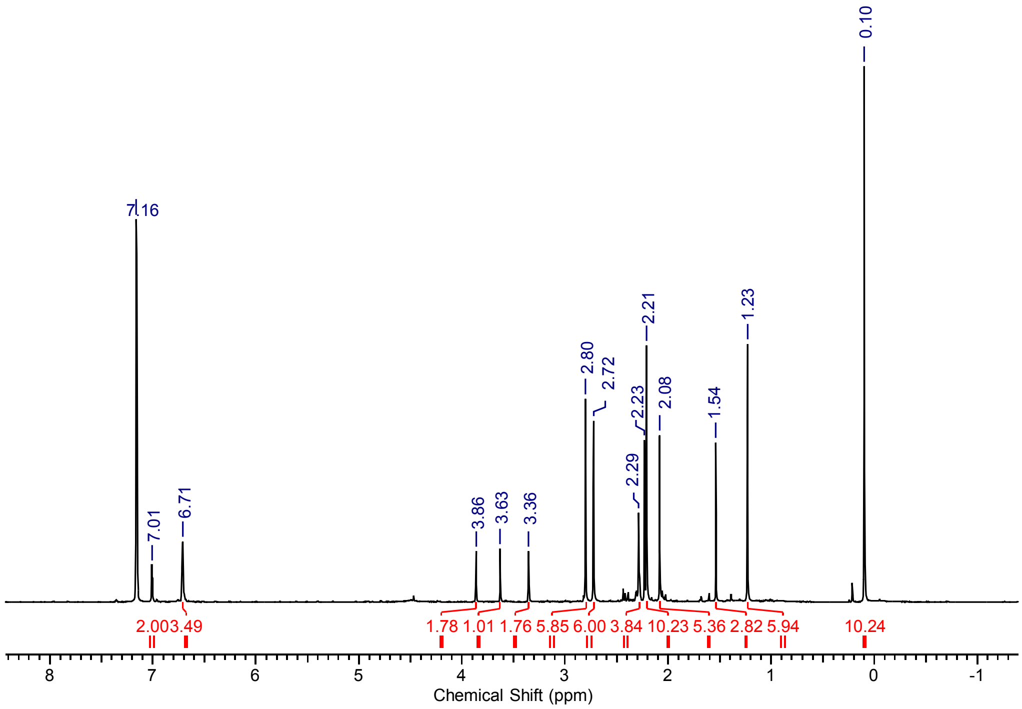

Figure S58. ^1H NMR spectrum of the reaction mixture for the catalytic synthesis of $IMe_4{\cdot}PMes$ (**5**) using 10 mol % $[Co(N'')_2]$ (benzene-d_6, 400 MHz, 298 K). δ/ppm. **$IMe_4{\cdot}PMes$**: 7.01 (2H, s, mesityl *meta*-CH); 2.81 (6H, s, NCH_3); 2.72 (6H, s, mesityl *ortho*-CH_3); 2.29 (3H, s, mesityl *para*-CH_3); 1.23 (6H, s, IMe CCH_3). **IMe_4**: 3.36 (6H, s, NCH_3); 1.54 (6H, s, CCH_3). **$MesPH_2$**: 6.71 (2H, s, mesityl *meta*-CH); 3.61 (d, 2H, PH_2, $^1J_{PH}$ = 202.47 Hz); 2.21 (6H, s, mesityl *ortho*-CH_3); 2.08 (6H, s, mesityl *para*-CH_3).

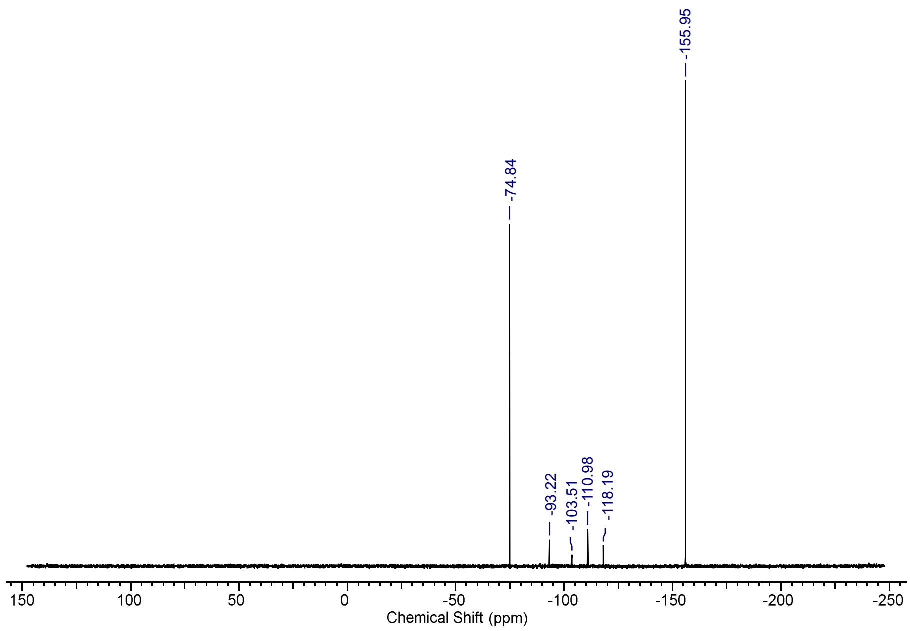

Figure S59. $^{31}P\{^1H\}$ NMR spectrum of the reaction mixture for the catalytic synthesis of $IMe_4{\cdot}PMes$ (**5**) using 10 mol % **1-Co** (benzene-d_6, 298 K). δ/ppm: –74.8 ($IMe_4{\cdot}PMes$); –93.2 (Mes_2PH); –103.5 (s, Mes_2PH); –111.0 (MesP(H)P(H)Mes); –118.2 (MesP(H)P(H)Mes); –155.8 ($MesPH_2$).

Preparative-scale catalytic synthesis of IMes·PPh (6) using 10 mol % 1-Co. IMes (0.15 g, 0.5 mmol) and $[Co(N(SiMe_3)_2)_2]_2$ (0.019 g, 0.025 mmol) were charged into an ampoule and dissolved in toluene (3 ml). The reaction mixture was stirred at room temperature for 30 minutes. A solution of $PhPH_2$ in hexane (0.75 ml, 0.05mmol) was added to the reaction mixture, which was heated at 80°C for 2 hours. The volatiles were removed *in vacuo* and the remaining solids were washed with hexane (2 ml) to give **6** as a bright yellow (0.12 g, 58%). Purity was established by comparison of the ^1H and ^{31}P NMR spectroscopy to those of the previously reported compound.[6]

Preparative-scale catalytic synthesis of IMes·PPh (6) using 1 mol % 1-Co. IMes (0.40 g, 1.3 mmol) and $[Co(N(SiMe_3)_2)_2]_2$ (0.005 g, 0.0065 mmol) were charged into an ampoule and dissolved in toluene (10 ml). The reaction mixture was stirred at room temperature for 30 minutes A solution of $PhPH_2$ in hexane (1.95 ml, 1.3 mmol) was added to the reaction mixture, which was heated at 80°C for 18. The volatiles were removed *in vacuo* and the remaining solids were washed with hexane (5 ml) to give **6** as a bright yellow solid (0.33 g, 61%). Purity was established by comparison of the ^1H and ^{31}P NMR spectroscopy to those of the previously reported compound.[6]

NMR-scale catalytic synthesis of IMes·PPh (6) using 10 mol % 1-Co. A mixture of IMes (0.015 g, 0.05 mmol), $[Co\{N(SiMe_3)_2\}_2]_2$ (0.0019 g, 0.0025 mmol) and $PhPH_2$ (10% w/w in hexane, 76 µL, 0.05 mmol) in benzene-d_6 (1.0 ml) was heated to 80°C in an NMR tube for seven days. Volatiles were removed *in vacuo* and the sample was dissolved in benzene-d_6.

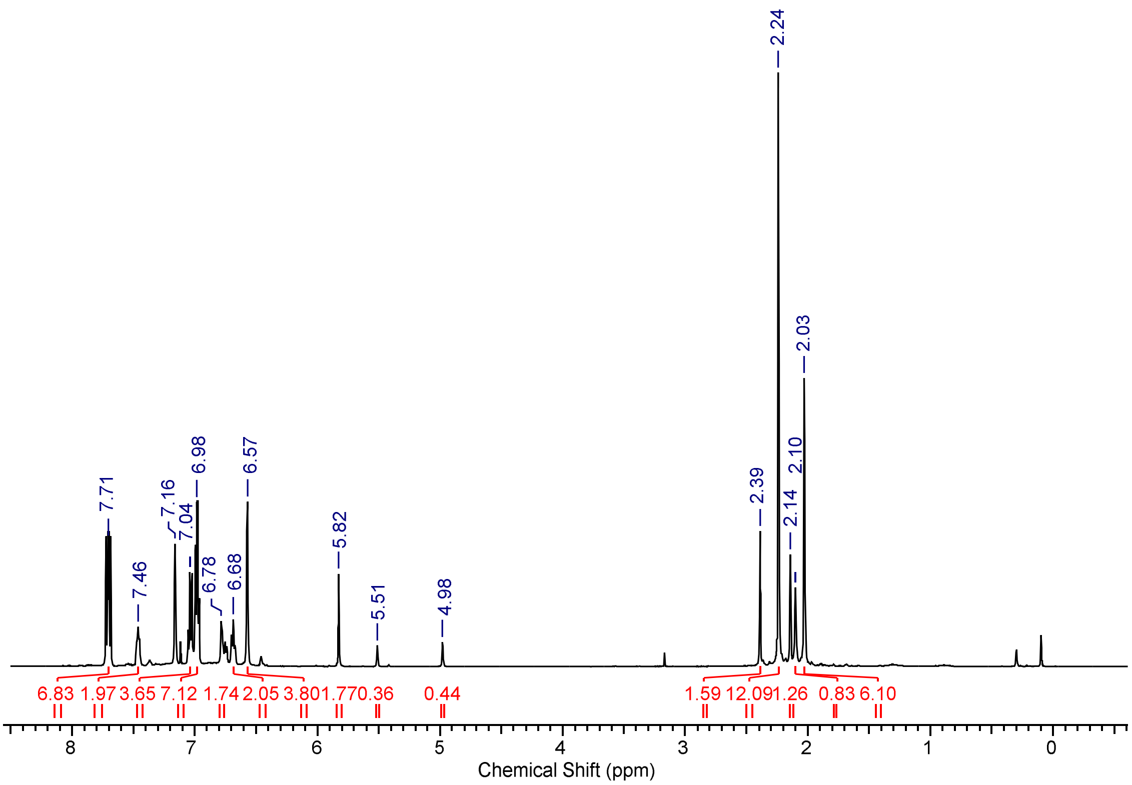

Figure S60. ^{1}H NMR spectrum of the reaction mixture for the catalytic synthesis of IMes·PPh (**6**) using 10 mol % [Co(N″)$_2$] (400 MHz, benzene-d$_6$, 298 K). δ/ppm: **IMes·PPh:** 7.47 (2H, m, phenyl *ortho*-CH); 6.68-6.78 (3H, m, phenyl *meta/para*-CH); 6.57 (4H, s, mesityl *meta*-CH); 5.82 (2H, s, imidazole-CH); 2.24 (12H, s, mesityl *ortho*-CH$_3$); 2.03 (6H, s, mesityl *para*-CH$_3$). **IMesH$_2$**: 6.78 (4H, s, mesityl *meta*-CH); 5.51 (2H, s, NCH$_2$N); 4.98 (2H, imidazole-CH); 2.39 (12H, s, mesityl *ortho*-CH$_3$), 2.14 (6H, s, mesityl *para*-CH$_3$). Ph$_3$PO capillary (7.71, 7.04, 6.98).

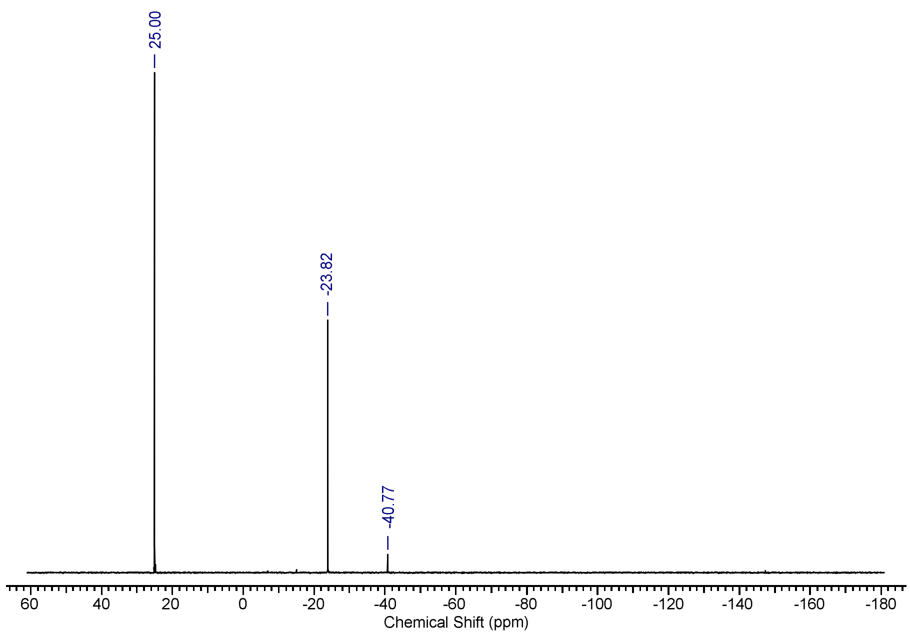

Figure S61. $^{31}P\{^{1}H\}$ NMR spectrum of the reaction mixture for the catalytic synthesis of IMes·PPh (**6**) using 10 mol % [Co(N″)$_2$] (benzene-d$_6$, 298 K). δ/ppm: –23.8 (IMes·PPh); –40.8 (PhPH$_2$). δ = +25.0 ppm corresponds to a benzene solution of Ph$_3$P=O contained in a capillary.

Preparative-scale catalytic synthesis of IPr·PPh (7) using 10 mol % [Co(N″)$_2$]. IPr (0.19 g, 0.5 mmol) and [Co(N(SiMe$_3$)$_2$)$_2$]$_2$ (0.019 g, 0.025 mmol) were dissolved in toluene (3 ml) and stirred at room temperature for 30 minutes. A solution of PhPH$_2$ in hexane (0.7 M, 0.75 ml, 0.5 mmol) was added to the reaction mixture and heated at 80°C for two hours. The volatiles were removed *in vacuo* and the remaining bright yellow solid washed with hexane (2 ml), which gave **7** as a bright yellow solid (0.33 g, 61%). Purity was established by comparison of the ^{1}H and ^{31}P NMR spectroscopy to those of the previously reported compound.[6]

NMR-scale catalytic synthesis of IPr·PPh (7) using 10 mol % 1-Co. A mixture of IPr (0.019 g, 0.05 mmol), [Co{N(SiMe$_3$)$_2$}$_2$]$_2$ (0.0019 g, 0.025 mmol) and PhPH$_2$ (7.6 μL, 0.05 mmol) in benzene-d$_6$ (1.0 ml) was heated to 80°C in an NMR tube for seven days. Volatiles were removed *in vacuo* and the sample was re-dissolved in benzene-d$_6$.

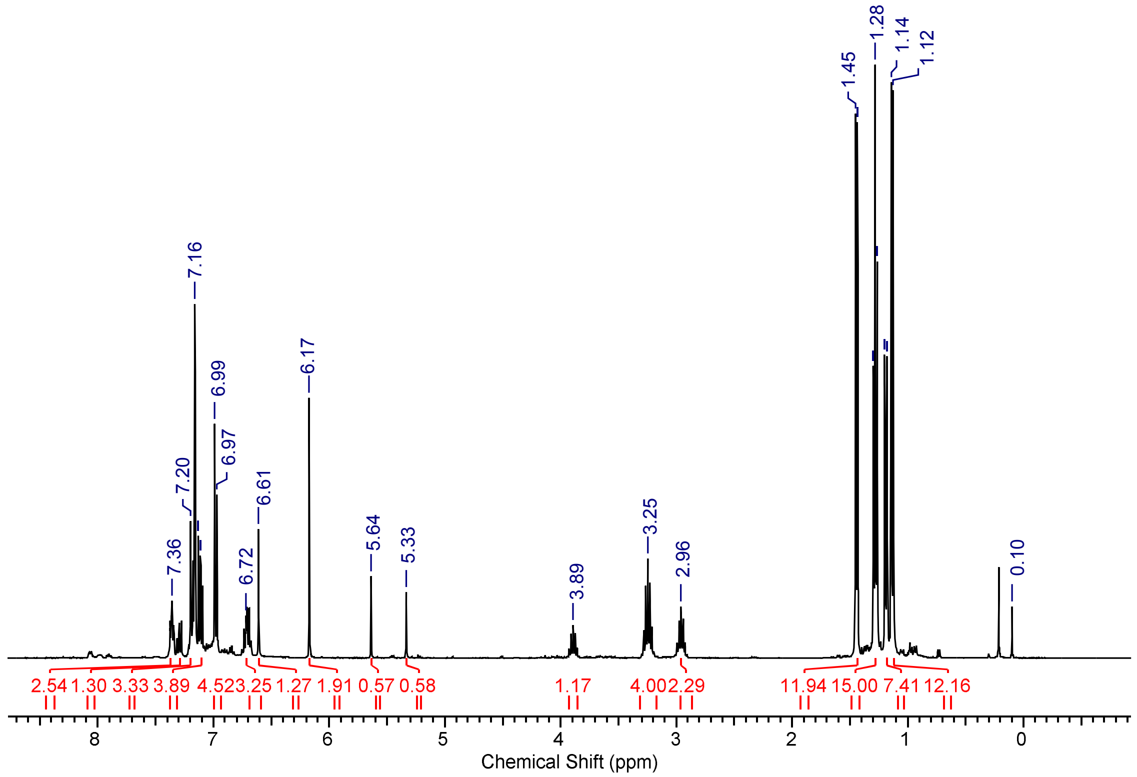

Figure S62. ^{1}H NMR spectrum of the reaction mixture for the catalytic synthesis of IPr·PPh (**7**) using 10 mol % [Co(N″)$_2$] (400 MHz, benzene-d$_6$, 298 K). δ/ppm. **IPr·PPh:** 7.36 (2H, m, phenyl *ortho*-CH); 7.10 (t, 2H, $^3J_{HH}$ = 6.77 Hz, Dipp *para*-CH); 6.98 (d, 4H, $^3J_{HH}$ = 7.63 Hz, Dipp *meta*-CH); 6.72 (m, phenyl *ortho*-CH); 6.17 (s, 2H, imidazole CH); 3.25 (4H, sept, $^3J_{HH}$ = 6.85 Hz, isopropyl CH); 1.45 (12H, d, $^3J_{HH}$ = 6.85 Hz, isopropyl-CH$_3$); 1.13 (12H, d, $^3J_{HH}$ = 6.97 Hz, isopropyl-CH$_3$). **IPr:** 7.30 (2H, t, $^3J_{HH}$ = 7.34 Hz, Dipp *para*-CH); 7.20 (4H, d, $^3J_{HH}$ = 8.02 Hz, Dipp *meta*-CH); 6.61 (2H, s, imidazole CH); 2.96 (4H, sept, $^3J_{HH}$ = 6.97 Hz, isopropyl-CH); 1.28 (12H, d, $^3J_{HH}$ = 6.85 Hz, isopropyl-CH$_3$); 1.19 (12H, d, $^3J_{HH}$ = 6.97 Hz, isopropyl-CH$_3$). **IPrH$_2$**: 7.12-7.09 (m, 2H, aryl CH); 6.67-6.71 (m, 4H, aryl CH); 5.64 (s, 2H, imidazole CH); 5.33 (s, 2H, NCH$_2$N); 3.89 (4H, sept, $^3J_{HH}$ = 6.97 Hz, isopropyl CH); 1.27 (12H, d, $^3J_{HH}$ = 6.85 Hz, isopropyl-CH$_3$).

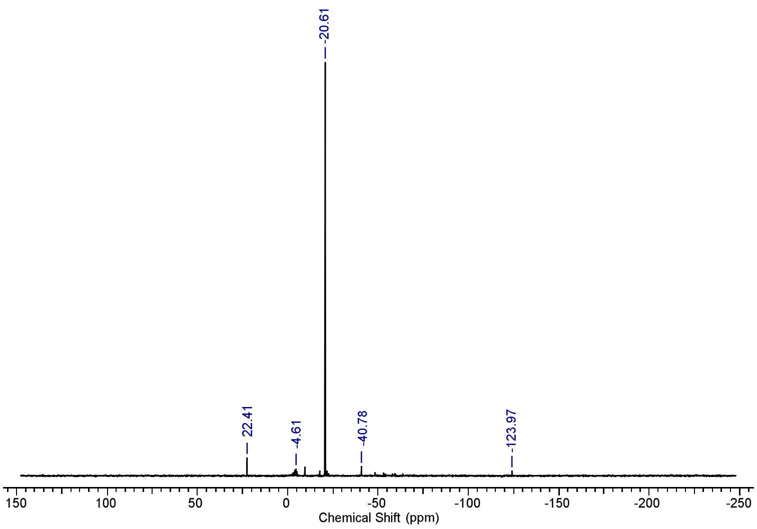

Figure S63. $^{31}P\{^1H\}$ NMR spectrum of the reaction mixture for the catalytic synthesis of IPr·PPh (**7**) using 10 mol % [Co(N″)$_2$] (benzene-d$_6$, 298 K). δ/ppm: +22.4 (unknown); –4.6 (Ph_5P_5); –20.6 (IPr·PPh); –40.8 (Ph_2PH); –123.97 ($PhPH_2$).

Catalytic synthesis of IMe$_4$·PPh (8) using 10 mol % [Co(N″)$_2$]. IMe$_4$ (0.062 g, 0.5 mmol) and [Co(N(SiMe$_3$)$_2$)$_2$]$_2$ (0.019 g, 0.025 mmol) were dissolved in toluene (3 ml) and stirred at room temperature for 30 minutes. A solution of $PhPH_2$ in hexane (0.7 M, 0.75 ml, 0.5 mmol) was added to the reaction mixture and heated at 80°C for four hours. The volatiles were removed *in vacuo* and the remaining bright yellow solid washed with hexane (2 ml), which gave **8** as a bright yellow solid (0.072 g, 62%). Purity was established by comparison of the ^1H and ^{31}P NMR spectroscopy to those of the previously reported compound.[6]

NMR-scale catalytic synthesis of IMe$_4$·PPh (7) using 10 mol % 1-Co. A mixture of IMe$_4$ (0.015 g, 0.05 mmol), [Co{N(SiMe$_3$)$_2$}$_2$]$_2$ (0.0019 g, 0.025 mmol) and MesPH$_2$ (0.7 M, 7.6 µL, 0.05 mmol) in benzene-d$_6$ (0.5 ml) was heated to 80°C in an NMR tube for seven days. Volatiles were removed in vacuo and the sample was dissolved in benzene-d$_6$.

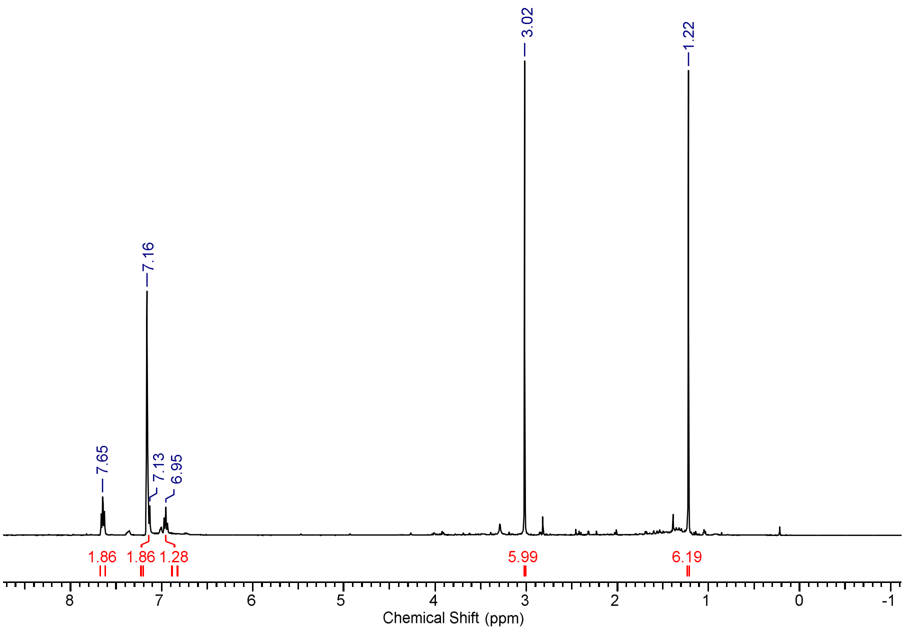

Figure S64. ^1H NMR spectrum of the reaction mixture for the catalytic synthesis of IMe$_4$·PPh (**8**) using 10 mol % [Co(N″)$_2$] (400 MHz, benzene-d$_6$, 298 K). δ/ppm: 7.65 (2H, m, phenyl *ortho*-CH); 7.13 (2H, m, phenyl *meta*-CH); 6.95 (1H, m, phenyl *para*-CH); 3.02 (6H, s, NCH_3); 1.22 (6H, s, CCH_3).

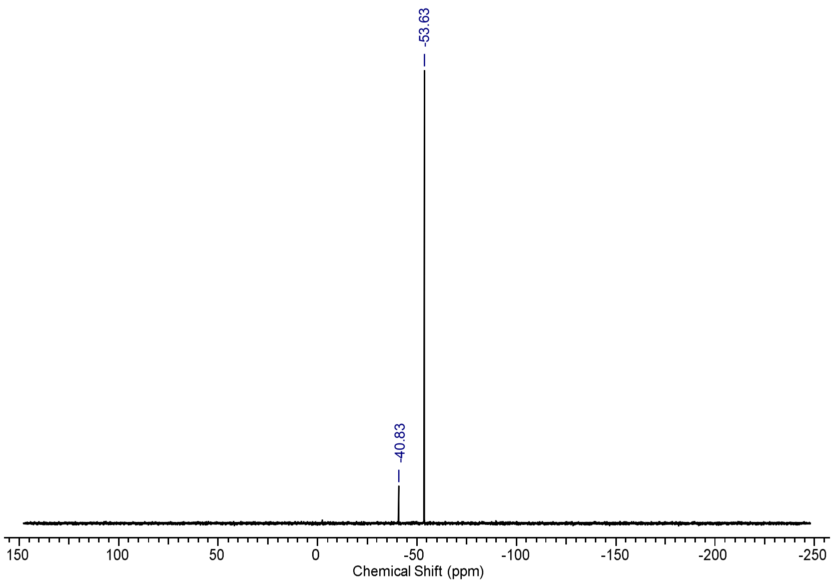

Figure S65. $^{31}P\{^1H\}$ NMR spectrum of the reaction mixture for the catalytic synthesis of $IMe_4{\cdot}PPh$ (**8**) using 10 mol % $[Co(N'')_2]$ (benzene-d_6, 298 K). δ/ppm: –40.8 ($PhPH_2$); –53.6 ($IMe_4{\cdot}PPh$).

NMR-scale catalytic synthesis of $IMe_4{\cdot}PMes$ (5) using 5 mol % 2-Fe. A mixture of IMe_4 (0.015 g, 0.05 mmol), $[Fe\{N(SiMe_3)_2\}_2]_2$ (0.0019 g, 0.025 mmol) and $MesPH_2$ (7.6 µL, 0.05 mmol) in benzene-d_6 (0.5 ml) was heated to 80°C in benzene-d_6 in an NMR tube for seven days.

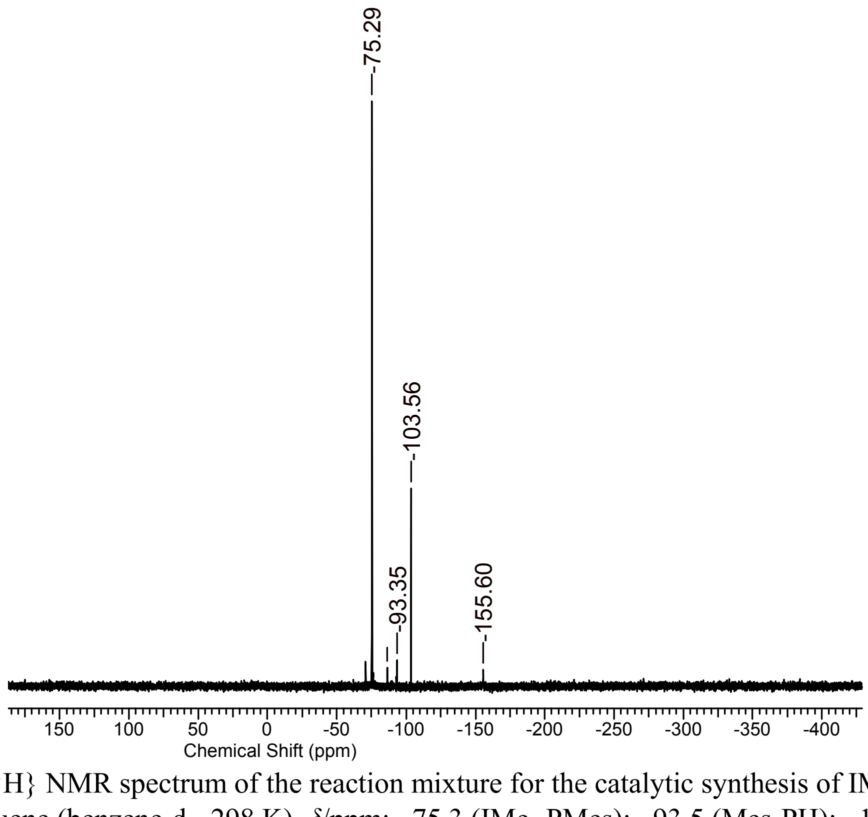

Figure S66. $^{31}P\{^1H\}$ NMR spectrum of the reaction mixture for the catalytic synthesis of $IMe_4{\cdot}PPh$ (**8**) using 5 mol % **2-Fe**·toluene (benzene-d_6, 298 K). δ/ppm: –75.3 ($IMe_4{\cdot}PMes$); –93.5 (Mes_2PH); –103.6 (MesMePH); –155.6 ($MesPH_2$).

X-ray crystallography

X-ray diffraction data for **1-Fe**, **1-Co**, **2-Fe.toluene**, **3** and **4** were collected on an Agilent Technologies SuperNova diffractometer with an Eos CCD detector using MoKα radiation. Data for **2-Co.toluene** were collected on a Bruker X8 Prospector 3-circle diffractometer with an APEX II CCD detector using CuKα radiation. Data for 7 were collected on an Oxford Diffraction Xcalibur diffractometer with a Sapphire 2 CCD detector. Crystals were covered in an inert oil and selected under a microscope. Structures were solved with SHELXS using direct methods and refined with SHELXL using least-squares minimisation.[7]

The structures of **2-Fe·toluene** and **2-Co·toluene** both contain a toluene molecule of solvation disordered over two positions and located on an inversion centre. This molecule was modelled free from special position constraints with 1/2 occupancy. The phenyl ring was modelled as a regular hexagon with the carbon atoms refined using SIMU and DELU restraints.

Table S1. Crystal data and structural refinement for compounds **1-M** and **2-M**·toluene.

	**1-Fe**	**1-Co**	**2-Fe·toluene**	**2-Co·toluene**
CCDC ref. code	1417243	1417244	1417245	1417246
Formula	$C_{19}H_{48}FeN_4Si_4$	$C_{19}H_{48}CoN_4Si_4$	$C_{53}H_{78}Fe_2N_8P_2$	$C_{53}H_{78}Co_2N_8P_2$
FW	500.82	503.90	1000.87	1007.03
Crystal system	Orthorhombic	Orthorhombic	Triclinic	Triclinic
Space group	*Pbcn*	*Pbcn*	*P*-1	*P*-1
a/Å	12.4515(13)	12.5808(6)	11.5890(7)	11.3790(9)
b/Å	12.2768(12)	12.2966(6)	11.6934(8)	11.7789(11)
c/Å	19.2749(14)	19.1395(7)	12.0250(7)	11.8321(12)
α/°	90	90	89.226(5)	88.523(8)
β/°	90	90	65.098(6)	64.402(9)
γ/°	90	90	67.294(6)	68.750(8)
V/Å^3	2946.4(5)	2960.9(2)	1341.71(17)	1316.7(2)
Z	4	4	1	1
Crystal size/mm^3	0.1 × 0.06 × 0.06	0.4 × 0.2 × 0.1	0.15 × 0.1 × 0.03	0.1 × 0.02 × 0.02
2θ range/°	6.638 to 50.7	6.626 to 52.742	6.83 to 52.744	8.152 to 133.15
Reflections collected	7285	10311	9814	11291
Independent reflections, R(int)	2693, 0.0647	3016, 0.0440	5473, 0.0330	4462, 0.0548
Completeness/%	99.8	99.7	99.8	95.7
Data/restraints/parameters	2693/0/136	3016/0/136	5473/57/325	4462/57/325
Goodness-of-fit on F^2	1.014	1.043	1.056	1.032
Final R indices [I>2σ(I)]	R_1 = 0.0510, wR_2 = 0.0853	R_1 = 0.0397, wR_2 = 0.0924	R_1 = 0.0506, wR_2 = 0.1065	R_1 = 0.0477, wR_2 = 0.1146
R indices (all data)	R_1 = 0.0982, wR_2 = 0.0998	R_1 = 0.0598, wR_2 = 0.1007	R_1 = 0.0682, wR_2 = 0.1187	R_1 = 0.0636, wR_2 = 0.1227

Table S2. Selected bond lengths [Å] and angles [°] for **1-Fe** and **1-Co**.

	**1-Fe**	**1-Co**
M–C	2.144(5)	2.083(4)
M–N	1.953(3)	1.951(2)
C(1)-M-N(2)	116.43(8)	117.29(6)
N(2)-M-N(2A)	127.15(16)	125.42(12)

Table S3. Crystal data and structural refinement for compounds **3** and **4**.

	**3 (IMes·PMes)**	**4 (IPr·PMes)**
CCDC ref. code	1417247	1417248
Formula	$C_{30}H_{35}N_2P$	$C_{36}H_{47}N_2P$
FW	454.57	538.72
Crystal system	Triclinic	Monoclinic
Space group	P-1	$P2_1/n$
a/Å	7.7120(5)	12.442(3)
b/Å	8.3117(5)	14.8946(10)
c/Å	20.2210(12)	17.789(7)
α/°	80.699(5)	90
β/°	81.673(5)	101.84(4)
γ/°	86.002(5)	90
V/Å^3	1264.21(14)	3226.5(16)
Z	2	4
Crystal size/mm^3	0.1 × 0.1 × 0.05	0.3 × 0.2 × 0.2
2θ range/°	6.93 to 51.36	5.844 to 52.744
Reflections collected	7767	12557
Independent reflections, R(int)	4753, 0.0318	6591, 0.0564
Completeness/%	99.1	99.9
Data/restraints/parameters	4753/0/307	6591/0/363
Goodness-of-fit on F^2	1.043	1.030
Final R indices [I>2σ(I)]	R_1 = 0.0569, wR_2 = 0.1240	R_1 = 0.0590, wR_2 = 0.1239
R indices (all data)	R_1 = 0.0831, wR_2 = 0.1404	R_1 = 0.0968, wR_2 = 0.1465

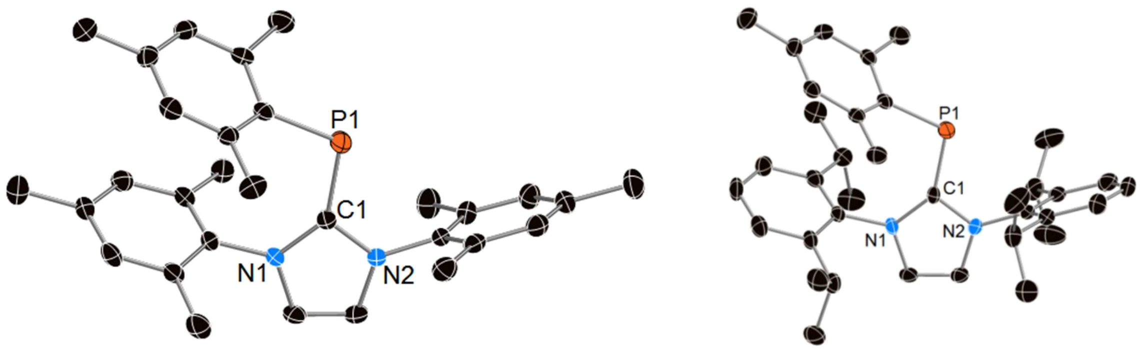

Figure S67. Molecular structures of **3** (left) and **4** (right) (thermal ellipsoids at 50% probability). Hydrogen atoms are not shown. Unlabelled atoms are carbon.

References

1. Andersen, R. A.; Faegri, K.; Green, J. C.; Haaland, A.; Lappert, M. F.; Leung, W.-P.; Rypdal, K. *Inorg. Chem*. **1988**, *27*, 1782.
2. Bryan, A. M.; Long, G. J.; Grandjean, F.; Power, P. P. *Inorg. Chem*. **2013**, *52*, 12152.
3. Kuhn, N.; Kratz, T. *Synthesis*, **1993**, 561.
4. Bantrelli, X.; Nolan, S. P. *Nature Protocols*, **2011**, *6*, 69.
5. Barlett, R. A.; Olmstead, M. M.; Power, P. P. *Inorg. Chem*. **1987**, *26*, 1941.
6. (a) Back, O.; Henry-Ellinger, M.; Martin, C. D.; Martin, D.; Bertrand, G. *Angew. Chem. Int. Ed*. **2013**, *52*, 2939. (b) Arduengo, A. J., III; Calabrese, J. C.; Cowley, A. H.; Rasika Dias, H. V.; Goerlich, J. R.; Marshall, W. J.; Riegel, B. *Inorg. Chem*. **1997**, *36*, 2151.
7. Sheldrick, G. M. *Acta Cryst.* **2008**, *A64*, 112.
